# Supplementary material for: Reconstitution of the destruction complex defines roles of AXIN polymers and APC in β-catenin capture, phosphorylation, and ubiquitylation
Source: Mol Cell. 2021 Aug 19;81(16):3246–3261.e11. doi: 10.1016/j.molcel.2021.07.013 (PMC8403986; doi:10.1016/j.molcel.2021.07.013)
Supplement: Document S1. Figures S1–S8 and Tables S1–S11 [file mmc1.pdf]

Molecular Cell, Volume *81*

Supplemental information

Reconstitution of the destruction complex defines roles of AXIN polymers and APC in β-catenin capture, phosphorylation, and ubiquitylation

Michael Ranes, Mariola Zaleska, Saira Sakalas, Ruth Knight, and Sebastian Guettler

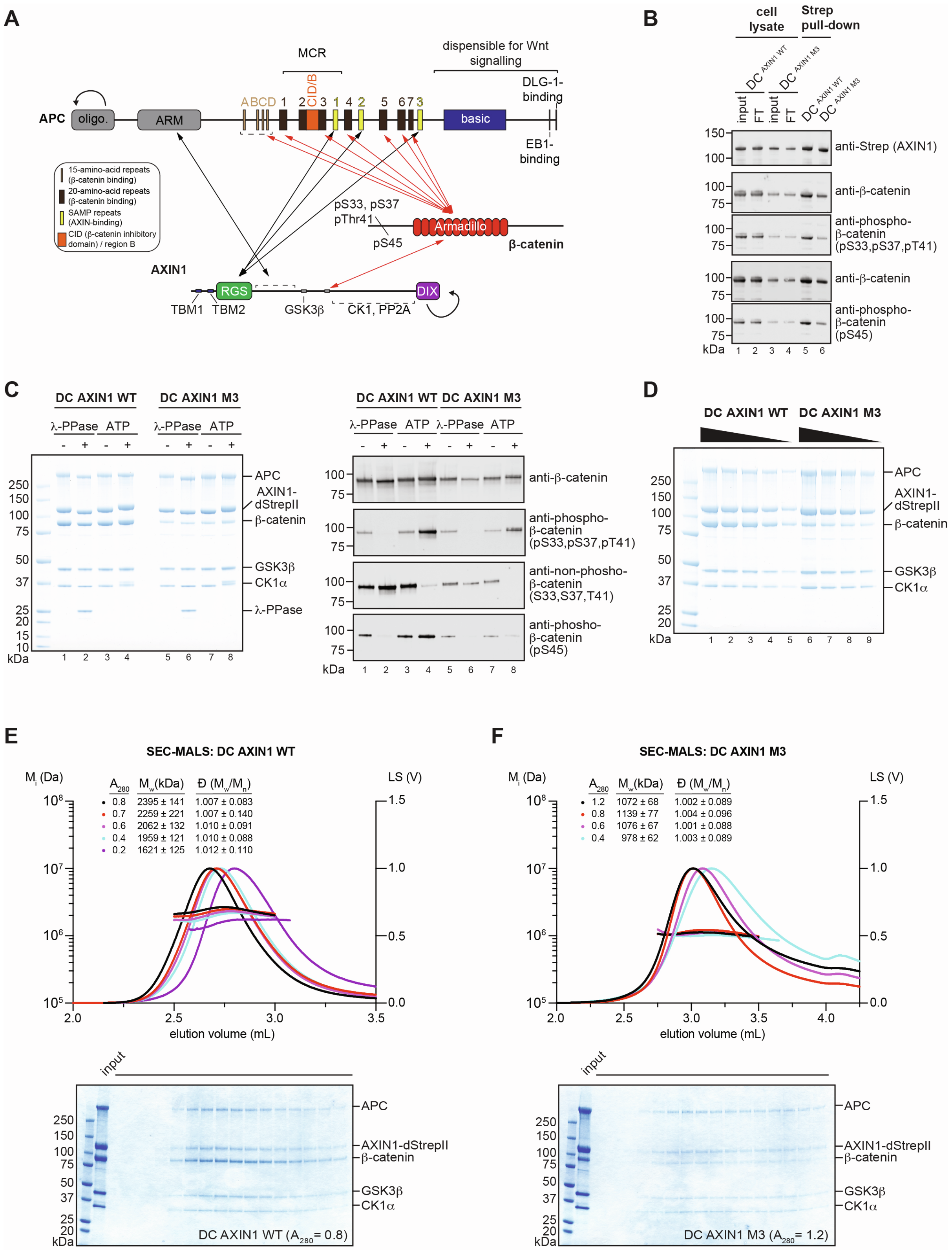

Figure S1. AXIN1 polymerisation is a major contributor to the concentration-dependent stoichiometry of the DC. (related to Figure 1)

(**A**) Schematic representation of core DC components. **APC:** APC 15- and 20-amino-acid-repeats (15R and 20R) bind β-catenin (except for 20R2) (Eklof Spink et al., 2001; Ha et al.,

2004). SAMP repeats bind AXIN1/2 (Spink et al., 2000). 20R2 and the adjacent β-catenin-inhibitory domain (CID) (a.k.a. region B) are proposed to bind α-catenin or regulate AXIN1/2 binding to APC (Choi et al., 2013; Pronobis et al., 2015). The MCR (mutation cluster region) refers to a CRC mutation hotspot (Kohler et al., 2008). Oligo., oligomerisation domain; ARM, armadillo repeat domain. **Beta-catenin:** ARM, armadillo repeat domain. **AXIN1:** TBM, tankyrase-binding motif (Morrone et al., 2012); RGS, regulator of G-protein signalling domain; DIX, polymerising domain present in Dishevelled and AXIN (Fiedler et al., 2011), sometimes also referred to as DAX domain. Kinase binding sites in AXIN1 (Stamos and Weis, 2013) are indicated. Mutual interaction sites between APC, AXIN1 and β-catenin are indicated by arrows. (**B**) Immunoblot analysis of the expressed and affinity-purified recombinant DC containing either wild-type or polymerisation-deficient (M3) AXIN1 to probe the β-catenin phosphorylation status. The figure, which corresponds to Figure 1B, shows one of three representative experiments that form the basis of Figure 1C. Input, lysates; FT, flow-through. (**C**) Further analysis of the phosphorylation status of the affinity-purified recombinant DC containing either wild-type or polymerisation-deficient (M3) AXIN1. Samples were either left untreated, incubated with λ-phosphatase for de-phosphorylation, or incubated with ATP for hyperphosphorylation. Left, Coomassie-stained SDS-PAGE gel; right, immunoblot analysis of the same samples. (**D**) Coomassie-stained SDS-PAGE gel of the indicated input samples analysed by SEC-MALS, corresponding to Figure 1D. (**E** and **F**) SEC-MALS analyses of the DC containing either wild-type AXIN1 (panel E) or polymerisation-deficient AXIN1 M3 (panel F), corresponding to Figure 1D. Chromatograms show plots of light scattering intensity at 90° and calculated molecular weights per measurement slice (M_i). The tables show the measured A_{280} of the input material*, weight-average molecular weights (M_w) and dispersities (Đ) over the peak areas with uncertainties (see Methods). Eluates across the peaks were fractionated and analysed by SDS-PAGE and Coomassie staining.

*Note that in the absence of a known stoichiometry, the concentration of the DC cannot be reliably calculated.

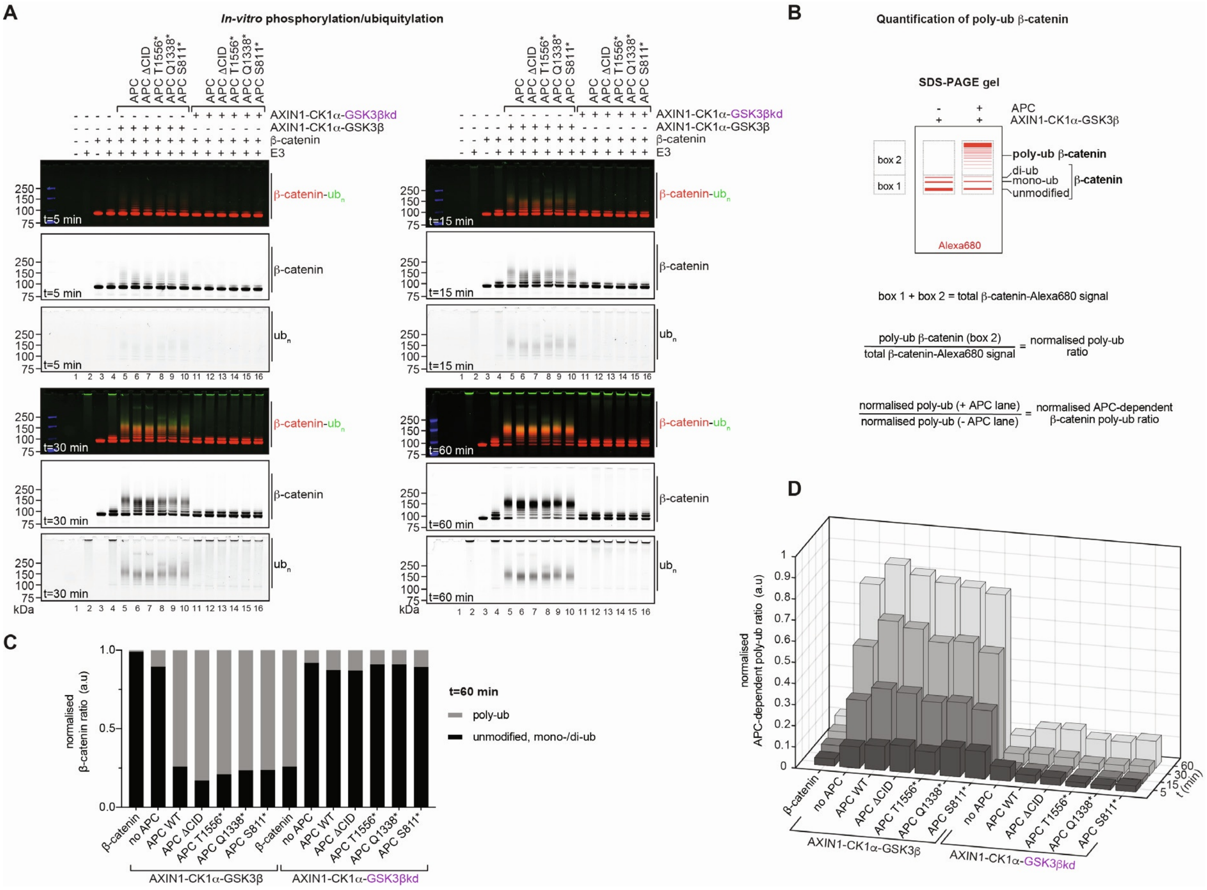

Figure S2. Reconstitution of the biochemical activity of the DC: β-catenin poly-ubiquitylation. (related to Figure 3)

(**A**) *In-vitro* DC activity assay as in Figure 3D, with different reaction times. The data for the 60-min time point are identical to those shown in Figure 3D and are included for comparison. (**B**) Procedure for quantifying β-catenin poly-ubiquitylation and the contribution of APC. (**C**) Normalised ratio of unmodified + mono-/di-ubiquitylated vs. poly-ubiquitylated β-catenin for the 60-min time point. (**D**) Normalised APC-dependent β-catenin poly-ubiquitylation, calculated for different reaction time points, as shown in A and B (n=1 representative experiment).

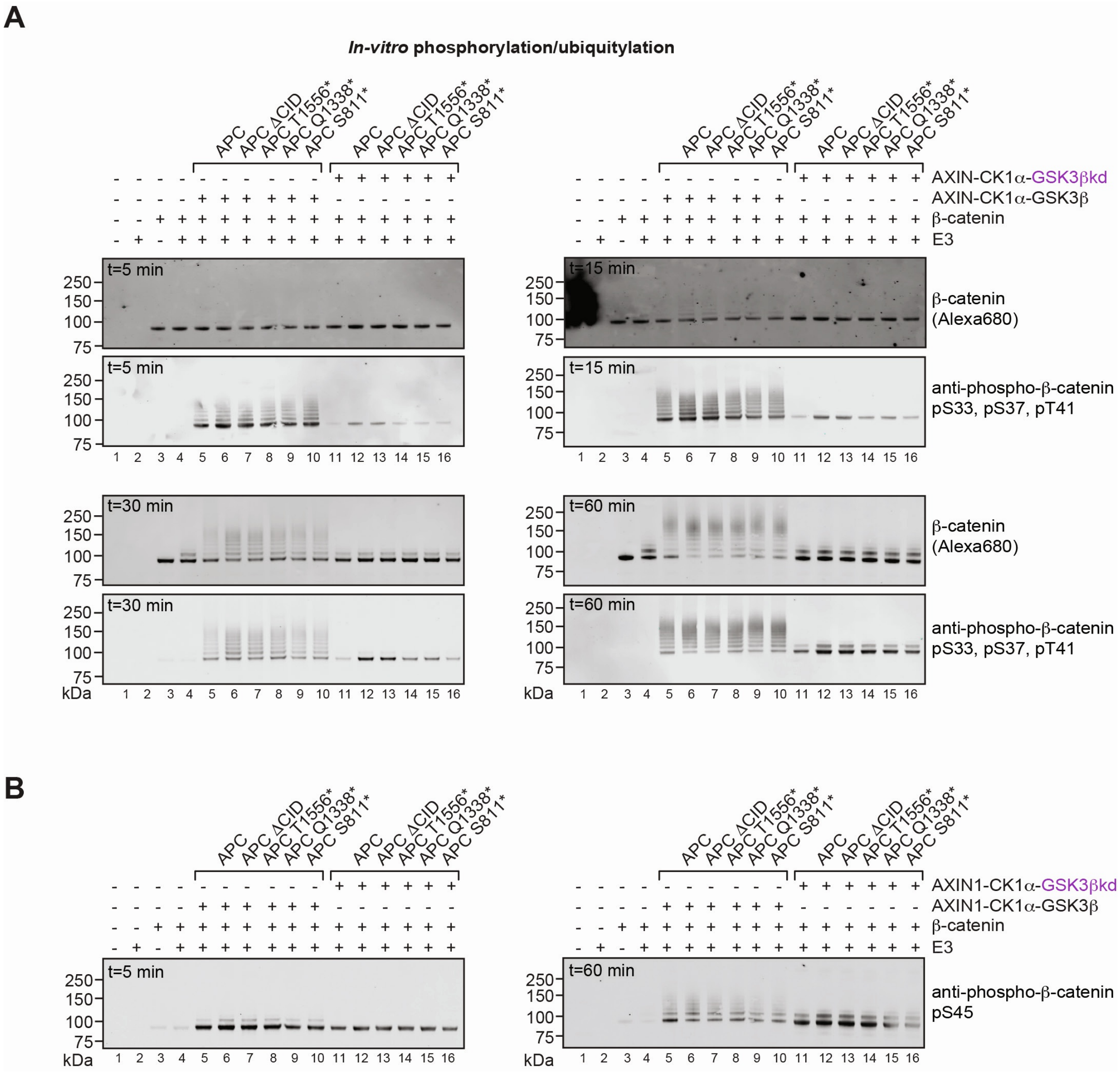

Figure S3. Reconstitution of the biochemical activity of the DC: β-catenin phosphorylation. (related to Figure 3)

(**A**) Analysis of GSK3β-dependent phosphorylation of β-catenin in the samples also analysed in Figures 3D and S2. The same gels as those shown in Figure 3D and S2A were used for transfer and immunoblotting. (**B**) Analysis of CK1α-dependent phosphorylation of β-catenin in the same samples from the indicated reaction time points.

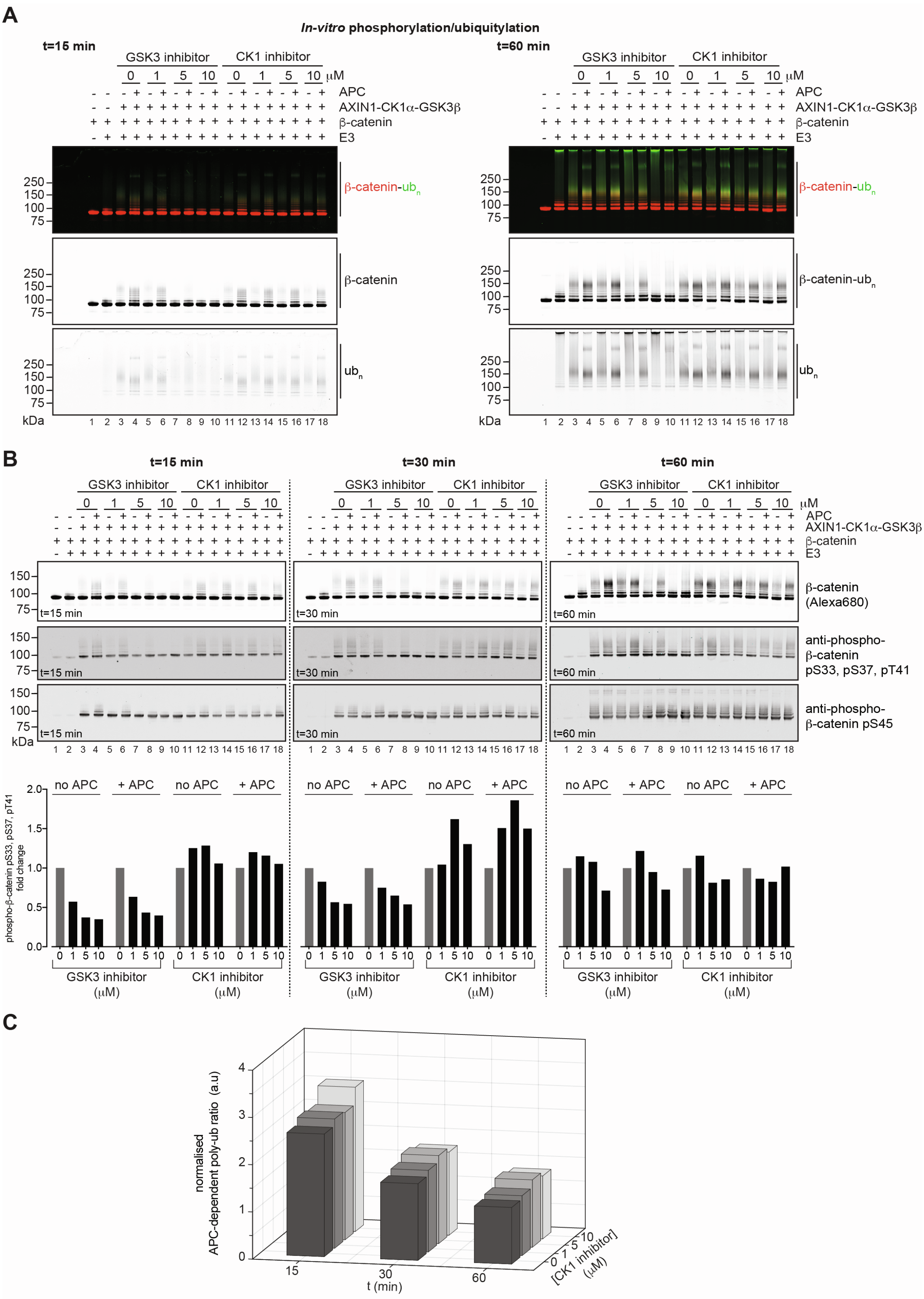

Figure S4. GSK3β inhibition sensitises the *in-vitro* DC phosphorylation/ubiquitylation assay to APC addition. (related to Figure 3)

(**A**) *In-vitro* DC activity assays as in Figure 3F, with different reaction times. Data from the 30-min time point are shown in Figure 3F. (**B**) Analysis of GSK3β- and CK1α-dependent phosphorylation of β-catenin at the indicated reaction time points. The same gels as those shown in A and Figure 3F were used for transfer and immunoblotting. The phospho-β-catenin changes upon inhibitor titration were quantified and expressed relative to those in the absence of kinase inhibitor (set to 1, grey bars) for each condition. (**C**) Normalised APC-dependent β-catenin poly-ubiquitylation, calculated for different reaction time points and different CK1 inhibitor concentrations (n=1 representative experiment).

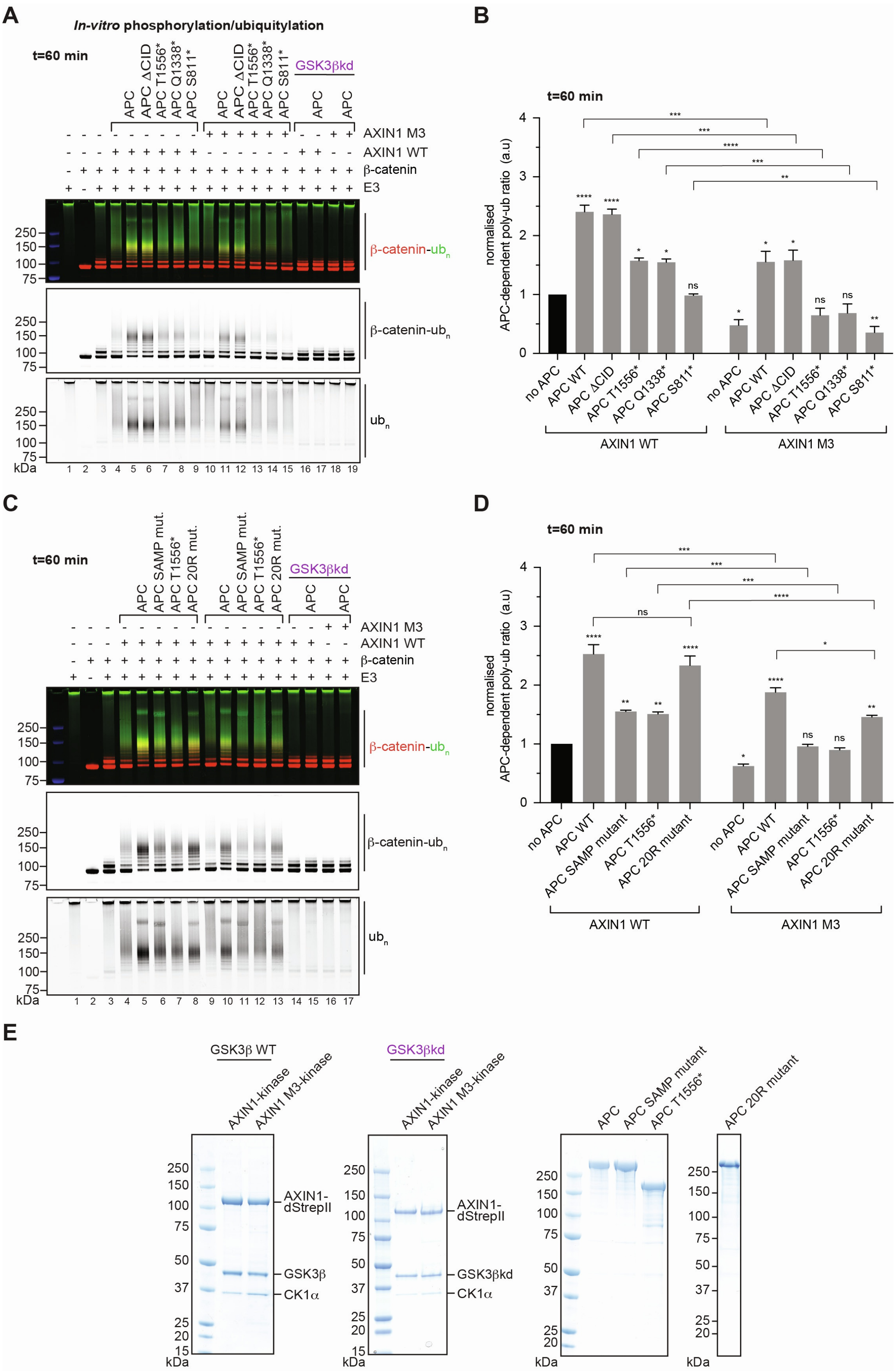

Figure S5. Oncogenic APC truncation and impairment of AXIN1 polymerisation limit DC activity. (related to Figure 4)

(**A** and **C**), *In-vitro* DC activity assay in the presence of 5 μM GSK3 inhibitor. Data are displayed as in Figures 4A and 4C but are from the 60-min reaction time point. (**B** and **D**) Quantification of the APC-dependent poly-ubiquitylation of β-catenin as in Figures 4B and 4D, but from the 60-min reaction time point. Data are means from three independent experiments with error bars representing SEM. Statistical analyses were performed using one-way ANOVA analysis with Bonferroni test for multiple comparisons. Asterisks above histograms (without brackets) refer to comparisons to the no-APC condition (black bar). *$P<0.05$; **$P<0.01$; ***$P<0.001$; ****$P<0.0001$; ns = not significant. See Tables S7 and S9 for details of statistical analyses. (**E**) Coomassie-stained SDS-PAGE gels for purified AXIN1-kinase complexes, wild-type APC, APC SAMP mutant, APC T1556* and APC 20R phospho-site mutant.

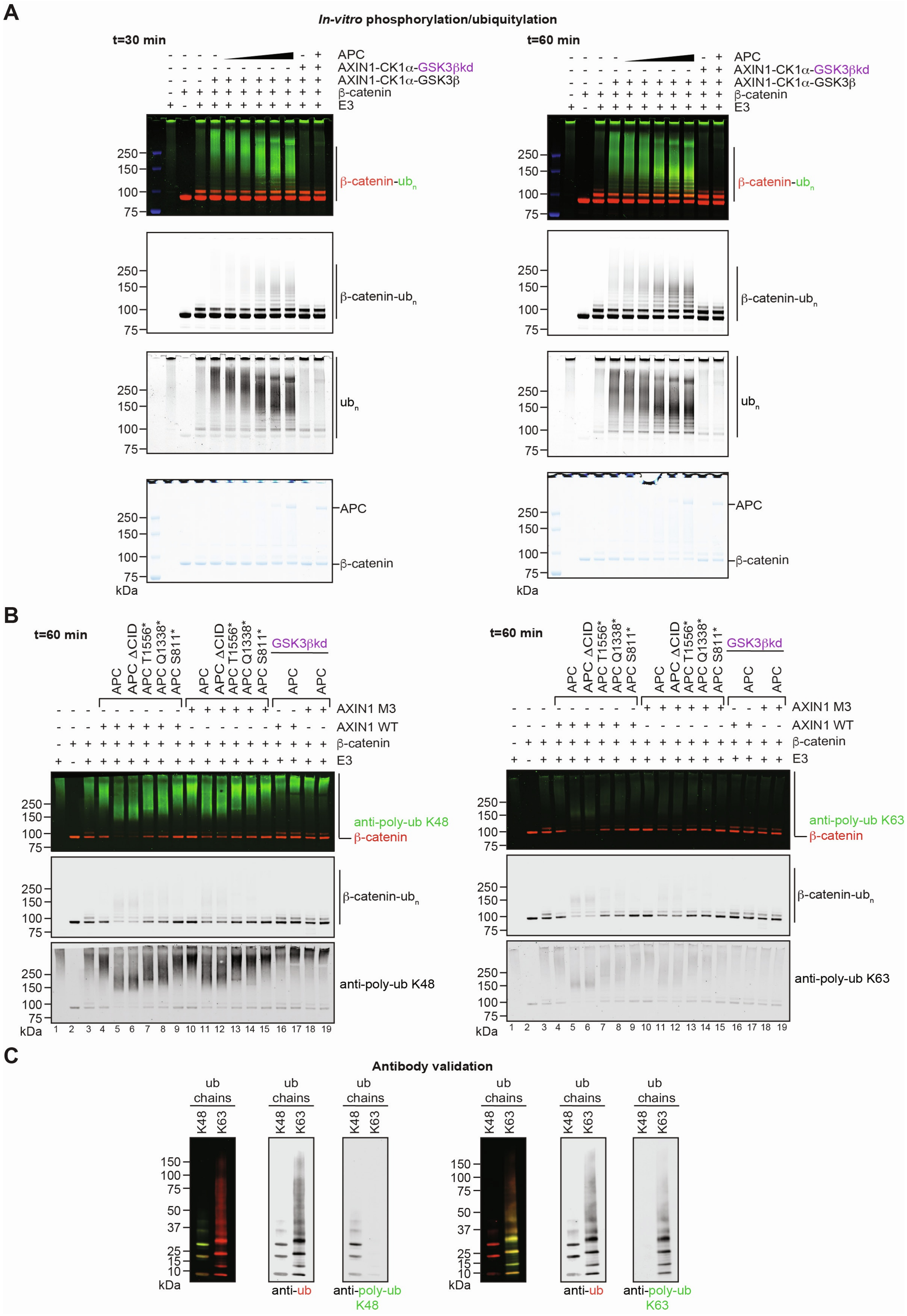

Figure S6. *In-vitro* modification of β-catenin by K48-linked poly-ubiquitin. (related to Figure 4)

(**A**) *In-vitro* DC activity assay in the presence of variable amounts of APC (25, 50, 100, 200, 400 nM) and 5 μM GSK3 inhibitor. (**B**) *In-vitro* DC activity assay in the presence of 10 μM GSK3 inhibitor. Reactions were probed with antibodies against K48-linked (left) or K63-linked poly-ubiquitin (right). (**C**) Validation of the linkage-specific poly-ubiquitin antibodies detecting K48-linked and K63-linked poly-ubiquitin. An anti-ubiquitin antibody was used to detect both chain types.

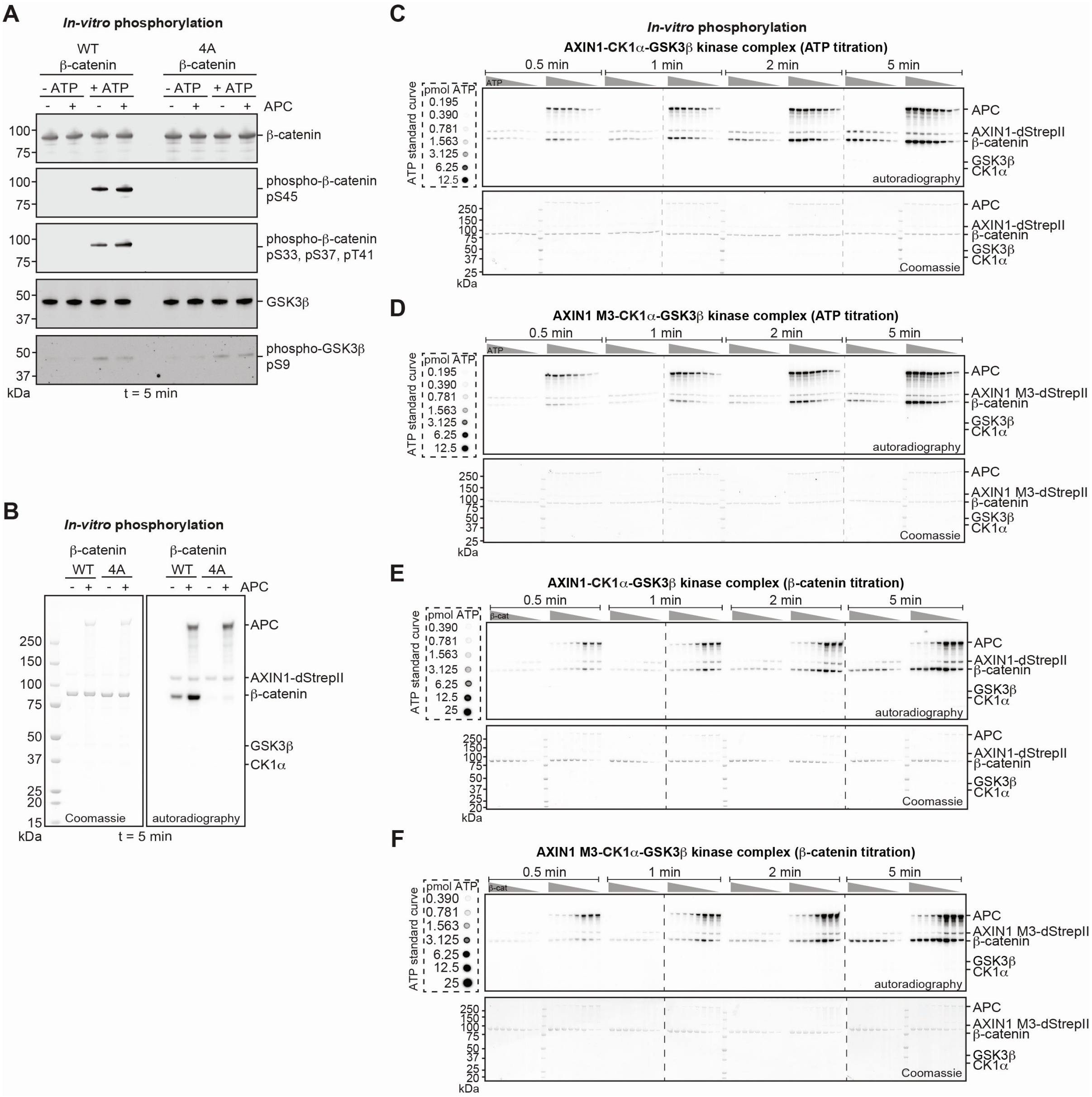

Figure S7. ***In-vitro* β-catenin phosphorylation assays.** (related to Figure 5)

(**A**) Validation of the β-catenin-4A phospho-mutant (S33A, S37A, T41A, S45A) and assessment of GSK3β serine 9 phosphorylation by *in-vitro* phosphorylation and immunoblotting. AXIN1-kinase complex was incubated with the indicated β-catenin variants in the absence and presence of ATP. (**B**) Assessment of overall β-catenin *in-vitro* phosphorylation, not limited to phosphodegron phosphorylation, by incubation with ATP/γ-^{32}P-ATP and autoradiography. APC was added to the reactions as indicated. (**C, D, E, F**) Representative Coomassie-stained gels, their respective autoradiographs and autoradiographs of ATP standard curve serial dilutions for kinetic β-catenin phosphorylation assays. Prior to SDS-PAGE analysis, samples containing >1 μM β-catenin were diluted to 1 μM to prevent gel overloading; the raw values were corrected accordingly during data analysis.

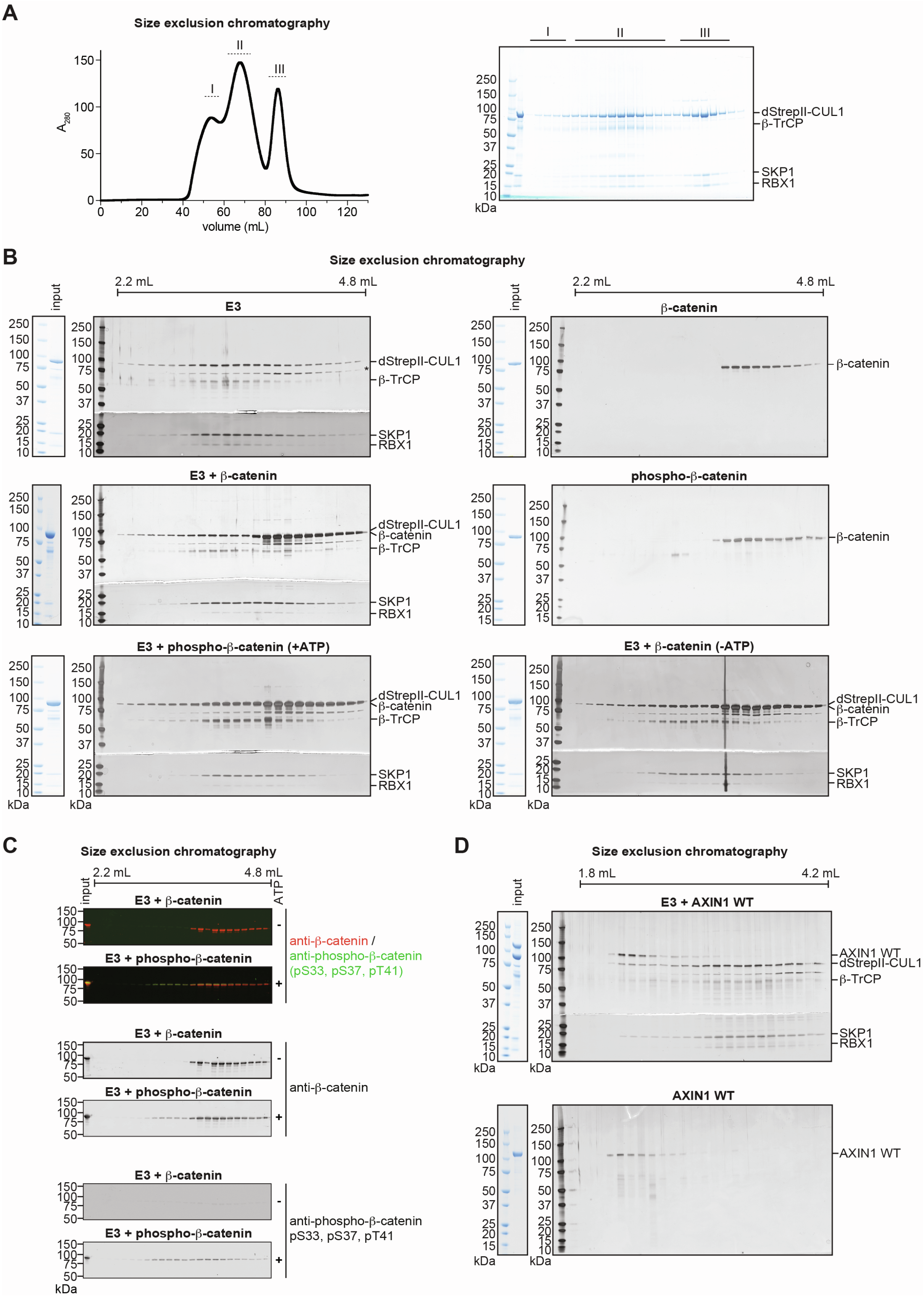

Figure S8. Size exclusion chromatography analysis of SCF$^{\beta\text{-TrCP}}$ with β-catenin or AXIN1. (related to Figure 6)

(**A**) Final (size exclusion chromatography) step in the purification of the SCF$^{\beta\text{-TrCP}}$ complex, with chromatogram (left) and analysis of peak fractions by SDS-PAGE and Coomassie staining (right). Peaks 2 and 3 correspond to the full complex and one lacking β-TrCP, respectively. (**B**) Analytical size exclusion chromatography to study phosphorylation-dependent interactions of the SCF$^{\beta\text{-TrCP}}$ complex with β-catenin. Input samples and SEC fractions were analysed by SDS-PAGE and Coomassie or silver staining, respectively. β-catenin was partially phosphorylated ("phospho-β-catenin") by incubation with sub-stoichiometric amounts of an AXIN1-CK1α-GSK3β complex and ATP. (**C**) Analysis of samples from panel B by immunoblotting, using antibodies recognising either total or GSK3β-phosphorylated β-catenin. (**D**) Analytical size exclusion chromatography to study interaction of the SCF$^{\beta\text{-TrCP}}$ complex with AXIN1. Reference runs with loading of the equivalent fractions are found in Figure 6. Samples were analysed as for panel B.

Table S1. *In-silico* DC stoichiometry modelling corresponding to Figure 1G.

DC AXIN1	Number of molecules					M_n (kDa)	**Maximum stoichiometry** (APC : AXIN1 : kinases : β-catenin)
	APC	AXIN1	CK1α	GSK3β	β-catenin		
**M3**	2	2	1 – 2	1 – 2	4 – 6	1250 – 1507	1 : 1 : 1 : 3
	1	2	1 – 2	1 – 2	3 – 5	853 – 1110	1 : 2 : 2 : 5
	1	3	2 – 3	2 – 3	4 – 5	1124 – 1295	1 : 3 : 3 : 5
**WT**	2	2	1 – 2	1 – 2	12 – 22	1934 – 2875	1 : 1 : 1 : 11
	3	3	2 – 3	2 – 3	13 – 17	2517– 2944	1 : 1 : 1 : 5.7
	3	4	2 – 4	2 – 4	13 – 17	2616 – 2959	1 : 1.3 : 1.3 : 5.7
	2	3	2 – 3	2 – 3	9 – 21	1863 – 2975	1 : 1.5 : 1.5 : 10.5
	3	5	3 – 5	3 – 5	13 – 15	2802 – 2973	1 : 1.7 : 1.7 : 5
	2	4	2 – 4	2 – 4	9 – 20	1963 – 2950	1 : 2 : 2 : 10
	2	5	3 – 5	3 – 5	9 – 18	2148 – 2964	1 : 2.5 : 2.5 : 9
	2	6	3 – 6	3 – 6	9 – 15	2248 – 2971	1 : 3 : 3 : 8.5
	1	3	2 – 3	2 – 3	11 – 13	1722 – 1979	1: 3: 3: 13 (fully saturated unit of the DC)

Table S2. One-way ANOVA analysis corresponding to Figure 2D and STAR Methods (Quantification and statistical analysis).

**Bonferroni's multiple comparisons test**	**Mean diff.**	**95.00% CI of diff.**	**Significant?**	**Summary**	**Adjusted P value**
DC-APC wild-type vs. DC-APC T1556*	0.5053	0.3200 to 0.6905	Yes	****	<0.0001
DC-APC wild-type vs. DC-APC Q1338*	0.4397	0.2544 to 0.6249	Yes	****	<0.0001
DC-APC wild-type vs. DC-APC S811*	0.5649	0.3797 to 0.7502	Yes	****	<0.0001
DC-APC wild-type vs. DC-lacking APC	0.8597	0.6744 to 1.045	Yes	****	<0.0001
DC-APC T1556* vs. DC-APC Q1338*	-0.06559	-0.2508 to 0.1197	No	ns	>0.9999
DC-APC T1556* vs. DC-APC S811*	0.0597	-0.1255 to 0.2449	No	ns	>0.9999
DC-APC T1556* vs. DC-lacking APC	0.3544	0.1692 to 0.5397	Yes	****	<0.0001
DC-APC Q1338* vs. DC-APC S811*	0.1253	-0.05995 to 0.3105	No	ns	0.4774
DC-APC Q1338* vs. DC-lacking APC	0.42	0.2348 to 0.6052	Yes	****	<0.0001
DC-APC S811* vs. DC-lacking APC	0.2947	0.1095 to 0.4800	Yes	***	0.0005

Table S3. One-way ANOVA analysis corresponding to Figure 2E and STAR Methods (Quantification and statistical analysis).

**Bonferroni's multiple comparisons test**	**Mean diff.**	**95.00% CI of diff.**	**Significant?**	**Summary**	**Adjusted P value**
DC-APC wild-type vs. DC-APC T1556*	0.466	0.1120 to 0.8201	Yes	**	0.0082
DC-APC wild-type vs. DC-APC Q1338*	0.3814	0.02740 to 0.7355	Yes	*	0.0317
DC-APC wild-type vs. DC-APC S811*	0.6791	0.3251 to 1.033	Yes	***	0.0004
DC-APC wild-type vs. DC-lacking APC	0.7478	0.3937 to 1.102	Yes	***	0.0002
DC-APC T1556* vs. DC-APC Q1338*	-0.08457	-0.4386 to 0.2695	No	ns	>0.9999
DC-APC T1556* vs. DC-APC S811*	0.2131	-0.1410 to 0.5671	No	ns	0.5653
DC-APC T1556* vs. DC-lacking APC	0.2817	-0.07231 to 0.6358	No	ns	0.1725
DC-APC Q1338* vs. DC-APC S811*	0.2977	-0.05639 to 0.6517	No	ns	0.1309
DC-APC Q1338* vs. DC-lacking APC	0.3663	0.01226 to 0.7203	Yes	*	0.0407
DC-APC S811* vs. DC-lacking APC	0.06864	-0.2854 to 0.4227	No	ns	>0.9999

Table S4. One-way ANOVA analysis corresponding to Figure 2F and STAR Methods (Quantification and statistical analysis).

**Bonferroni's multiple comparisons test**	**Mean diff.**	**95.00% CI of diff.**	**Significant?**	**Summary**	**Adjusted P value**
DC-APC wild-type vs. DC-APC T1556*	0.4528	0.08416 to 0.8214	Yes	*	0.0134
DC-APC wild-type vs. DC-APC Q1338*	0.3932	0.02454 to 0.7618	Yes	*	0.0337
DC-APC wild-type vs. DC-APC S811*	0.6682	0.2996 to 1.037	Yes	***	0.0007
DC-APC wild-type vs. DC-lacking APC	0.7858	0.4171 to 1.154	Yes	***	0.0002
DC-APC T1556* vs. DC-APC Q1338*	-0.05962	-0.4282 to 0.3090	No	ns	>0.9999
DC-APC T1556* vs. DC-APC S811*	0.2154	-0.1532 to 0.5840	No	ns	0.6283
DC-APC T1556* vs. DC-lacking APC	0.333	-0.03563 to 0.7016	No	ns	0.0894
DC-APC Q1338* vs. DC-APC S811*	0.275	-0.09359 to 0.6436	No	ns	0.2341
DC-APC Q1338* vs. DC-lacking APC	0.3926	0.02399 to 0.7612	Yes	*	0.034
DC-APC S811* vs. DC-lacking APC	0.1176	-0.2510 to 0.4862	No	ns	>0.9999

Table S5. One-way ANOVA analysis corresponding to Figure 3E and STAR Methods (Quantification and statistical analysis).

**Bonferroni's multiple comparisons test**	**Mean diff.**	**95.00% CI of diff.**	**Significant?**	**Summary**	**Adjusted P value**
no APC vs. APC WT	-0.1165	-0.1709 to -0.06203	Yes	****	<0.0001
no APC vs. APC ΔCID	-0.09181	-0.1463 to -0.03737	Yes	***	0.0009
no APC vs. APC T1556*	-0.04927	-0.1037 to 0.005174	No	ns	0.0906
no APC vs. APC Q1338*	-0.04389	-0.09834 to 0.01055	No	ns	0.1681
no APC vs. APC S811*	0.007780	-0.04666 to 0.06222	No	ns	>0.9999
APC WT vs. APC ΔCID	0.02466	-0.02978 to 0.07910	No	ns	>0.9999
APC WT vs. APC T1556*	0.06721	0.01276 to 0.1216	Yes	*	0.0119
APC WT vs. APC Q1338*	0.07258	0.01814 to 0.1270	Yes	**	0.0066
APC WT vs. APC S811*	0.1243	0.06981 to 0.1787	Yes	****	<0.0001

Table S6. One-way ANOVA analysis corresponding to Figure 4B and STAR Methods (Quantification and statistical analysis).

**Bonferroni's multiple comparisons test**	**Mean diff.**	**95.00% CI of diff.**	**Significant?**	**Summary**	**Adjusted P value**
no APC vs. APC WT	-5.037	-6.880 to -3.194	Yes	****	<0.0001
no APC vs. APC ΔCID	-5.158	-7.001 to -3.315	Yes	****	<0.0001
no APC vs. APC T1556*	-1.855	-3.698 to -0.01211	Yes	*	0.0474
no APC vs. APC Q1338*	-1.847	-3.690 to -0.004193	Yes	*	0.0491
no APC vs. APC S811*	-0.06197	-1.905 to 1.781	No	ns	>0.9999
no APC vs. no APC_AXIN M3	0.5509	-1.292 to 2.394	No	ns	>0.9999
no APC vs. APC WT_AXIN M3	-1.500	-3.342 to 0.3434	No	ns	0.2129
no APC vs. APC ΔCID_AXIN M3	0.4440	-1.399 to 2.287	No	ns	>0.9999
no APC vs. APC T1556*_AXIN M3	0.3777	-1.465 to 2.221	No	ns	>0.9999
no APC vs. APC Q1338*_AXIN M3	0.7412	-1.102 to 2.584	No	ns	>0.9999
no APC vs. APC S811*_AXIN M3	-1.907	-3.750 to -0.06458	Yes	*	0.0377
APC WT vs. APC WT_AXIN M3	3.538	1.695 to 5.380	Yes	****	<0.0001
APC ΔCID vs. APC ΔCID_AXIN M3	3.250	1.408 to 5.093	Yes	****	<0.0001
APC T1556* vs. APC T1556*_AXIN M3	2.299	0.4561 to 4.142	Yes	**	0.0066
APC Q1338* vs. APC Q1338*_AXIN M3	2.225	0.3819 to 4.068	Yes	**	0.0092
APC S811* vs. APC S811*_AXIN M3	0.8031	-1.040 to 2.646	No	ns	>0.9999

Table S7. One-way ANOVA analysis corresponding to Figure S5B, which is related to Figure 4, and STAR Methods (Quantification and statistical analysis).

Bonferroni's multiple comparisons test	Mean diff.	95.00% CI of diff.	Significant?	Summary	Adjusted P value
no APC vs. APC WT	-1.406	-1.925 to -0.8878	Yes	****	<0.0001
no APC vs. APC ΔCID	-1.362	-1.881 to -0.8434	Yes	****	<0.0001
no APC vs. APC T1556*	-0.5749	-1.093 to -0.05630	Yes	*	0.0207
no APC vs. APC Q1338*	-0.5479	-1.066 to -0.02928	Yes	*	0.0317
no APC vs. APC S811*	0.01609	-0.5025 to 0.5347	No	ns	>0.9999
no APC vs. no APC_AXIN M3	0.5210	0.002445 to 1.040	Yes	*	0.0481
no APC vs. APC WT_AXIN M3	-0.5538	-1.072 to -0.03516	Yes	*	0.0289
no APC vs. APC ΔCID_AXIN M3	-0.5814	-1.100 to -0.06279	Yes	*	0.0187
no APC vs. APC T1556*_AXIN M3	0.3520	-0.1666 to 0.8706	No	ns	0.5659
no APC vs. APC Q1338*_AXIN M3	0.3142	-0.2044 to 0.8328	No	ns	0.9293
no APC vs. APC S811*_AXIN M3	0.6456	0.1270 to 1.164	Yes	**	0.0067
APC WT vs. APC WT_AXIN M3	0.8526	0.3340 to 1.371	Yes	***	0.0002
APC ΔCID vs. APC ΔCID_AXIN M3	0.7806	0.2620 to 1.299	Yes	***	0.0008
APC T1556* vs. APC T1556*_AXIN M3	0.9269	0.4083 to 1.446	Yes	****	<0.0001
APC Q1338* vs. APC Q1338*_AXIN M3	0.8621	0.3435 to 1.381	Yes	***	0.0002
APC S811* vs. APC S811*_AXIN M3	0.6295	0.1109 to 1.148	Yes	**	0.0087

Table S8. One-way ANOVA analysis corresponding to Figure 4D and STAR Methods (Quantification and statistical analysis).

Bonferroni's multiple comparisons test	Mean diff.	95.00% CI of diff.	Significant?	Summary	Adjusted P value
no APC vs. APC WT	-5.506	-6.817 to -4.195	Yes	****	<0.0001
no APC vs. APC SAMP mut.	-1.993	-3.304 to -0.6820	Yes	***	0.0009
no APC vs. APC T1556*	-1.944	-3.255 to -0.6332	Yes	**	0.0012
no APC vs. APC 20R mut.	-4.255	-5.566 to -2.944	Yes	****	<0.0001
no APC vs. no APC_AXIN M3	0.4914	-0.8195 to 1.802	No	ns	>0.9999
no APC vs. APC WT_AXIN M3	-2.477	-3.788 to -1.166	Yes	****	<0.0001
no APC vs. APC SAMP mut. AXIN_M3	0.09441	-1.217 to 1.405	No	ns	>0.9999
no APC vs. APC T1556*_AXIN M3	0.2288	-1.082 to 1.540	No	ns	>0.9999
no APC vs. APC 20R mut._AXIN M3	-1.044	-2.355 to 0.2667	No	ns	0.2290
APC WT vs. APC WT_AXIN M3	3.028	1.717 to 4.339	Yes	****	<0.0001
APC SAMP mut. Vs. APC SAMP mut. Axin_M3	2.087	0.7764 to 3.398	Yes	***	0.0005
APC T1556* vs. APC T1556*_AXIN M3	2.173	0.8620 to 3.484	Yes	***	0.0003
APC 20R mut. Vs. APC 20R mut._AXIN M3	3.211	1.900 to 4.522	Yes	****	<0.0001
APC WT vs. APC 20R mut.	1.251	-0.06032 to 2.562	No	ns	0.0710
APC WT_M3 vs. APC 20R mut._AXIN M3	1.433	0.1223 to 2.744	Yes	*	0.0244

Table S9. One-way ANOVA analysis corresponding to Figure S5D, which is related to Figure 4, and STAR Methods (Quantification and statistical analysis).

Bonferroni's multiple comparisons test	Mean diff.	95.00% CI of diff.	Significant?	Summary	Adjusted P value
no APC vs. APC WT	-1.528	-1.900 to -1.156	Yes	****	<0.0001
no APC vs. APC SAMP mut.	-0.5513	-0.9235 to -0.1791	Yes	**	0.0012
no APC vs. APC T1556*	-0.5072	-0.8794 to -0.1350	Yes	**	0.0030
no APC vs. APC 20R mut.	-1.337	-1.709 to -0.9644	Yes	****	<0.0001
no APC vs. no APC_AXIN M3	0.3765	0.004260 to 0.7487	Yes	*	0.0458
no APC vs. APC WT_AXIN M3	-0.8799	-1.252 to -0.5077	Yes	****	<0.0001
no APC vs. APC SAMP mut. AXIN_M3	0.04048	-0.3317 to 0.4127	No	ns	>0.9999
no APC vs. APC T1556*_AXIN M3	0.1024	-0.2698 to 0.4746	No	ns	>0.9999
no APC vs. APC 20R mut._AXIN M3	-0.4596	-0.8318 to -0.08735	Yes	**	0.0081
APC WT vs. APC WT_AXIN M3	0.1911	-0.1811 to 0.5633	No	ns	>0.9999
APC SAMP mut. vs. APC SAMP mut. Axin_M3	0.4204	0.04817 to 0.7926	Yes	*	0.0184
APC T1556* vs. APC T1556*_AXIN M3	0.6478	0.2756 to 1.020	Yes	***	0.0002
APC 20R mut. vs. APC 20R mut._AXIN M3	0.5918	0.2196 to 0.9640	Yes	***	0.0005
APC WT vs. APC 20R mut.	0.6096	0.2374 to 0.9818	Yes	***	0.0004
APC WT_M3 vs. APC 20R mut._AXIN M3	0.8771	0.5049 to 1.249	Yes	****	<0.0001

Table S10. Individual expression constructs for DC subunits. UniProt IDs (UniProt Consortium, 2021) are provided. (related to Figure 1B, 2A-B, 3A-C, S5E, S7A-B)

insert in pLIB vector	UniProt ID	tag	mutations
APC	P25054 APC_ HUMAN	N-term	
APC T1156*		N-term	Based on COLO-205 cell line, APC p.T1556Nfs*3 mutation, which replaces the T1556 codon with codons for N, Y and stop (*).
APC Q1338*		N-term	Based on SW480 cell line, harbouring a nonsense point mutation at the Q1338 codon.
APC S811*		N-term	Based on COLO320-DM cell line, harbouring a nonsense point mutation at the S811 codon.
APC ΔCID		N-term	S1404 – G1466 deletion
APC SAMP mutant		N-term	SAMP1: I1579A, M1583A, P1584A<br>SAMP2: I1728A, M1732A, P1733A<br>SAMP3: I2043A, M2047A, P2048A<br><br>Mutations were designed based on the crystal structure of the human AXIN1 RGS domain bound to the human APC SAMP3 repeat (Spink et al., 2000; PDB: 1EMU).
APC 20R phospho mutant		N-term	20R1: S1272A, S1275A, S1276A, S1278A, S1279A, S1281A<br>20R2: S1385A, T1388A, S1389A, S1391A, S1392A<br>20R3: S1501A, S1504A, S1505A, S1507A, S1510A<br>20R4: S1652A, T1655A, S1656A, S1658A, T1661A<br>20R5: S1857A, S1861A, S1863A, S1864A<br>20R6: S1967A, S1968A, S1970A, S1971A, S1973A<br>20R7: S2022A, S2025A, S2026A, S2028A, S2029A, S2031A<br><br>Mutations were designed based on Ha et al., 2004, which reports the crystal structure of phosphorylated 20R3 from human APC bound to the armadillo repeat region of murine β-catenin (PDB: 1V18).
AXIN1	O15169 AXIN1_ HUMAN	C-term	
AXIN1 M3		C-term	I794A, R797D (Fiedler et al., 2011)
β-catenin	P35222 CTNB1_ HUMAN	N-term	
β-catenin-4A phospho-mutant		N-term	S33A, S37A, T41A, S45A
β-catenin -ybbR		N-term	C-terminal ybbR; *-linker (GGGS)* – DSLEFIASKLA * stop
GSK3β	P49841 GSK3B_ HUMAN		
GSK3β kinase-dead			K85A, D181N (PDB: 1H8F; Dajani et al., 2001)
CK1α	P48729 KC1A_ HUMAN		
β-TrCP	Q9UKB1 FBW1B_ HUMAN		
CUL1	Q13616 CUL1_ HUMAN	N-term	
SKP1	P63208		

	SKP1_ HUMAN		
RBX1	P62877 RBX1_ HUMAN		

Table S11. Purified recombinant protein complexes. (related to Figure 1B, 2A-B, 3A-C, S5E)

Construct	Description
AXIN1-kinase complex	pBIG1a vector containing: AXIN1-TEV-dStrepII, CK1α, GSK3β
AXIN1-kinase GSK3β-dead complex	pBIG1a vector containing: AXIN1-TEV-dStrepII, CK1α, GSK3β K85A, D181N
AXIN1 M3-kinase complex	pBIG1a vector containing: AXIN1 M3-TEV-dStrepII, CK1α, GSK3β
AXIN1 M3-kinase GSK3β-dead complex	pBIG1a vector containing: AXIN1 M3-TEV-dStrepII, CK1α, GSK3β K85A, D181N
AXIN1-kinase β-catenin complex	pBIG1a vector containing: AXIN1-TEV-dStrepII, β-catenin, CK1α, GSK3β
wild-type destruction complex (DC)	pBIG2ab vector containing: APC, AXIN1-TEV-dStrepII, β-catenin, CK1α, GSK3β
DC – AXIN1 M3	pBIG2ab vector containing: APC, AXIN1 M3-TEV-dStrepII, β-catenin, CK1α, GSK3β
DC – APC T1556*	pBIG2ab vector containing: APC T1556*, AXIN1-TEV-dStrepII, β-catenin, CK1α, GSK3β
DC – APC Q1338*	pBIG2ab vector containing: APC Q1338*, AXIN1-TEV-dStrepII, β-catenin, CK1α, GSK3β
DC – APC S811*	pBIG2ab vector containing: APC S811*, AXIN1-TEV-dStrepII, β-catenin, CK1α, GSK3β
$SCF^{β\text{-}TrCP}$ E3 ubiquitin ligase	pBIG2ab vector containing: β-TrCP, dStrepII-TEV-CUL1, SKP1, RBX1
