# Supplementary material for: Reconstitution of the destruction complex defines roles of AXIN polymers and APC in β-catenin capture, phosphorylation, and ubiquitylation
Source: Mol Cell. 2021 Aug 19;81(16):3246–3261.e11. doi: 10.1016/j.molcel.2021.07.013 (PMC8403986; doi:10.1016/j.molcel.2021.07.013)
Supplement: Document S2. Article plus supplemental information [file mmc3.pdf]

Molecular Cell

Article

Reconstitution of the destruction complex defines roles of AXIN polymers and APC in β-catenin capture, phosphorylation, and ubiquitylation

Graphical abstract

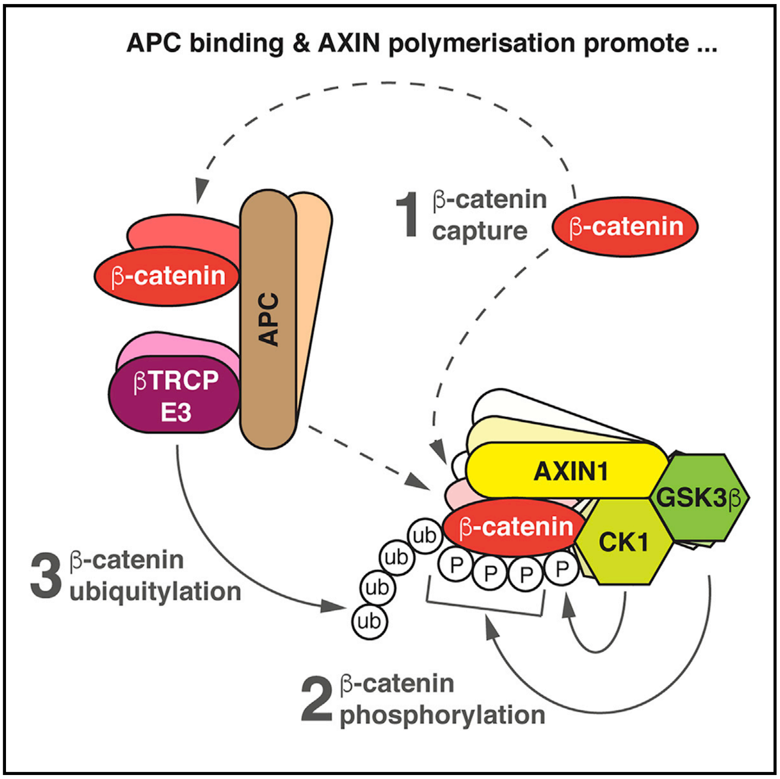

Highlights

- β-Catenin destruction complex function recapitulated with purified proteins
- AXIN1 polymers and APC promote β-catenin capture, phosphorylation, and ubiquitylation
- Oncogenic APC truncation mutants are hypomorphs promoting β-catenin recruitment
- APC directly binds the ubiquitylation machinery

Authors

Michael Ranes, Mariola Zaleska, Saira Sakalas, Ruth Knight, Sebastian Guettler

Correspondence

sebastian.guettler@icr.ac.uk

In brief

Ranes et al. reconstitute the β-catenin destruction complex from purified proteins, thereby recapitulating its assembly and biochemical activities and enabling the mechanistic interrogation of its scaffolding proteins AXIN1 and APC. AXIN1 polymerization and APC facilitate the recruitment, phosphorylation, and ubiquitylation of β-catenin. Oncogenic truncation of APC partially preserves APC function.

Ranes et al., 2021, Molecular Cell *81*, 3246–3261
August 19, 2021 © 2021 The Author(s). Published by Elsevier Inc.
https://doi.org/10.1016/j.molcel.2021.07.013

CellPress

CellPress
OPEN ACCESS

Molecular Cell

Article

Reconstitution of the destruction complex defines roles of AXIN polymers and APC in β-catenin capture, phosphorylation, and ubiquitylation

Michael Ranes,[1,2] Mariola Zaleska,[1,2] Saira Sakalas,[1,2] Ruth Knight,[1] and Sebastian Guettler[1,2,3,*]
[1]Division of Structural Biology, The Institute of Cancer Research (ICR), London, UK
[2]Division of Cancer Biology, The Institute of Cancer Research (ICR), London, UK
[3]Lead contact
*Correspondence: sebastian.guettler@icr.ac.uk
https://doi.org/10.1016/j.molcel.2021.07.013

SUMMARY

The Wnt/β-catenin pathway is a highly conserved, frequently mutated developmental and cancer pathway. Its output is defined mainly by β-catenin's phosphorylation- and ubiquitylation-dependent proteasomal degradation, initiated by the multi-protein β-catenin destruction complex. The precise mechanisms underlying destruction complex function have remained unknown, largely because of the lack of suitable *in vitro* systems. Here we describe the *in vitro* reconstitution of an active human β-catenin destruction complex from purified components, recapitulating complex assembly, β-catenin modification, and degradation. We reveal that AXIN1 polymerization and APC promote β-catenin capture, phosphorylation, and ubiquitylation. APC facilitates β-catenin's flux through the complex by limiting ubiquitylation processivity and directly interacts with the $SCF^{β\text{-}TrCP}$ E3 ligase complex in a β-TrCP-dependent manner. Oncogenic APC truncation variants, although part of the complex, are functionally impaired. Nonetheless, even the most severely truncated APC variant promotes β-catenin recruitment. These findings exemplify the power of biochemical reconstitution to interrogate the molecular mechanisms of Wnt/β-catenin signaling.

INTRODUCTION

The highly conserved Wnt/β-catenin signaling pathway coordinates key events in early embryogenesis, tissue homeostasis, and regeneration, governing stem cell maintenance, cell fate specification, and cell proliferation (Clevers et al., 2014; Steinhart and Angers, 2018). It is one of the most frequently mutated pathways in cancer (Sanchez-Vega et al., 2018). The degree of its activation is the outcome of balanced activities of two opposing multi-protein complexes that determine the fate of newly synthesized cytoplasmic β-catenin: the β-catenin destruction complex (DC) and the Wnt signalosome (Gammons and Bienz, 2018; Stamos and Weis, 2013; van Kappel and Maurice, 2017). The DC predominates at basal Wnt/β-catenin signaling and, via phosphorylation-dependent ubiquitylation, earmarks β-catenin for proteasomal degradation. Receptor engagement by Wnt growth factors converts the DC into a receptor-associated Wnt signalosome complex, where β-catenin phosphorylation and ubiquitylation are attenuated, resulting in increased levels and nuclear accumulation of β-catenin and the expression of β-catenin/T cell factor/lymphoid enhancer-binding factor (TCF/LEF) target genes (Gammons and Bienz, 2018).

In the DC, the scaffolding proteins and tumor suppressors axis inhibition protein 1 (AXIN1) and adenomatous polyposis coli (APC) collaborate to co-recruit the kinases casein kinase 1 (CK1) and glycogen synthase kinase 3β (GSK3β) jointly with their substrate β-catenin (Stamos and Weis, 2013; van Kappel and Maurice, 2017; Figure 1A). This enables the sequential phosphorylation of an N-terminal β-catenin phosphodegron and the subsequent ubiquitylation of newly synthesized β-catenin by a SKP1-CUL1-F box (SCF) E3 ubiquitin ligase complex containing the substrate recruitment component β-TrCP ($SCF^{β\text{-}TrCP}$) (Amit et al., 2002; Jiang and Struhl, 1998; Li et al., 2012; Liu et al., 2002; Orford et al., 1997; Stamos and Weis, 2013; van Kappel and Maurice, 2017; Winston et al., 1999; Wu et al., 2003). Beta-catenin poly-ubiquitylation triggers its proteasomal degradation (Aberle et al., 1997).

The central DC scaffold, AXIN1, directly binds and assembles all components of the core DC, strongly boosting β-catenin phosphorylation (Dajani et al., 2003; Hamada et al., 1999; Ikeda et al., 1998; Willert et al., 1999), in part by suppressing phosphorylation of substrates competing with β-catenin (Gavagan et al., 2020). Its low average cellular concentration (Lee et al., 2003; Tan et al., 2012) requires AXIN1 to form filamentous polymers for efficient β-catenin degradation (Fiedler et al., 2011). The second DC scaffold, APC, harbors up to ten β-catenin binding sites, a subset of which are phospho-regulated by the DC kinases (Eklof Spink et al., 2001; Ha et al., 2004; Liu et al., 2006;

 © 2021 The Author(s). Published by Elsevier Inc.
This is an open access article under the CC BY license (http://creativecommons.org/licenses/by/4.0/).

CellPress OPEN ACCESS

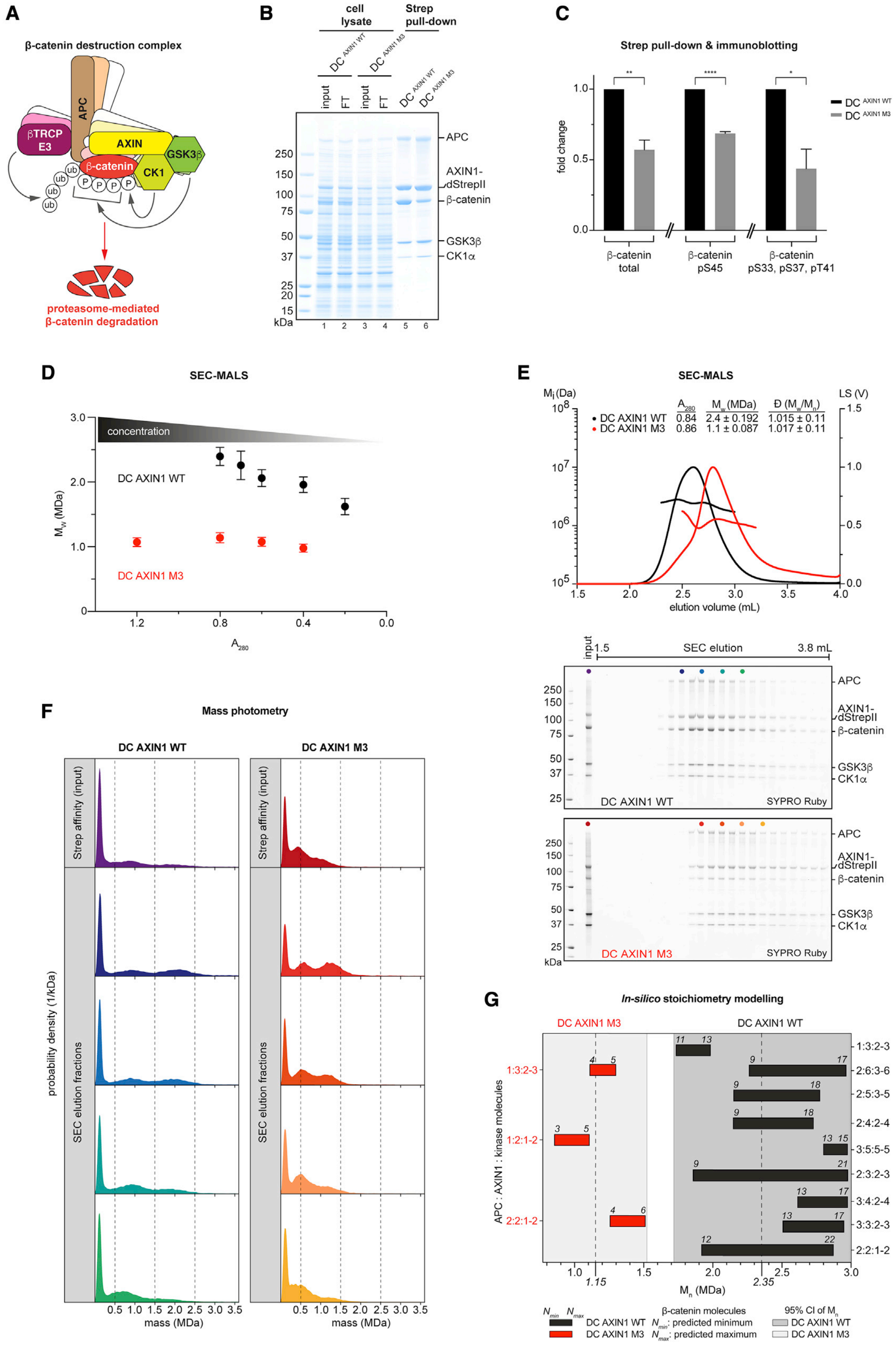

(legend on next page)

Rubinfeld et al., 1993; Su et al., 1993), as well as three AXIN1 docking motifs (Behrens et al., 1998; Kishida et al., 1998; Spink et al., 2000; Stamos and Weis, 2013; van Kappel and Maurice, 2017; Figure S1A). Loss of varying numbers of these motifs by APC truncating mutations within the intestinal stem cell compartment confers high basal β-catenin levels and initiates up to 80% of colorectal cancers (CRCs) (Barker and Clevers, 2006; Kohler et al., 2008, 2009; Morin et al., 1997; Munemitsu et al., 1995; Novellasdemunt et al., 2017; Roberts et al., 2011; Rubinfeld et al., 1997; Zhang and Shay, 2017), the third most common cancer type worldwide (Bray et al., 2018). Although the function of AXIN1 in the DC is relatively well understood, that of APC is less clear (van Kappel and Maurice, 2017). Several non-mutually exclusive functions have been proposed for APC, among them the capture of β-catenin in the cytoplasm for its modification (Ha et al., 2004) or cytoplasmic retention (Krieghoff et al., 2006; Roberts et al., 2011), the transfer of phosphorylated β-catenin from AXIN1 to the ubiquitin-proteasome system (Kimelman and Xu, 2006), or the protection of β-catenin from de-phosphorylation (Su et al., 2008) or de-ubiquitylation (Novellasdemunt et al., 2017).

Our understanding of the DC is shaped largely by genetic, cell biological, cellular biochemical, and structural studies (Stamos and Weis, 2013; van Kappel and Maurice, 2017; and references above). A minimal DC has been constructed in mammalian cells (Pronobis et al., 2017), and the reconstitution of DC function in mammalian cell (Su et al., 2008) or *Xenopus* egg (Salic et al., 2000) extracts has been the closest step toward achieving full DC reconstitution *in vitro*. Still, both these systems offer limited control over the factors present in the experimental setup. The biochemical reconstitution of the DC from purified components, thus far hampered by the lack of purified, recombinant, full-length human AXIN1 and APC, has long been sought after, as it would enable the controlled interrogation of DC function in a reductionist system without confounding cellular factors.

Here we describe the *in vitro* reconstitution of the core DC and recapitulate β-catenin capture, phosphorylation, ubiquitylation, and degradation, using purified components. We demonstrate that oncogenic APC mutations in CRC impart deficiencies to DC assembly and activities.

RESULTS

Biochemical reconstitution of the DC

To interrogate the mechanism of the core DC, we expressed the recombinant human complex in insect cells and affinity-purified it through a C-terminal double-StrepII tag on AXIN1. We included AXIN1 either in its wild-type or non-polymerizable mutant form (M3) (Fiedler et al., 2011). All full-length DC components (AXIN1, APC, CK1α, GSK3β, and β-catenin) were present in the purified complex (Figure 1B). However, loss of AXIN1 polymerization significantly reduced β-catenin recruitment (Figures 1B, 1C, and S1B), in agreement with the essential role of AXIN1 self-assembly in destabilizing β-catenin *in vivo* (Fiedler et al., 2011). Phosphorylation of β-catenin, APC, and AXIN1 is critical to the DC function (Stamos and Weis, 2013; van Kappel and Maurice, 2017). Immunoblotting revealed that a subpopulation of β-catenin within the complex was phosphorylated on the authentic phosphodegron sites targeted by CK1α and GSK3β (S45 and S33/S37/T41, respectively; Figure S1B; Liu et al., 2002). Loss of AXIN1 polymerization also significantly reduced degron phosphorylation (Figures 1C and S1B). Treatment of the purified DC with λ-phosphatase increased APC and AXIN1 mobility in SDS-PAGE and abolished detectable β-catenin phosphorylation (Figures S1C). Conversely, ATP addition reduced AXIN1 mobility and further augmented β-catenin phosphorylation, showing that phosphorylation of these components was not saturated (Figure S1C).

We next analyzed the DC's mass by size exclusion chromatography coupled to in-line multi-angle light scattering (SEC-MALS). AXIN1 (92 kDa) is expected to bind a single copy each of β-catenin (85 kDa), CK1α (39 kDa), and GSK3β (47 kDa). Disregarding AXIN1 and APC multimerization (Fiedler et al., 2011; Kunttas-Tatli et al., 2014), we reasoned that up to three AXIN1 molecules could be bound by a single APC molecule (312 kDa) through APC's SAMP repeats. APC would bind up to ten β-catenin molecules through its 15Rs and 20Rs. The resulting overall theoretical molecular weight would amount to 1,951 kDa (Figure S1A). At its highest attainable concentration, the DC displayed an average molecular weight of 2,395 ± 141 kDa (Figures 1D and S1D–S1F), indicating additional higher order contributions to DC stoichiometry. Dilution of the complex decreased

Figure 1. AXIN1 polymerization is a major contributor to the concentration-dependent DC stoichiometry

(A) Schematic representation of the DC. See Figure S1A for further details.

(B) DC variants containing either wild-type or polymerization-deficient (M3) AXIN1, affinity-purified via AXIN1-dStrepII, and analyzed using SDS-PAGE and Coomassie staining. FT, flowthrough.

(C) Quantification of total β-catenin recovered with AXIN1-dStrepII and its phosphorylation, normalized to recovered total β-catenin. Data are from immunoblot analyses of experiments as shown in (B). See Figure S1B for a representative set of immunoblots. Results are means from three independent experiments ± SEM. Statistical analyses were performed using an unpaired Student's t test. *$p < 0.05$, **$p < 0.01$, ***$p < 0.001$, and ****$p < 0.0001$.

(D) SEC-MALS of the DC containing either wild-type or M3 AXIN1 at different input concentrations. Data points are mean weight average molecular weights (M_W) ± uncertainty (see STAR Methods). See Figure S1D for SDS-PAGE analysis of input samples and Figures S1E and S1F for SEC-MALS data and representative SDS-PAGE analyses of elution fractions.

(E) SEC-MALS of the DC containing either wild-type or M3 AXIN1 at a single input concentration. Light scattering intensities at 90° and calculated M_i values are plotted. Input A_{280}, M_W, and dispersities (Đ) over the peak areas with uncertainties are tabulated. Peak fractions were analyzed using SDS-PAGE and Sypro Ruby staining.

(F) Mass photometry of input and SEC-MALS elution fractions (indicated by colored dots in E).

(G) *In silico* modeling of DC stoichiometry. Predicted stoichiometries are shown on the right- or left-hand y axes for the DC with wild-type or M3 AXIN1, respectively. Dashed lines indicate mean number average molecular weight (M_n) values from SEC-MALS; shaded areas indicate corresponding 95% confidence intervals.

CellPress
OPEN ACCESS

the measured molecular weights, in line with the anticipated concentration dependency of complex assembly (Figures 1D and S1D–S1F). The size of the DC was not saturated at its highest concentration, but we were unable to concentrate the complex by ultrafiltration. AXIN1 polymerization is a major contributor to the overall size of the DC: even at a higher concentration than that for the wild-type DC, the AXIN1 M3 mutant complex displayed an average molecular weight of only 1,072 ± 68 kDa, and its dilution had no impact on its overall mass (Figures 1D and S1D–S1F). As for affinity purification, the eluting complex showed an increased β-catenin occupancy with wild-type compared with M3 AXIN1 (Figures S1E and S1F).

We next used mass photometry to measure the DC's mass distribution at the single-molecule level (Sonn-Segev et al., 2020; Young et al., 2018). We first performed SEC-MALS on DCs containing either wild-type or M3 AXIN1 (Figure 1E), with results comparable with those shown in Figure 1D. We next analyzed the SEC-MALS input material and fractions across the elution peaks (Figure 1E, indicated by colored dots on gels) using mass photometry (Figure 1F). Molecular weight distributions were broad. The dominance of events below 0.2 MDa indicated DC dissociation upon dilution for mass photometry (<100 nM) (Sonn-Segev et al., 2020), even within the short time frame (≈5 min). This generated multiple lower molecular weight events from the parental complex, in line with the dynamic, concentration-dependent assembly of the DC (Barua and Hlavacek, 2013; Nong et al., 2021; Pronobis et al., 2017; Schaefer and Peifer, 2019). Nonetheless, we still detected high-molecular weight species (Figure 1F). Although most events measured for DC AXIN1 M3 were less than 1.5 MDa, the wild-type DC displayed a broader mass distribution ranging up to ≈3 MDa (Figure 1F). In conclusion, mass photometry further illustrates the importance of AXIN1 polymerization in DC assembly.

We performed *in silico* modeling to predict possible stoichiometries within the DC that satisfy both the molecular weights observed in SEC-MALS and constraints on the basis of our own and published findings on DC assembly (Figure 1G; Table S1; see STAR Methods for details). Modeling suggests that the DC can attain different stoichiometries for a given molecular weight range. Although AXIN1 polymerization and the APC/AXIN1 ratio are important stoichiometry determinants, the degree of β-catenin incorporation contributes most to the stoichiometry variability (Figure 1G; Table S1).

Taken together, we show that AXIN1 polymerization governs the concentration-dependent assembly of the DC, recruitment of β-catenin, and complex composition.

Oncogenic APC variants are part of the DC

We next assessed the contribution of APC to DC assembly by purifying different DC variants. The kinases robustly co-purified with AXIN1, regardless of APC's presence (Figure 2B, lanes 17 and 13). Although only sub-stoichiometric amounts of β-catenin were recovered in the absence of APC (Figures 2B and 2C, lane 17, and Figure 2D), APC co-expression robustly increased β-catenin co-purification (Figures 2B and 2C, lane 13, and Figure 2D). Analyzing the phosphorylation status of β-catenin using immunoblotting, we observed that APC omission not only limited β-catenin recruitment but also reduced its degron phosphorylation by ≈75% (Figure 2C, compare lanes 13 and 17; Figures 2E and 2F).

Germline and sporadic nonsense mutations give rise to truncated APC variants lacking large C-terminal stretches, with variable losses of AXIN1-binding SAMP repeats and β-catenin-binding 15R and 20R repeats, depending on the truncation site (van Kappel and Maurice, 2017; Zhang and Shay, 2017; Figure 2A). To explore whether these truncated variants can be part of the recombinant DC and facilitate β-catenin recruitment and phosphorylation, we expressed and purified the DC in the context of APC mutant variants found in commonly used CRC cell lines (APC T1556* from COLO-205, APC Q1338* from SW480, and APC S811* from COLO-320DM; Figure 2A; Table S10). All APC variants were expressed (Figures 2B and 2C, lanes 1–12), and APC T1556* and APC Q1338* robustly co-purified with AXIN1 (Figures 2B and 2C, lanes 14 and 15). This was surprising, given the absence of AXIN1-binding SAMP repeats in both these APC variants. APC S811*, although co-purifying at much reduced levels, as anticipated given the loss of all 15R, 20R, and SAMP repeats, nonetheless was detectable within the complexes (Figures 2B and 2C, lane 16). A parallel control pull-down from cells expressing only APC S811* (without any other DC components) indicated that APC S811* inclusion into the DC was specific (Figures 2B and 2C, lane 18). The three truncation variants, including APC S811*, still stimulated β-catenin recruitment (Figures 2B–2D). Both GSK3β- and CK1α-dependent β-catenin phosphorylation were reduced upon APC truncation. This was especially apparent for APC S811*, for which β-catenin phosphorylation was comparable with that observed upon APC omission (Figures 2E and 2F). Expression of APC T1556*, but not APC omission or expression of any other APC variant, reproducibly increased the electrophoretic mobility of AXIN1 in SDS-PAGE, suggesting altered post-translational modification, and lowered levels of AXIN1 and β-catenin (Figures 2B and 2C).

In conclusion, APC truncation variants are hypomorphs still able to incorporate into the DC, in line with previous cell-based biochemical studies (Li et al., 2012; Novellasdemunt et al., 2017; Voloshanenko et al., 2013). APC promotes both the recruitment of β-catenin to the DC and its phosphorylation. Surprisingly, even an extreme CRC mutant variant of APC lacking all β-catenin-binding motifs still supports β-catenin recruitment. Although APC 15R and 20R motifs are therefore not strictly required for facilitating β-catenin recruitment, they play a role in promoting β-catenin phosphorylation.

APC facilitates β-catenin phosphorylation and ubiquitylation

We next aimed to reconstitute the biochemical activities of the DC to investigate the consequences of lost AXIN1 polymerization and oncogenic APC truncation. We individually expressed full-length APC, the aforementioned CRC truncation variants, and an APC variant lacking the so-called β-catenin-inhibitory domain (CID), which is essential for the suppression of β-catenin levels in cells (Choi et al., 2013; Kohler et al., 2009; Liu et al., 2006; Novellasdemunt et al., 2017; Roberts et al., 2011). We separately produced *in vitro*-dephosphorylated β-catenin (Figures 3A and 3B). We further generated an AXIN1-CK1α-GSK3β

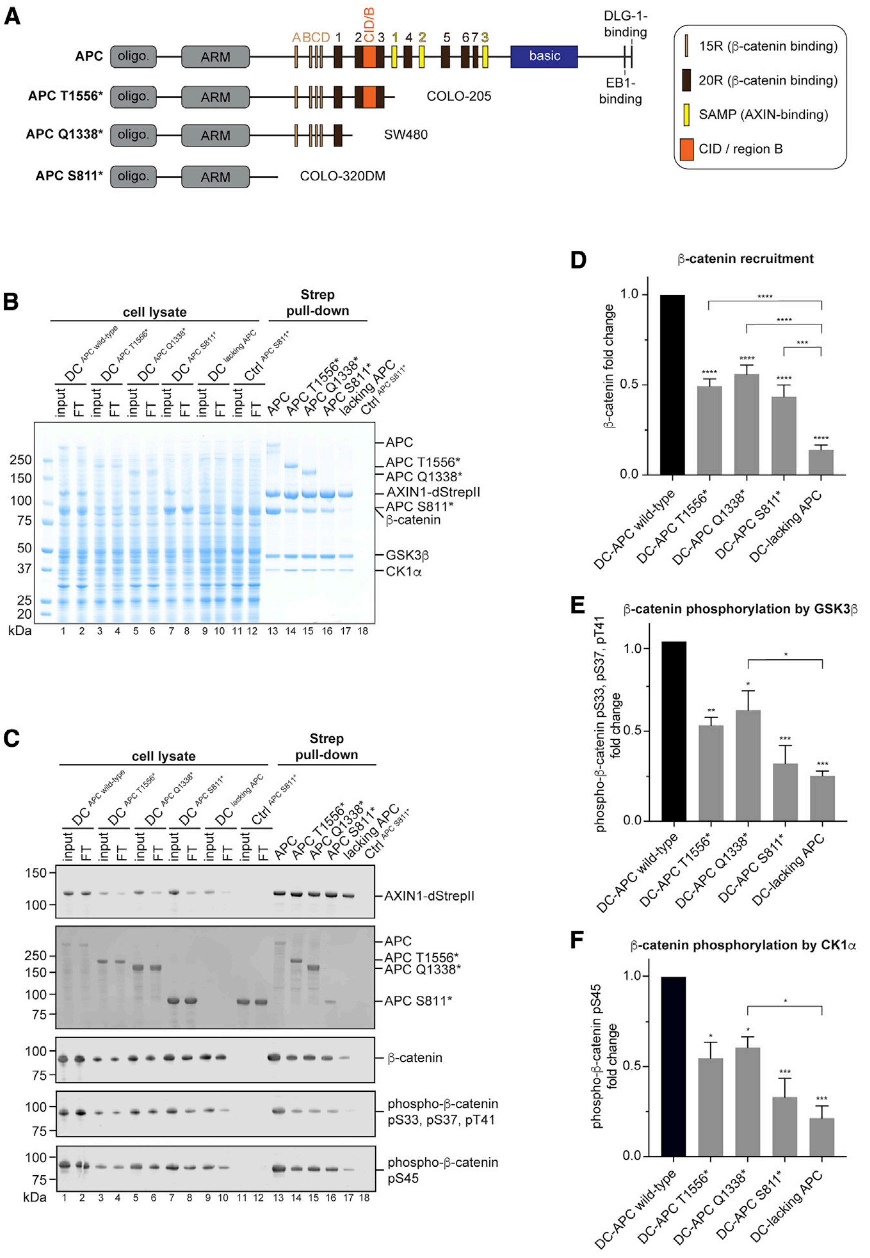

Figure 2. Oncogenic APC variants, although part of the DC, impair β-catenin recruitment and phosphorylation

(A) Schematic representation of APC and its oncogenic truncation variants from the indicated CRC cell lines. See Table S10 for details of APC truncations.

(B) DCs purified via AXIN1-dStrepII with the indicated co-expressed APC variants. Samples were analyzed using SDS-PAGE and Coomassie staining.

(C) Immunoblot analyses of the lysates and affinity-purified material shown in B. Immunoblotting likely underestimates APC (312 kDa) abundance because of lower transfer efficiency compared with truncation variants.

(D) Quantification of total β-catenin recovered with AXIN1, assessed using immunoblotting. Signals were not normalized for β-catenin levels in the lysates, as not all β-catenin may be available for DC binding.

(E and F) Quantification of phosphorylation status (pS33, pS38, pS41; pS45) of recovered β-catenin, normalized for the amounts of recovered total β-catenin.

Results shown in (D)–(F) are means from three independent experiments ± SEM. Statistical analyses were performed using one-way ANOVA with the Bonferroni test for multiple comparisons. Asterisks above bars (without brackets) refer to comparisons to the no-APC condition (black bar). *$p < 0.05$, **$p < 0.01$, ***$p < 0.001$, ****$p < 0.0001$; ns, not significant. See Tables S2–S4 for details of statistical analyses.

(AXIN1-kinase) complex containing either the wild-type proteins, non-polymerizable AXIN1 M3, and/or a kinase-dead (kd) GSK3β variant (KD85/181AN) (Figures 3A, 3B, and S5E). To reconstitute the ubiquitylation reaction, we supplied the E1 ubiquitin-activating enzyme UBE1, the E2 ubiquitin-conjugating enzyme UBCH5a and the E3 ubiquitin ligase complex composed of β-TrCP, SKP1, CUL1, and RBX1, with the latter being *in vitro* neddylated on CUL1 (Rabut and Peter, 2008; Figure 3C). To track β-catenin modification by SDS-PAGE and multi-channel fluorimetry, we labeled β-catenin with an Alexa Fluor 680 fluorophore and used fluorescein-ubiquitin (Figures 3A and 3B). Upon combining the relevant components, we initiated β-catenin phosphorylation and ubiquitylation by ATP addition, terminating the reactions at different time points (see Figures 3D and 3E for 60 min time point and Figures S2 and S3 for additional time points).

The ubiquitylation machinery (E1, E2, E3 complex and ubiquitin) produced free poly-ubiquitin (Figure 3D, lane 2). Unmodified β-catenin appeared as a single band, and addition of the ubiquitylation machinery without the AXIN1-kinase complex resulted in the moderate (mostly mono- and di-) ubiquitylation of β-catenin (Figure 3D, lanes 3 and 4). Further addition of the AXIN1-kinase complex gave rise to β-catenin poly-ubiquitylation (Figure 3D, lane 5), which was further augmented by APC, leading to the near complete depletion of un-modified β-catenin (Figure 3D, lane 6). Given that β-catenin was present in excess (1 μM β-catenin, 150 nM AXIN1-kinase complex, 200 nM APC, and 100 nM $SCF^{β\text{-}TrCP}$), the near stoichiometric poly-ubiquitylation of β-catenin indicates that the reconstituted DC releases β-catenin upon modification and subsequently captures new β-catenin molecules. Although the proportion of modified β-catenin increased, the average ubiquitin chain length decreased in the presence of APC, indicating that APC reduces the processivity of β-catenin ubiquitylation, increasing throughput of the DC (Figure 3D). By

CellPress
OPEN ACCESS

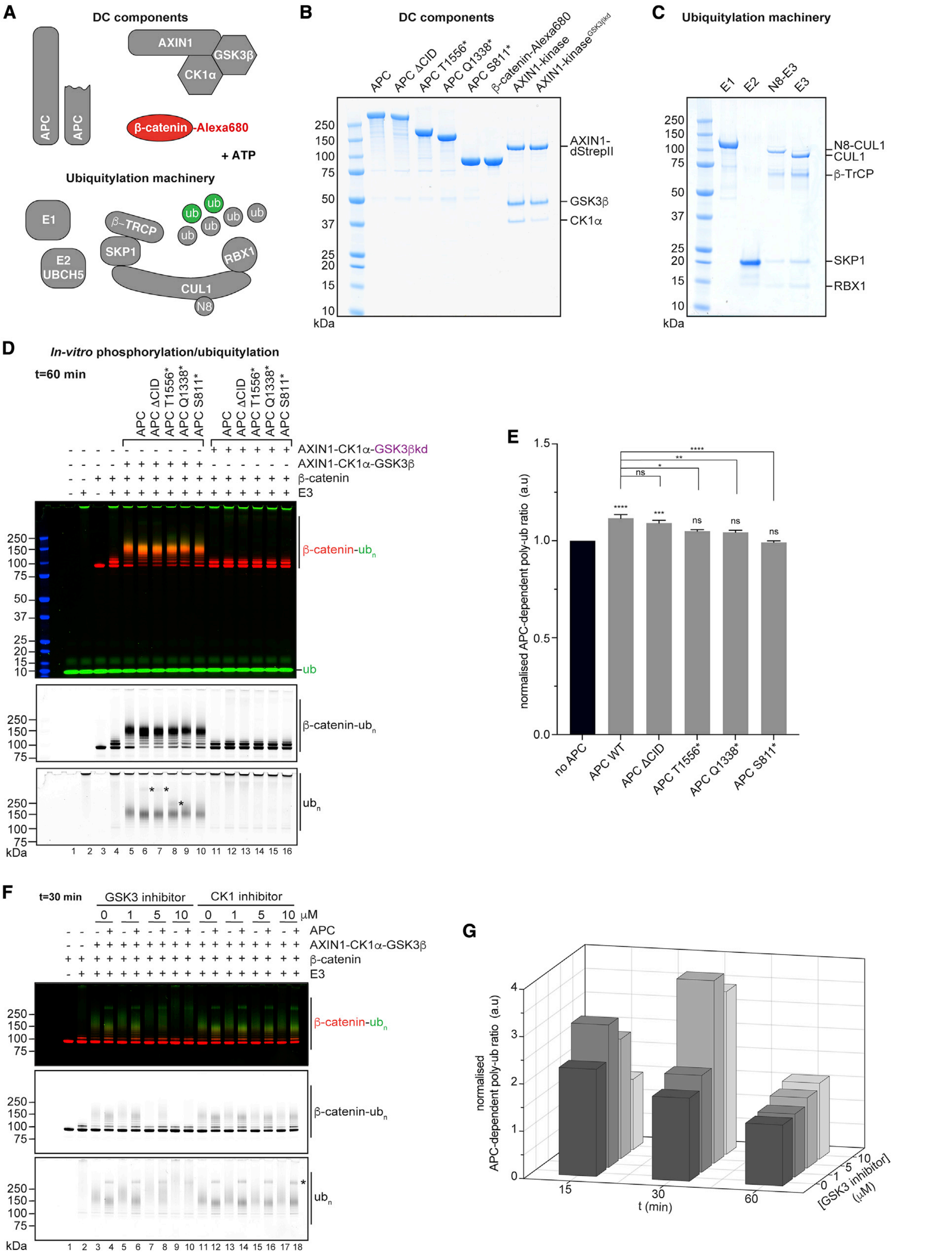

(legend on next page)

dividing the poly-ubiquitylated β-catenin Alexa Fluor 680 signal by the total β-catenin signal in each lane, we compared the extent of β-catenin poly-ubiquitylation between reactions (Figures S2B and S2C). We subsequently quantified the β-catenin poly-ubiquitylation attributed to APC versus AXIN1-kinase complex only (Figure 3D, lanes 6–10 compared with lane 5; Figures 3E and S2D). Although wild-type APC and its ΔCID variant moderately but significantly increased β-catenin poly-ubiquitylation, the APC truncation mutants did not (Figure 3E). We observed a small but statistically significant decrease in β-catenin poly-ubiquitylation with the APC truncations compared with wild-type APC (Figure 3E). Catalytic inactivation of GSK3β resulted in background levels of β-catenin poly-ubiquitylation comparable with those without AXIN1-kinase complex (Figure 3D, compare lanes 11–16 with lane 4). We observed similar trends at the 15 and 30 min reaction time points (Figure S2D).

Interestingly, we noted discrete ubiquitylation at molecular weights corresponding to wild-type, ΔCID, and T1556* APC variants (Figure 3D, lower panel, lanes 6–8), suggesting that APC is a substrate of $SCF^{\beta\text{-TrCP}}$. The β-catenin poly-ubiquitylation signal may mask any potential signal from APC Q1338* and S811*; we were therefore unable to ascertain the ubiquitylation status of these two variants. APC ubiquitylation was dependent on active GSK3β (Figure 3D, lanes 12–14).

We confirmed phosphodegron phosphorylation in poly-ubiquitylated β-catenin using immunoblotting (Figure S3A, lanes 5–10). The much reduced β-catenin phosphorylation kinetics with kinase-dead GSK3β revealed that wild-type and ΔCID APC promote residual β-catenin phosphorylation (Figure S3A, lanes 12 and 13). This led us to optimize our experimental setup, reasoning that the rapid progression of β-catenin phosphorylation with wild-type GSK3β may mask potential effects of APC on β-catenin modification. To attenuate the reaction kinetics while using wild-type GSK3β, we titrated GSK3 or CK1 inhibitors and monitored β-catenin modification across different time points. Indeed, with the GSK3 inhibitor, we observed a dose-dependent suppression in β-catenin phosphorylation and poly-ubiquitylation, which was more extensive in the absence than presence of APC (Figure 3F for 30 min time point; Figures S4A and S4B for 15 and 60 min time points). This sensitization of the assay toward APC addition displayed a maximum at 30 min with 5 μM GSK3 inhibitor (Figure 3G). In contrast, we did not observe this sensitization using a CK1 inhibitor (Figures 3F and S4A–S4C), possibly because of its limited potency toward CK1α (Rena et al., 2004), especially at the high ATP concentration (5 mM) in the reactions.

We next revisited the impact of APC truncations under the sensitized reaction conditions (5 μM GSK3 inhibitor, t = 30 min [Figures 4A and 4B] or t = 60 min [Figures S5A and S5B]). Wild-type and ΔCID APC stimulated β-catenin modification ≈6-fold (Figures 4A and 4B). This stimulation was reduced by ≈50% for the T1556* and Q1338* truncation variants and completely abolished for the S811* variant, with a similar trend after 60 min (Figures 4A, 4B, S5A, and S5B). Under the sensitized conditions, APC retained its ability to limit poly-ubiquitylation processivity (Figure S6A). Although CRC-associated APC truncations were less able to stimulate β-catenin poly-ubiquitylation, APC ΔCID behaved identically to wild-type APC (Figures 4A, 4B, S5A, and S5B), suggesting that the essential role of the CID region in cells (Choi et al., 2013; Kohler et al., 2009; Liu et al., 2006; Novellasdemunt et al., 2017; Roberts et al., 2011) is mediated by other factors not present in our *in vitro* system.

We also probed the contribution of AXIN1 polymerization to the activity of the DC containing either wild-type or truncated APC. Loss of AXIN1 polymerization (M3) significantly reduced the APC-dependent activity of the DC (Figures 4A, 4B, S5A, and S5B). With AXIN1 M3, wild-type and ΔCID APC were only able to stimulate β-catenin ubiquitylation to levels similar to those attained with APC T1556* and APC Q1338* in the context of wild-type AXIN1 (Figures 4A, 4B, S5A, and S5B). Therefore, AXIN1 polymerization is critical to DC activity, in line with the reduced ability of AXIN1 M3 to recruit β-catenin (see above).

Both AXIN1 and β-catenin binding define APC function in the DC

We next explored the mechanism underlying the deficiency of APC truncation variants to promote β-catenin ubiquitylation. Any known APC truncation in CRC removes both a number of AXIN-binding SAMP repeats and 20R β-catenin binding sites (van Kappel and Maurice, 2017; Zhang and Shay, 2017). To dissect the contributions of either AXIN1 or β-catenin binding to APC function, we generated point mutant variants of APC, inactivating either all AXIN1-binding SAMP repeats (Spink et al., 2000) or abolishing the 20R phosphorylation sites responsible for turning 20Rs into high-affinity β-catenin binding sites (Ha et al., 2004; Liu et al., 2006; Table S10). The impairment of

Figure 3. Reconstitution of the biochemical activity of the DC

(A) Schematic representation of the DC and accessory components for biochemical assays.

(B) Purified DC components analyzed using SDS-PAGE and Coomassie staining. kd, kinase-dead.

(C) Purified ubiquitylation machinery components.

(D) *In vitro* DC activity assay (reaction time 60 min). Top panel: dual-channel fluorimetry of SDS-PAGE gel with β-catenin in red and ubiquitin in green. Middle and bottom panels: separated channels. Asterisks in the bottom panel mark ubiquitylated APC variants. See Figure S2 for additional time points and Figure S3 for analyses of β-catenin phosphorylation.

(E) Quantification of APC-dependent β-catenin poly-ubiquitylation. See Figures S2B and S2C for quantification details. Data are means from three independent experiments ± SEM. Statistical significance was tested using one-way ANOVA with the Bonferroni test for multiple comparisons. Asterisks above bars (without brackets) refer to comparisons to the no-APC condition (black bar). *$p < 0.05$, **$p < 0.01$, ***$p < 0.001$, ****$p < 0.0001$; ns, not significant. See Table S5 for details of statistical analyses.

(F) *In vitro* DC activity assay (as in D, but at 30 min time point) ± GSK3 or CK1 inhibitors at the indicated concentrations. The asterisk in the bottom panel marks ubiquitylated APC. See Figures S4A and S4B for additional time points and analyses of β-catenin phosphorylation.

(G) Quantification of APC-dependent β-catenin poly-ubiquitylation ± GSK3 inhibitor, on the basis of data shown in (F) (n = 1 representative experiment). See Figure S4C for CK1 inhibitor quantification.

CellPress
OPEN ACCESS

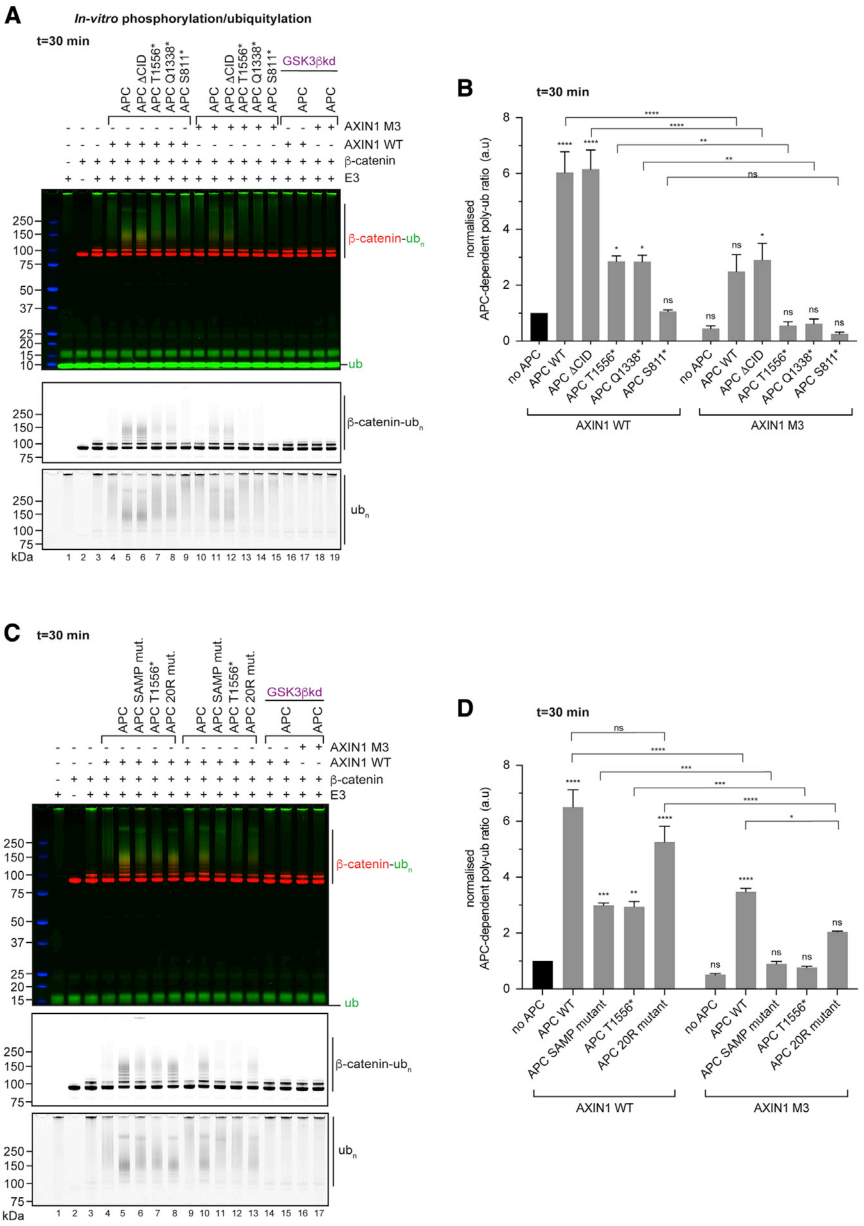

Figure 4. Oncogenic APC truncation and impaired AXIN1 polymerization limit DC activity

(A and C) *In vitro* DC activity assays in the presence of 5 μM GSK3 inhibitor. Data are displayed as in Figure 3D. APC and AXIN1 mutant variants were used as indicated.

(B and D) Quantification of the APC-dependent poly-ubiquitylation of β-catenin. Data are means from three independent experiments ± SEM. Statistical analyses were performed using one-way ANOVA with the Bonferroni test for multiple comparisons. Asterisks above bars (without brackets) refer to comparisons to the no-APC condition (black bar). *p < 0.05, **p < 0.01, ***p < 0.001, ****p < 0.0001; ns, not significant. See Tables S6 and S8 for details of statistical analyses, Figure S5 for data from the 60 min time point and protein quality control, and Figures S6B and S6C for analysis of poly-ubiquitin linkage type.

APC-dependent β-catenin poly-ubiquitylation upon loss of all SAMP repeats was comparable with that seen for APC T1556* (≈50%), which additionally lacks four of the six β-catenin-binding 20R repeats (4–7) (Figures 4C, 4D, and 2A). Both mutants also behaved similarly to each other in the context of non-polymerizable AXIN1 M3, where they were unable to significantly stimulate β-catenin poly-ubiquitylation (Figures 4C and 4D). This suggests that loss of AXIN1 binding is the primary cause for the deficiency of APC T1556* and that additional loss of 20R 4–7 has a limited effect. Concordantly, mutation of all 20R phosphorylation sites accounted only for a small, non-significant reduction of β-catenin poly-ubiquitylation relative to wild-type APC in the context of wild-type AXIN1 and a weaker (≈40%) but significant reduction of β-catenin poly-ubiquitylation in the context of AXIN1 M3 (Figures 4C and 4D). We note that the 20R mutations can, however, be considered a less severe mutation than those of the SAMP repeats. We made similar observations at the 60 min reaction time point (Figures S5C and S5D). The stronger impact of the APC S811* truncation, which removes all direct β-catenin binding sites (Figures 4A, 4B, S5A, and S5B) illustrates that 15Rs and 20Rs nonetheless contribute to APC function in our *in vitro* system, implying possible redundancy. In summary, we show that both AXIN1 and β-catenin binding define the function of APC in the DC and that the loss of APC-AXIN1 interaction largely accounts for the impact of APC truncations that preserve at least some β-catenin binding sites.

AXIN1 polymerization and APC control β-catenin phosphorylation

The contribution of APC toward β-catenin phosphorylation (Figures 2E, 2F, and S3A) prompted us to characterize the β-catenin phosphorylation kinetics using a radiometric assay (Figures 5 and S7; see STAR Methods for details). Our experimental setup did not distinguish between β-catenin degron-specific phosphorylation and potential other phosphorylation events on the protein. We therefore generated a β-catenin phosphodegron mutant variant (4A) as a control (Table S10). These mutations abolished the pS45 and pS33, pS37, pT41 signals corresponding to CK1α- and GSK3β-dependent phosphorylation, respectively (Figure S7A), which is unsurprising, but also very much reduced overall β-catenin phosphorylation, as detected by autoradiography (Figure S7B). This demonstrated that the bulk of β-catenin phosphorylation *in vitro* occurs on the phosphodegron. The autoinhibitory pS9 phosphorylation of GSK3β (Dajani et al., 2001;

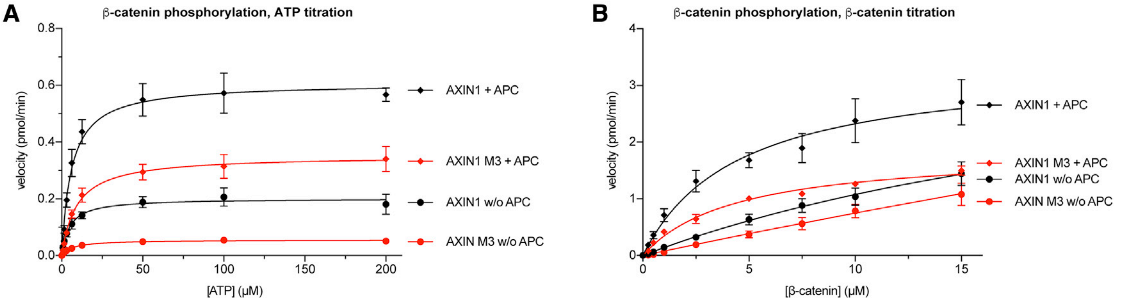

C

variable	reaction	V_{max} (pmol/min)	K_M (μM)	k_{cat} (min^{-1})	k_{cat} / K_M (M^{-1} min^{-1})
[ATP]					
	AXIN1-kinase w/o APC	0.202 ± 0.011	4.92 ± 1.17	0.224 ± 0.013	(4.6 ± 1.1) × 10^4
	AXIN1-kinase + APC	0.607 ± 0.025	6.08 ± 1.03	0.674 ± 0.028	(11 ± 1.9) × 10^4
	AXIN1 M3-kinase w/o APC	0.055 ± 0.003	6.55 ± 1.44	0.061 ± 0.003	(0.9 ± 0.2) × 10^4
	AXIN1 M3-kinase + APC	0.351 ± 0.017	9.29 ± 1.72	0.390 ± 0.018	(4.2 ± 0.8) × 10^4
[β-catenin]					
	AXIN1-kinase w/o APC	n.d.	n.d.	n.d.	(10.4 ± 0.5) × 10^4
	AXIN1-kinase + APC	3.369 ± 0.404	4.49 ± 1.41	3.744 ± 0.448	(83 ± 28) × 10^4
	AXIN1 M3-kinase w/o APC	n.d.	n.d.	n.d.	(7.4 ± 0.4) × 10^4
	AXIN1 M3-kinase + APC	1.856 ± 0.099	4.41 ± 0.62	2.062 ± 0.110	(47 ± 7) × 10^4

Figure 5. AXIN1 polymerization and APC regulate kinase activity in the DC

Kinetic kinase assays to assess β-catenin phosphorylation.

(A) β-Catenin-directed activity of an AXIN1-kinase complex containing either wild-type or polymerization-deficient (M3) AXIN1 ± APC, at different ATP concentrations. See Figure S7 for control reactions and representative autoradiographs. Data points are means from three independent experiments ± SEM.

(B) β-Catenin-directed kinase activity as in (A), but at different β-catenin concentrations.

(C) Summary of kinase activity parameters. Data are means from three independent experiments ± SEM. n.d., not determined because of lack of reaction velocity saturation.

Frame et al., 2001) was barely detected and will therefore have a limited impact on the *in vitro* kinase reactions (Figure S7A).

Titrating ATP while maintaining a fixed concentration of β-catenin and wild-type or M3 AXIN1-kinase complex, we measured the initial rates of β-catenin phosphorylation (Figures 5A, S7C, and S7D). Addition of APC had no (within error) or only a minor effect on the apparent kinase K_M (ATP) in either the wild-type or M3 AXIN1-kinase containing reactions (Figure 5C). Furthermore, K_M (ATP) was comparable for the wild-type and M3 AXIN1-kinase complexes. Therefore, neither inclusion of APC nor AXIN1 polymerization affect ATP binding. In contrast, APC increased the apparent k_{cat} for wild-type and M3 AXIN1-kinase reactions by ≈3- and ≈6-fold and thereby the apparent reaction efficiencies (k_{cat}/K_M) by ≈2- and ≈5-fold, respectively (Figure 5C). In the absence or presence of APC, AXIN1 polymerization increased the apparent reaction efficiency by ≈5- or ≈3-fold, respectively (Figure 5C). The apparent reaction efficiencies of the wild-type AXIN1-kinase complex without APC and the AXIN1 M3-kinase complex with APC were comparable, suggesting that AXIN1 polymerization and APC have overlapping functions (Figure 5C).

We next performed β-catenin titrations at a saturating ATP concentration of 200 μM to explore whether the increased phosphorylation efficiencies are due to an effect of β-catenin binding or another mechanism (Figures 5B, S7E, and S7F). In the absence of APC, the initial velocities of neither the wild-type nor the M3 AXIN1-kinase complex reactions were saturated with up to 15 μM β-catenin (Figure 5B). We could therefore only determine the apparent k_{cat}/K_M reaction efficiencies for these conditions (Figure 5C; see STAR Methods for details). Both the wild-type and M3 AXIN1-kinase complexes experienced a strong (≈8- and ≈6-fold, respectively) increase in reaction efficiencies upon APC addition (Figures 5B and 5C). In the presence of APC, where we could reliably measure all kinetic parameters, AXIN1 polymerization had no effect on the apparent kinase K_M (β-catenin); however, we observed a modest (≈2-fold) increase in k_{cat} and the corresponding reaction efficiency (Figures 5B and 5C). These observations suggest that under the reaction conditions, AXIN1 polymerization promotes β-catenin phosphorylation not through enhancing β-catenin binding but through directly affecting the maximum phosphorylation rates the kinases can attain. This is surprising in the light of our findings described above (Figures 1B, 1C, 1E, and 1G), which would predict a change in K_M.

Taken together, AXIN1 polymerization and APC binding both increase the DC's phosphorylation efficiency. We note that AXIN1 and APC are both CK1α and GSK3β substrates (Ha et al., 2004; Ikeda et al., 1998; Kim et al., 2013; Liu et al., 2006;

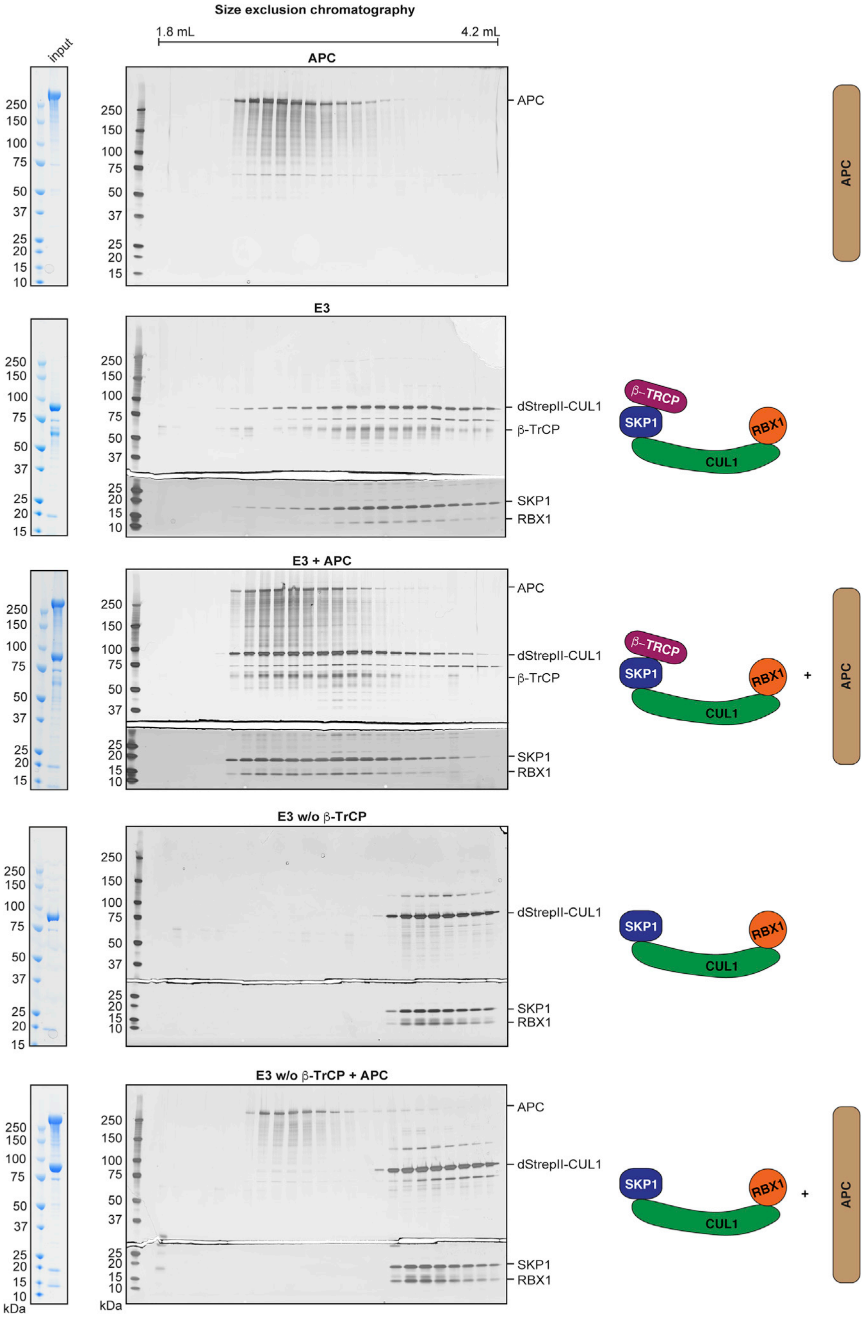

Figure 6. Interaction of APC with the SCF$^{\beta\text{-TrCP}}$ E3 ubiquitin ligase complex

The indicated proteins and complexes were resolved by size exclusion chromatography and fractions analyzed using SDS-PAGE and Coomassie (input) or silver staining (SEC fractions). The top and bottom parts of the gels were separated for silver staining of SKP1 and RBX1 to avoid overstaining of the other components. See Figure S8 for purification of the E3 complexes and analyses of β-catenin and AXIN1 interactions with SCF$^{\beta\text{-TrCP}}$.

Rubinfeld et al., 1996; Figures S7C–S7F) and may act as β-catenin competitors in our assays, complicating the interpretation of the derived parameters.

APC interfaces the DC with the ubiquitylation machinery

Among the many mutually compatible roles proposed for APC in the DC, one is to communicate with SCF$^{\beta\text{-TrCP}}$ (Kimelman and Xu, 2006; Pronobis et al., 2015; Su et al., 2008; Xu and Kimelman, 2007). Reasoning this role may involve a physical interaction of APC with the E3 ubiquitin ligase complex, we performed analytical gel filtration experiments. To benchmark our experimental setup, we confirmed that the elution of phosphorylated but not non-phosphorylated β-catenin is affected by addition of SCF$^{\beta\text{-TrCP}}$ (Figures S8B and S8C), in agreement with the well-understood phosphorylation-dependent interaction of β-catenin with β-TrCP (Wu et al., 2003). The APC elution peak was centered around 2.2 mL and that of SCF$^{\beta\text{-TrCP}}$ around 2.8 mL. Mixing of both components resulted in a substantially earlier elution of SCF$^{\beta\text{-TrCP}}$, in a range similar to that for APC alone (Figure 6), in line with the largely intrinsically disordered APC dominating the elution behavior of an APC-SCF$^{\beta\text{-TrCP}}$ complex. Purifying SCF$^{\beta\text{-TrCP}}$, we obtained two populations, the full E3 complex and one lacking β-TrCP (Figure S8A). This enabled us to test the role of β-TrCP in the E3-APC interaction. Omission of β-TrCP abolished the elution shift by APC (Figure 6), suggesting that the interaction either occurs directly through β-TrCP or through another β-TrCP-dependent mechanism. A recently proposed interaction of *Drosophila* Axin with the β-TrCP ortholog Slimb (Schaefer et al., 2020) prompted us to perform the same experiment with AXIN1. However, we observed only a small population of SCF$^{\beta\text{-TrCP}}$ shifting compared with APC (Figure S8D), indicating that APC is the predominant DC component binding to the E3 complex in our system. Our *in vitro* system will be a valuable tool to decipher the precise molecular mechanisms by which APC communicates the activities of the DC to the ubiquitylation machinery to control Wnt/β-catenin signaling.

Reconstitution of β-catenin proteasomal degradation

To interrogate whether poly-ubiquitylation of β-catenin is sufficient for its proteasomal degradation *in vitro*, we next sought to recapitulate the final step of β-catenin processing, its degradation. We confirmed that β-catenin is indeed modified with

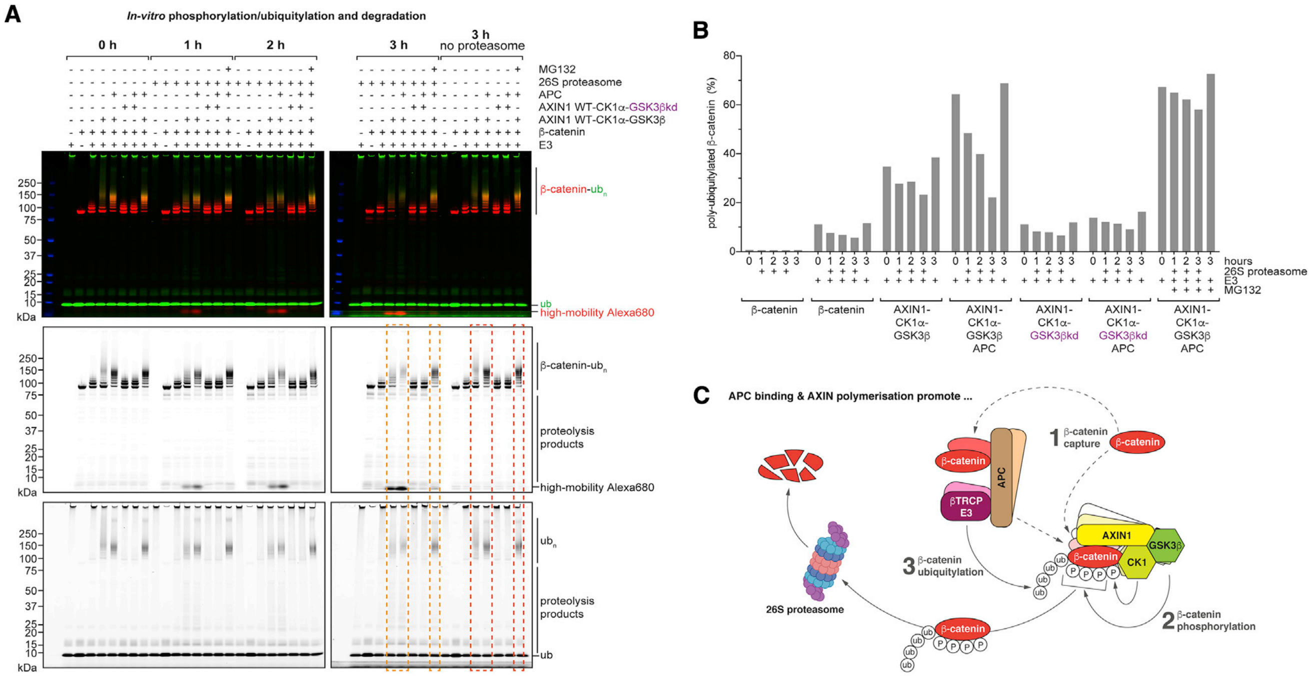

Figure 7. ***In vitro*** **reconstitution of ubiquitylation-dependent β-catenin degradation by the proteasome**

(A) *In vitro* DC activity assay in the presence of 5 μM GSK3 inhibitor. After 60 min, 26S-proteasome was added for the indicated time periods. Data are displayed as in Figure 3D. The high-mobility Alexa 680 signal (bottom of the gel) accumulates in a proteasome- and ubiquitylation-dependent manner. Areas boxed in orange and red-orange highlight the differences in β-catenin levels with or without incubation with proteasome for 3 h, respectively.

(B) Quantification (as in Figure S2B) of the percentage of poly-ubiquitylated β-catenin from the data shown in (A).

(C) Model summarizing functions of AXIN1 polymerization and APC in the DC. APC promotes β-catenin recruitment, both by direct binding and by facilitating β-catenin binding by AXIN1. Likewise, AXIN1 polymerization enhances β-catenin recruitment. Both APC binding and AXIN1 polymerization boost the efficiency of β-catenin phosphorylation. Increased β-catenin phosphorylation translates into increased ubiquitylation of β-catenin. APC may further promote β-catenin ubiquitylation through a direct interaction with $SCF^{\beta\text{-TrCP}}$. Dashed arrows represent interactions, solid arrows enzymatic modifications. The 26S-proteasome was created using BioRender.com.

proteasome-targeting K48-linked ubiquitin chains *in vitro* (Figures S6B and S6C). We next performed β-catenin phosphorylation/ubiquitylation assays, adding purified human 26S-proteasome 60 min after initiating the reactions. As observed previously, the AXIN1-kinase complex and APC stimulated β-catenin poly-ubiquitylation in a GSK3β-dependent manner (Figures 7A and 7B). We observed a time-dependent degradation of poly-ubiquitylated β-catenin by the 26S-proteasome, which was severely inhibited by the proteasome inhibitor MG132 (Figures 7A and 7B). We further noted the proteasome-dependent accumulation of a high-mobility Alexa Fluor 680 degradation product, which correlated with the extent of β-catenin poly-ubiquitylation (Figure 7A). We conclude that our *in vitro* system fully recapitulates the biochemical functions of the core DC from component assembly to phosphorylation-dependent ubiquitylation of β-catenin and its proteasomal degradation.

DISCUSSION

A recent interrogation of the cBioPortal for Cancer Genomics database (Cerami et al., 2012) highlights APC, AXIN2, and β-catenin mutations in ≈67%, ≈5%, and ≈6% of colorectal carcinomas, respectively (Bugter et al., 2021). Other cancer types with DC mutations include hepatocellular, endometrial, adrenocortical, gastric, and bladder urothelial carcinoma as well as melanoma (Bugter et al., 2021). This underpins the significance of understanding the molecular mechanisms of the DC. The biochemical reconstitution of the core DC from purified, recombinant proteins enables detailed mechanistic studies of the DC and its regulators. The latter include protein phosphatases (van Kappel and Maurice, 2017), the poly-ADP-ribosyltransferase tankyrase (Mariotti et al., 2017), ubiquitin ligases (Tauriello and Maurice, 2010), and components of the Wnt signalosome (Gammons and Bienz, 2018).

We show that AXIN1 polymerization is a major determinant of DC assembly and that both AXIN1 polymerization and APC promote β-catenin recruitment, phosphorylation, and ubiquitylation. Despite the loss of all AXIN1-binding SAMP repeats and numerous or all known β-catenin binding sites, APC truncation variants found in CRC are still part of the DC and act as functional hypomorphs. Stimulation of β-catenin ubiquitylation by AXIN1 polymerization and APC correlates with β-catenin phosphorylation. Beyond a simple phosphorylation-dependent effect, APC may promote β-catenin ubiquitylation by recruiting the $SCF^{\beta\text{-TrCP}}$ complex to the DC. These insights led us to propose a model describing roles of polymeric AXIN1 and APC in the DC (Figure 7C). We further reconstituted the ubiquitylation- and proteasome-dependent degradation of β-catenin, enabling future investigations of DC-dependent

CellPress
OPEN ACCESS

β-catenin processing at every step, namely recruitment, phosphorylation, ubiquitylation, and proteolysis.

DC assembly and stoichiometry

We observe a concentration-dependent contribution of AXIN1 polymerization to the overall size of the DC. Rather than describing the DC as a stoichiometrically well-defined multi-protein machine, it is more appropriately portrayed as a dynamic assembly whose composition responds to the concentration of its components, in line with previous proposals (Schaefer et al., 2018; Xue et al., 2013). The molecular weights of the complexes attainable indicate that the extent of AXIN1 polymerization is limited in our system, which agrees with the relatively high (low-micromolar) K_d of AXIN1 DIX domains (Kan et al., 2020). Using parameters derived from SEC-MALS, we calculated the DCs extinction coefficients and concentrations across the SEC-MALS elution peaks (see STAR Methods). For the DC with wild-type AXIN1, the estimated concentration in the input sample was 415 nM; its maximum concentration at the detector was 56 nM. In SEC-MALS experiments performed on the isolated AXIN1 DIX domain at >10-fold higher protein concentration (6.6 μM in the input and 0.71 μM at the detector), molecular weights compatible with only 1 or 2 protomers were observed (Kan et al., 2020). Despite the comparably low DC concentrations in our study, we still observe a contribution of AXIN1 polymerization to the size of the DC, both in SEC-MALS and even in mass photometry, likely because of avidity provided by APC. The main consequence of AXIN1 polymerization is an increased recruitment of β-catenin to the DC. Although our current setup does not lend itself to the precise determination of DC stoichiometries, we used *in silico* modeling to determine a subset of possible DC stoichiometries that satisfy the observed molecular weights. Our analysis indicates that AXIN1 polymerization provides the DC with a broader range of possible stoichiometries and increased β-catenin recruitment. Our model also reveals potential stoichiometries at which the wild-type DC is nearly or fully saturated with β-catenin.

These stoichiometries obtained *in vitro* may differ from those in cells, where DC-accessory factors, additional regulatory events, potential effects of molecular crowding (Rivas and Minton, 2016), and the subcellular context add to the complexity. Indeed, our work and that of Kan et al. (2020) points toward stoichiometry differences between DCs assembled *in vitro* and those observed in cells by light microscopy (Schaefer et al., 2018). Cytoplasmic puncta in the *Wingless* interstripes within the epidermis of *Drosophila* embryos, equivalents of the DC known as β-catenin degradasomes, were measured to contain tens to hundreds of Axin molecules (Schaefer et al., 2018). DC-associated factors influencing the size of the DC may include the poly-ADP-ribosyltransferase tankyrase (Martino-Echarri et al., 2016; Mariotti et al., 2017) or the Hippo signaling protein YAP/TAZ, which has been proposed to complex with the DC (Azzolin et al., 2014). These and other mechanisms may lead to still higher local concentrations of AXIN1 in cells than those we achieved in this study.

Overlapping functions of AXIN1 polymerization and APC

The functional overlap of AXIN1 polymerization and APC in DC activities illustrates the redundancy and robustness inherent to the DC (Pronobis et al., 2017). Although loss of AXIN1 polymerization reduces DC activity, APC can still stimulate the complex. This partial mechanistic redundancy likely ensures that the DC efficiently maintains low levels of newly synthesized β-catenin, given the large impact leaky β-catenin target gene expression would have on cellular decision making.

Functions of AXIN1 polymers

Polymerization of AXIN1 is required for its function in the Wnt/β-catenin pathway (Fiedler et al., 2011; Schwarz-Romond et al., 2007) and, as we show here, for normal DC assembly. AXIN1 polymerization likely increases its avidity for other DC components. This was apparent in our pull-down experiments for β-catenin, but lower experimental concentrations of components may be needed to reveal a potential avidity effect in APC recruitment, particularly if the AXIN1-APC interaction is of higher affinity. In turn, clustering of AXIN1 by APC may support AXIN1 polymerization. A contribution of Apc to the formation cytoplasmic Axin puncta has been revealed in *Apc* null *Drosophila* tissues, in line with roles for both Axin and Apc in the formation of polymeric assemblies (Mendoza-Topaz et al., 2011). AXIN1 has been proposed to adopt different conformations (Kim et al., 2013; Wang et al., 2013), and we speculate that polymerization may, in addition to increasing local AXIN1 concentration, allosterically expose the AXIN1 β-catenin binding site. However, in the light of enhanced β-catenin binding conferred by AXIN1 polymerization (Figures 1B, 1C, 1E, and 1G), it is surprising that AXIN1 polymerization promotes β-catenin phosphorylation through increasing k_{cat} rather than decreasing K_M. Future studies will address the underlying mechanism.

Functions of APC

Many non-mutually exclusive functions have been postulated for APC (Gammons and Bienz, 2018; Ha et al., 2004; Kimelman and Xu, 2006; Krieghoff et al., 2006; Kunttas-Tatli et al., 2014; Mendoza-Topaz et al., 2011; Novellasdemunt et al., 2017; Pronobis et al., 2017; Roberts et al., 2011; Stamos and Weis, 2013; Su et al., 2008; Tacchelly-Benites et al., 2018; van Kappel and Maurice, 2017). Our *in vitro* system offers novel opportunities for studying the precise effects of APC on DC assembly and its biochemical activities.

DC assembly

As does AXIN1 polymerization, APC promotes β-catenin recruitment to the DC, in agreement with previous proposals and the notion that β-catenin-binding 15R and 20R motifs in the extensive intrinsically disordered regions of APC act like a ‘‘fishing net’’ to retrieve cytoplasmic β-catenin (Ha et al., 2004; Kimelman and Xu, 2006; van Kappel and Maurice, 2017). APC truncation variants observed in CRC are still incorporated into the DC, as observed in cellular biochemistry studies (Li et al., 2012; Novellasdemunt et al., 2017; Voloshanenko et al., 2013). With loss of SAMP repeats in APC T1556* and APC Q1338* (Figure 2A), we hypothesize that the interaction of these APC variants with AXIN1 occurs largely indirectly through β-catenin. Indeed, β-catenin has been shown to simultaneously bind AXIN1 and a non-phosphorylated 20R3 motif from APC but not its phosphorylated variant, in agreement with structural findings (Ha et al., 2004).

APC 15R motifs are expected to bind β-catenin simultaneously with AXIN1 (Ha et al., 2004).

Surprisingly, even the most extensive truncation mutant tested (APC S811*, found in COLO-320DM cells) can be part of the DC and promote β-catenin recruitment. How APC S811* can achieve this in the absence of known β-catenin binding sites remains unknown. APC S811*, and also the other APC variants tested, may bind AXIN1 through its armadillo repeat domain (Pronobis et al., 2015), and this APC-AXIN1 interaction may promote a conformational change in AXIN1 that enables β-catenin binding to AXIN1 (Kim et al., 2013; Wang et al., 2013). The partially retained function of APC S811* offers a potential explanation for why a complete loss of APC is selected against during colorectal carcinogenesis (Albuquerque et al., 2002).

APC and enzymatic functions of the DC

We find that APC promotes both β-catenin phosphorylation and ubiquitylation. Given the absence of DC accessory components in our setup, this cannot be explained by APC-dependent protection of β-catenin from de-phosphorylation (Su et al., 2008) or de-ubiquitylation (Novellasdemunt et al., 2017), or the partition of the DC from the Wnt signalosome (Mendoza-Topaz et al., 2011), although each of these scenarios is likely relevant in the cellular context. Our mutagenesis studies illustrate the importance of APC's AXIN1 and β-catenin binding sites for DC-dependent modification of β-catenin.

Phosphorylation. We unequivocally show that APC promotes β-catenin phosphorylation, clarifying previous uncertainties around this question (e.g., Novellasdemunt et al., 2017). We find that neither APC incorporation nor AXIN1 polymerization affect K_M (ATP) and thereby ATP binding by the kinases. Instead, both APC binding and AXIN1 polymerization stimulate k_{cat}, leading to an enhanced catalytic efficiency k_{cat}/K_M. As we did not achieve saturation of the initial β-catenin phosphorylation velocities when titrating β-catenin *in vitro*, we could not deduce whether APC renders β-catenin phosphorylation more efficient by lowering the K_M (β-catenin), as our pull-down experiments would suggest. Our inability to achieve saturation of β-catenin phosphorylation rates by titrating β-catenin may reflect distinct reaction conditions from those used by Gavagan et al. (2020); this includes the presence of full-length AXIN1 and APC, which are also substrates of CK1α and GSK3β (Ha et al., 2004; Ikeda et al., 1998; Kim et al., 2013; Liu et al., 2006; Rubinfeld et al., 1996).

Ubiquitylation. We observe that APC limits the ubiquitin chain length on β-catenin, and thereby processivity, while increasing the stoichiometry of β-catenin ubiquitylation, indicating that APC promotes the flux of β-catenin through the DC and $SCF^{\beta\text{-}TrCP}$. Further supporting a role of APC in communicating with the ubiquitylation machinery, we show that APC interacts with the $SCF^{\beta\text{-}TrCP}$ complex independently of β-catenin and that this interaction is mediated by β-TrCP. AXIN1, however, appears to interact with $SCF^{\beta\text{-}TrCP}$ much more weakly than APC, at least under our experimental conditions. These observations differ from those of Schaefer et al. (2020), in which the *Drosophila* β-TrCP ortholog Slimb was shown to co-localize and co-immunoprecipitate with Axin but not Apc2. Our data further suggest that APC is a substrate of $SCF^{\beta\text{-}TrCP}$, potentially being mono-ubiquitylated (Figure S6B), with hitherto unknown functional consequences.

Role of the 20R2-CID region of APC

Previous studies have revealed that the 20R2-CID region in APC is critical for β-catenin ubiquitylation and degradation (Choi et al., 2013; Kohler et al., 2009; Liu et al., 2006; Novellasdemunt et al., 2017; Roberts et al., 2011). As 20R2-CID does not bind β-catenin, the underlying mechanism has remained unclear. In contrast to these cell-based studies, our *in vitro* system does not ascribe a function to the CID, strongly suggesting that the core DC lacks additional factors or the cellular context required for the CID to fulfil its role. Indeed, several studies proposed additional regulators that may act through the 20R2-CID region, including the serine/threonine protein phosphatase PP1, α-catenin, and the de-ubiquitylating enzyme USP7 (Choi et al., 2013; Novellasdemunt et al., 2017; Su et al., 2008).

In summary, the reconstitution of the DC provides detailed insights into the functions of the DC scaffolds AXIN1 and APC and opens new avenues for the mechanistic dissection of Wnt/β-catenin signaling. The system further provides important tools for the identification and development of molecular probes and potential therapeutics to modulate this highly cancer-relevant pathway.

Limitations of the study

This study presents a prototypic application of our reductionist system to interrogate the DC at mechanistic detail. For technical reasons, the experiments were carried out at higher than cellular concentrations of the relevant proteins (Lee et al., 2003; Tan et al., 2012), which in part may limit or override the requirement for scaffolding to drive interactions or biochemical reactions. Where DC core components were co-expressed prior to their purification, the cellular context may still affect their interplay. Similarly, the phosphorylation status of AXIN1 and APC, known to regulate the DC (van Kappel and Maurice, 2017), was not controlled in our assays. AXIN and APC paralogs (AXIN2, APC2) will also need to be considered. Future developments of the system will address these and other limitations referred to above.

STAR★METHODS

Detailed methods are provided in the online version of this paper and include the following:

- KEY RESOURCES TABLE
- RESOURCE AVAILABILITY
  - Lead contact
  - Materials availability
  - Data and code availability
- EXPERIMENTAL MODEL AND SUBJECT DETAILS
- METHOD DETAILS
  - Cloning and expression of recombinant proteins and complexes
  - APC purification
  - β-catenin purification
  - β-catenin-ybbR Alexa Fluor® 680 labeling
  - Purification of the destruction complex and subcomplexes
  - $SCF^{\beta\text{-}TrCP}$ E3 ligase complex purification and in-vitro neddylation

CellPress
OPEN ACCESS

- ○ E1 and E2 purifications
- ○ SEC-MALS
- ○ Assessment of $SCF^{\beta\text{-TrCP}}$ E3 ligase interactions with DC components
- ○ β-catenin phosphorylation and ubiquitylation reactions
- ○ *In-vitro* phosphorylation and de-phosphorylation
- ○ β-catenin proteasomal degradation
- ○ Radiometric kinetic kinase assays
- ○ Mass photometry
- ○ *In-silico* stoichiometry modeling of the DC and estimation of DC molar concentrations
- ○ Antibodies and reagents
- ● QUANTIFICATION AND STATISTICAL ANALYSIS

SUPPLEMENTAL INFORMATION

Supplemental information can be found online at https://doi.org/10.1016/j.molcel.2021.07.013.

ACKNOWLEDGMENTS

We thank Gideon Coster and Jerome Gouge for λ-phosphatase, Erin Cutts for Sfp1, Aylin Morris-Davies and Edward Morris for UBA1, and Frank Sicheri for the UBCH5C expression construct. We thank Jan-Michael Peters (IMP, Vienna, Austria) for providing the biGBac system. We thank Refeyn, particularly Tomás de Garay, for help with mass photometry. We thank Elton Zeqiraj, Sonja Lorenz, Gideon Coster, and members of the S.G. team for helpful comments on the manuscript. Work in S.G.'s laboratory has been funded by Cancer Research UK, first through a Career Establishment Award (C47521/A16217), followed by a Programme Foundation Award (C47521/A28286), by the Wellcome Trust through an Investigator Award (214311/Z/18/Z), by the Lister Institute of Preventive Medicine through a Lister Institute Research Prize Fellowship, and by the Institute of Cancer Research (ICR).

AUTHOR CONTRIBUTIONS

S.G. conceived the project with input from M.R. S.G. and M.R. planned and M.R. conducted all experiments. M.Z. expressed and purified the E3 complex. S.S. performed the *in silico* DC stoichiometry analysis. R.K. cloned the DC expression construct. S.G. and M.R. wrote the manuscript with contributions from all authors.

DECLARATION OF INTERESTS

The authors declare no competing interests.

INCLUSION AND DIVERSITY

One or more of the authors of this paper self-identifies as an underrepresented ethnic minority in science.

Received: July 13, 2020
Revised: May 18, 2021
Accepted: July 13, 2021
Published: August 4, 2021

SUPPORTING CITATIONS

The following references appear in the Supplemental information: Morrone et al. (2012).

REFERENCES

Aberle, H., Bauer, A., Stappert, J., Kispert, A., and Kemler, R. (1997). Beta-catenin is a target for the ubiquitin-proteasome pathway. EMBO J. *16*, 3797–3804.

Albuquerque, C., Breukel, C., van der Luijt, R., Fidalgo, P., Lage, P., Slors, F.J., Leitão, C.N., Fodde, R., and Smits, R. (2002). The 'just-right' signaling model: APC somatic mutations are selected based on a specific level of activation of the beta-catenin signaling cascade. Hum. Mol. Genet. *11*, 1549–1560.

Amit, S., Hatzubai, A., Birman, Y., Andersen, J.S., Ben-Shushan, E., Mann, M., Ben-Neriah, Y., and Alkalay, I. (2002). Axin-mediated CKI phosphorylation of beta-catenin at Ser 45: a molecular switch for the Wnt pathway. Genes Dev. *16*, 1066–1076.

Azzolin, L., Panciera, T., Soligo, S., Enzo, E., Bicciato, S., Dupont, S., Bresolin, S., Frasson, C., Basso, G., Guzzardo, V., et al. (2014). YAP/TAZ incorporation in the β-catenin destruction complex orchestrates the Wnt response. Cell *158*, 157–170.

Barker, N., and Clevers, H. (2006). Mining the Wnt pathway for cancer therapeutics. Nat. Rev. Drug Discov. *5*, 997–1014.

Barua, D., and Hlavacek, W.S. (2013). Modeling the effect of APC truncation on destruction complex function in colorectal cancer cells. PLoS Comput. Biol. *9*, e1003217.

Behrens, J., Jerchow, B.A., Würtele, M., Grimm, J., Asbrand, C., Wirtz, R., Kühl, M., Wedlich, D., and Birchmeier, W. (1998). Functional interaction of an axin homolog, conductin, with beta-catenin, APC, and GSK3beta. Science *280*, 596–599.

Berman, H.M., Battistuz, T., Bhat, T.N., Bluhm, W.F., Bourne, P.E., Burkhardt, K., Feng, Z., Gilliland, G.L., Iype, L., Jain, S., et al. (2002). The Protein Data Bank. Acta Crystallogr. D Biol. Crystallogr. *58*, 899–907.

Bieniossek, C., Richmond, T.J., and Berger, I. (2008). MultiBac: multigene baculovirus-based eukaryotic protein complex production. Curr. Protoc. Protein Sci. *Chapter 5*. Unit 5.20.

Bray, F., Ferlay, J., Soerjomataram, I., Siegel, R.L., Torre, L.A., and Jemal, A. (2018). Global cancer statistics 2018: GLOBOCAN estimates of incidence and mortality worldwide for 36 cancers in 185 countries. CA Cancer J. Clin. *68*, 394–424.

Bugter, J.M., Fenderico, N., and Maurice, M.M. (2021). Mutations and mechanisms of WNT pathway tumour suppressors in cancer. Nat. Rev. Cancer *21*, 5–21.

Cerami, E., Gao, J., Dogrusoz, U., Gross, B.E., Sumer, S.O., Aksoy, B.A., Jacobsen, A., Byrne, C.J., Heuer, M.L., Larsson, E., et al. (2012). The cBio cancer genomics portal: an open platform for exploring multidimensional cancer genomics data. Cancer Discov. *2*, 401–404.

Choi, S.H., Estarás, C., Moresco, J.J., Yates, J.R., 3rd, and Jones, K.A. (2013). α-Catenin interacts with APC to regulate β-catenin proteolysis and transcriptional repression of Wnt target genes. Genes Dev. *27*, 2473–2488.

Chou, Y.-C., Keszei, A.F.A., Rohde, J.R., Tyers, M., and Sicheri, F. (2012). Conserved structural mechanisms for autoinhibition in IpaH ubiquitin ligases. J. Biol. Chem. *287*, 268–275.

Clevers, H., Loh, K.M., and Nusse, R. (2014). An integral program for tissue renewal and regeneration: Wnt signaling and stem cell control. Science *346*, 1248012.

Dajani, R., Fraser, E., Roe, S.M., Yeo, M., Good, V.M., Thompson, V., Dale, T.C., and Pearl, L.H. (2003). Structural basis for recruitment of glycogen synthase kinase 3beta to the axin-APC scaffold complex. EMBO J. *22*, 494–501.

Dajani, R., Fraser, E., Roe, S.M., Young, N., Good, V., Dale, T.C., and Pearl, L.H. (2001). Crystal structure of glycogen synthase kinase 3 beta: structural basis for phosphate-primed substrate specificity and autoinhibition. Cell *105*, 721–732.

Eklof Spink, K., Fridman, S.G., and Weis, W.I. (2001). Molecular mechanisms of beta-catenin recognition by adenomatous polyposis coli revealed by the structure of an APC-beta-catenin complex. EMBO J. *20*, 6203–6212.

Fiedler, M., Mendoza-Topaz, C., Rutherford, T.J., Mieszczanek, J., and Bienz, M. (2011). Dishevelled interacts with the DIX domain polymerization interface of Axin to interfere with its function in down-regulating β-catenin. Proc. Natl. Acad. Sci. U S A *108*, 1937–1942.

Frame, S., Cohen, P., and Biondi, R.M. (2001). A common phosphate binding site explains the unique substrate specificity of GSK3 and its inactivation by phosphorylation. Mol. Cell *7*, 1321–1327.

Gammons, M., and Bienz, M. (2018). Multiprotein complexes governing Wnt signal transduction. Curr. Opin. Cell Biol. *51*, 42–49.

Gasteiger, E., Hoogland, C., Gattiker, A., Duvaud, S., Wilkins, M.R., Appel, R.D., and Bairoch, A. (2005). Protein identification and analysis tools on the ExPASy server. In The Proteomics Protocols Handbook, J.M. Walker, ed. (Humana Press), pp. 571–607.

Gavagan, M., Fagnan, E., Speltz, E.B., and Zalatan, J.G. (2020). The scaffold protein Axin promotes signaling specificity within the Wnt pathway by suppressing competing kinase reactions. Cell Syst. *10*, 515–525.e5.

Ha, N.-C., Tonozuka, T., Stamos, J.L., Choi, H.-J., and Weis, W.I. (2004). Mechanism of phosphorylation-dependent binding of APC to beta-catenin and its role in beta-catenin degradation. Mol. Cell *15*, 511–521.

Hamada, F., Tomoyasu, Y., Takatsu, Y., Nakamura, M., Nagai, S., Suzuki, A., Fujita, F., Shibuya, H., Toyoshima, K., Ueno, N., and Akiyama, T. (1999). Negative regulation of Wingless signaling by D-axin, a Drosophila homolog of axin. Science *283*, 1739–1742.

Ikeda, S., Kishida, S., Yamamoto, H., Murai, H., Koyama, S., and Kikuchi, A. (1998). Axin, a negative regulator of the Wnt signaling pathway, forms a complex with GSK-3beta and beta-catenin and promotes GSK-3beta-dependent phosphorylation of beta-catenin. EMBO J. *17*, 1371–1384.

Jiang, J., and Struhl, G. (1998). Regulation of the Hedgehog and Wingless signalling pathways by the F-box/WD40-repeat protein Slimb. Nature *391*, 493–496.

Kan, W., Enos, M.D., Korkmazhan, E., Muennich, S., Chen, D.-H., Gammons, M.V., Vasishtha, M., Bienz, M., Dunn, A.R., Skiniotis, G., and Weis, W.I. (2020). Limited dishevelled/Axin oligomerization determines efficiency of Wnt/β-catenin signal transduction. eLife *9*, 531.

Kim, S.-E., Huang, H., Zhao, M., Zhang, X., Zhang, A., Semonov, M.V., MacDonald, B.T., Zhang, X., Zhang, X., Garcia Abreu, J., et al. (2013). Wnt stabilization of β-catenin reveals principles for morphogen receptor-scaffold assemblies. Science *340*, 867–870.

Kimelman, D., and Xu, W. (2006). Beta-catenin destruction complex: insights and questions from a structural perspective. Oncogene *25*, 7482–7491.

Kishida, S., Yamamoto, H., Ikeda, S., Kishida, M., Sakamoto, I., Koyama, S., and Kikuchi, A. (1998). Axin, a negative regulator of the wnt signaling pathway, directly interacts with adenomatous polyposis coli and regulates the stabilization of beta-catenin. J. Biol. Chem. *273*, 10823–10826.

Kohler, E.M., Derungs, A., Daum, G., Behrens, J., and Schneikert, J. (2008). Functional definition of the mutation cluster region of adenomatous polyposis coli in colorectal tumours. Hum. Mol. Genet. *17*, 1978–1987.

Kohler, E.M., Chandra, S.H.V., Behrens, J., and Schneikert, J. (2009). Beta-catenin degradation mediated by the CID domain of APC provides a model for the selection of APC mutations in colorectal, desmoid and duodenal tumours. Hum. Mol. Genet. *18*, 213–226.

Krieghoff, E., Behrens, J., and Mayr, B. (2006). Nucleo-cytoplasmic distribution of β-catenin is regulated by retention. J. Cell Sci. *119*, 1453–1463.

Kunttas-Tatli, E., Roberts, D.M., and McCartney, B.M. (2014). Self-association of the APC tumor suppressor is required for the assembly, stability, and activity of the Wnt signaling destruction complex. Mol. Biol. Cell *25*, 3424–3436.

Lee, E., Salic, A., Krüger, R., Heinrich, R., and Kirschner, M.W. (2003). The roles of APC and Axin derived from experimental and theoretical analysis of the Wnt pathway. PLoS Biol. *1*, E10.

Li, V.S.W., Ng, S.S., Boersema, P.J., Low, T.Y., Karthaus, W.R., Gerlach, J.P., Mohammed, S., Heck, A.J.R., Maurice, M.M., Mahmoudi, T., and Clevers, H. (2012). Wnt signaling through inhibition of β-catenin degradation in an intact Axin1 complex. Cell *149*, 1245–1256.

Liu, C., Li, Y., Semenov, M., Han, C., Baeg, G.-H., Tan, Y., Zhang, Z., Lin, X., and He, X. (2002). Control of β-catenin phosphorylation/degradation by a dual-kinase mechanism. Cell *108*, 837–847.

Liu, J., Xing, Y., Hinds, T.R., Zheng, J., and Xu, W. (2006). The third 20 amino acid repeat is the tightest binding site of APC for beta-catenin. J. Mol. Biol. *360*, 133–144.

Mariotti, L., Pollock, K., and Guettler, S. (2017). Regulation of Wnt/β-catenin signalling by tankyrase-dependent poly(ADP-ribosyl)ation and scaffolding. Br. J. Pharmacol. *174*, 4611–4636.

Martino-Echarri, E., Brocardo, M.G., Mills, K.M., and Henderson, B.R. (2016). Tankyrase inhibitors stimulate the ability of tankyrases to bind Axin and drive assembly of β-catenin degradation-competent Axin puncta. PLoS ONE *11*, e0150484.

Mendoza-Topaz, C., Mieszczanek, J., and Bienz, M. (2011). The adenomatous polyposis coli tumour suppressor is essential for Axin complex assembly and function and opposes Axin's interaction with Dishevelled. Open Biol. *1*, 110013.

Morin, P.J., Sparks, A.B., Korinek, V., Barker, N., Clevers, H., Vogelstein, B., and Kinzler, K.W. (1997). Activation of β-catenin-Tcf signaling in colon cancer by mutations in β-catenin or APC. Science *275*, 1787–1790.

Morrone, S., Cheng, Z., Moon, R.T., Cong, F., and Xu, W. (2012). Crystal structure of a Tankyrase-Axin complex and its implications for Axin turnover and Tankyrase substrate recruitment. Proc. Natl. Acad. Sci. U S A *109*, 1500–1505.

Munemitsu, S., Albert, I., Souza, B., Rubinfeld, B., and Polakis, P. (1995). Regulation of intracellular beta-catenin levels by the adenomatous polyposis coli (APC) tumor-suppressor protein. Proc. Natl. Acad. Sci. U S A *92*, 3046–3050.

Nong, J., Kang, K., Shi, Q., Zhu, X., Tao, Q., and Chen, Y.-G. (2021). Phase separation of Axin organizes the β-catenin destruction complex. J. Cell Biol. *220*, e202012112.

Novellasdemunt, L., Foglizzo, V., Cuadrado, L., Antas, P., Kucharska, A., Encheva, V., Snijders, A.P., and Li, V.S.W. (2017). USP7 is a tumor-specific WNT activator for APC-mutated colorectal cancer by mediating β-catenin deubiquitination. Cell Rep. *21*, 612–627.

Orford, K., Crockett, C., Jensen, J.P., Weissman, A.M., and Byers, S.W. (1997). Serine phosphorylation-regulated ubiquitination and degradation of beta-catenin. J. Biol. Chem. *272*, 24735–24738.

Pronobis, M.I., Rusan, N.M., and Peifer, M. (2015). A novel GSK3-regulated APC:Axin interaction regulates Wnt signaling by driving a catalytic cycle of efficient βcatenin destruction. eLife *4*, e08022.

Pronobis, M.I., Deuitch, N., Posham, V., Mimori-Kiyosue, Y., and Peifer, M. (2017). Reconstituting regulation of the canonical Wnt pathway by engineering a minimal β-catenin destruction machine. Mol. Biol. Cell *28*, 41–53.

Rabut, G., and Peter, M. (2008). Function and regulation of protein neddylation. 'Protein modifications: beyond the usual suspects' review series. EMBO Rep. *9*, 969–976.

Rena, G., Bain, J., Elliott, M., and Cohen, P. (2004). D4476, a cell-permeant inhibitor of CK1, suppresses the site-specific phosphorylation and nuclear exclusion of FOXO1a. EMBO Rep. *5*, 60–65.

Rivas, G., and Minton, A.P. (2016). Macromolecular crowding in vitro, in vivo, and in between. Trends Biochem. Sci. *41*, 970–981.

Roberts, D.M., Pronobis, M.I., Poulton, J.S., Waldmann, J.D., Stephenson, E.M., Hanna, S., and Peifer, M. (2011). Deconstructing the ßcatenin destruction complex: mechanistic roles for the tumor suppressor APC in regulating Wnt signaling. Mol. Biol. Cell *22*, 1845–1863.

Rubinfeld, B., Souza, B., Albert, I., Müller, O., Chamberlain, S.H., Masiarz, F.R., Munemitsu, S., and Polakis, P. (1993). Association of the APC gene product with beta-catenin. Science *262*, 1731–1734.

Rubinfeld, B., Albert, I., Porfiri, E., Fiol, C., Munemitsu, S., and Polakis, P. (1996). Binding of GSK3β to the APC-β-catenin complex and regulation of complex assembly. Science *272*, 1023–1026.

Rubinfeld, B., Albert, I., Porfiri, E., Munemitsu, S., and Polakis, P. (1997). Loss of beta-catenin regulation by the APC tumor suppressor protein correlates with loss of structure due to common somatic mutations of the gene. Cancer Res. *57*, 4624–4630.

Salic, A., Lee, E., Mayer, L., and Kirschner, M.W. (2000). Control of beta-catenin stability: reconstitution of the cytoplasmic steps of the wnt pathway in Xenopus egg extracts. Mol. Cell *5*, 523–532.

Sanchez-Vega, F., Mina, M., Armenia, J., Chatila, W.K., Luna, A., La, K.C., Dimitriadoy, S., Liu, D.L., Kantheti, H.S., Saghafinia, S., et al.; Cancer Genome Atlas Research Network (2018). Oncogenic signaling pathways in The Cancer Genome Atlas. Cell *173*, 321–337.e10.

Schaefer, K.N., and Peifer, M. (2019). Wnt/beta-catenin signaling regulation and a role for biomolecular condensates. Dev. Cell *48*, 429–444.

Schaefer, K.N., Bonello, T.T., Zhang, S., Williams, C.E., Roberts, D.M., McKay, D.J., and Peifer, M. (2018). Supramolecular assembly of the beta-catenin destruction complex and the effect of Wnt signaling on its localization, molecular size, and activity in vivo. PLoS Genet. *14*, e1007339.

Schaefer, K.N., Pronobis, M.I., Williams, C.E., Zhang, S., Bauer, L., Goldfarb, D., Yan, F., Major, M.B., and Peifer, M. (2020). Wnt regulation: exploring Axin-Disheveled interactions and defining mechanisms by which the SCF E3 ubiquitin ligase is recruited to the destruction complex. Mol. Biol. Cell *31*, 992–1014.

Schindelin, J., Arganda-Carreras, I., Frise, E., Kaynig, V., Longair, M., Pietzsch, T., Preibisch, S., Rueden, C., Saalfeld, S., Schmid, B., et al. (2012). Fiji: an open-source platform for biological-image analysis. Nat. Methods *9*, 676–682.

Schwarz-Romond, T., Metcalfe, C., and Bienz, M. (2007). Dynamic recruitment of axin by Dishevelled protein assemblies. J. Cell Sci. *120*, 2402–2412.

Sonn-Segev, A., Belacic, K., Bodrug, T., Young, G., VanderLinden, R.T., Schulman, B.A., Schimpf, J., Friedrich, T., Dip, P.V., Schwartz, T.U., et al. (2020). Quantifying the heterogeneity of macromolecular machines by mass photometry. Nat. Commun. *11*, 1772.

Spink, K.E., Polakis, P., and Weis, W.I. (2000). Structural basis of the Axin-adenomatous polyposis coli interaction. EMBO J. *19*, 2270–2279.

Stamos, J.L., and Weis, W.I. (2013). The β-catenin destruction complex. Cold Spring Harb. Perspect. Biol. *5*, a007898.

Steinhart, Z., and Angers, S. (2018). Wnt signaling in development and tissue homeostasis. Development *145*, dev146589.

Su, L.K., Vogelstein, B., and Kinzler, K.W. (1993). Association of the APC tumor suppressor protein with catenins. Science *262*, 1734–1737.

Su, Y., Fu, C., Ishikawa, S., Stella, A., Kojima, M., Shitoh, K., Schreiber, E.M., Day, B.W., and Liu, B. (2008). APC is essential for targeting phosphorylated beta-catenin to the SCFbeta-TrCP ubiquitin ligase. Mol. Cell *32*, 652–661.

Tacchelly-Benites, O., Wang, Z., Yang, E., Benchabane, H., Tian, A., Randall, M.P., and Ahmed, Y. (2018). Axin phosphorylation in both Wnt-off and Wnt-on states requires the tumor suppressor APC. PLoS Genet *14*, e1007178.

Tan, C.W., Gardiner, B.S., Hirokawa, Y., Layton, M.J., Smith, D.W., and Burgess, A.W. (2012). Wnt signalling pathway parameters for mammalian cells. PLoS ONE *7*, e31882.

Tauriello, D.V.F., and Maurice, M.M. (2010). The various roles of ubiquitin in Wnt pathway regulation. Cell Cycle *9*, 3700–3709.

UniProt Consortium (2021). UniProt: the universal protein knowledgebase in 2021. Nucleic Acids Res. *49* (D1), D480–D489.

van Kappel, E.C., and Maurice, M.M. (2017). Molecular regulation and pharmacological targeting of the β-catenin destruction complex. Br. J. Pharmacol. *174*, 4575–4588.

Voloshanenko, O., Erdmann, G., Dubash, T.D., Augustin, I., Metzig, M., Moffa, G., Hundsrucker, C., Kerr, G., Sandmann, T., Anchang, B., et al. (2013). Wnt secretion is required to maintain high levels of Wnt activity in colon cancer cells. Nat. Commun. *4*, 2610.

Wang, S., Yin, J., Chen, D., Nie, F., Song, X., Fei, C., Miao, H., Jing, C., Ma, W., Wang, L., et al. (2013). Small-molecule modulation of Wnt signaling via modulating the Axin-LRP5/6 interaction. Nat. Chem. Biol. *9*, 579–585.

Weissmann, F., Petzold, G., VanderLinden, R., Huis In 't Veld, P.J., Brown, N.G., Lampert, F., Westermann, S., Stark, H., Schulman, B.A., and Peters, J.-M. (2016). biGBac enables rapid gene assembly for the expression of large multisubunit protein complexes. Proc. Natl. Acad. Sci. U S A *113*, E2564–E2569.

Willert, K., Logan, C.Y., Arora, A., Fish, M., and Nusse, R. (1999). A Drosophila Axin homolog, Daxin, inhibits Wnt signaling. Development *126*, 4165–4173.

Winston, J.T., Strack, P., Beer-Romero, P., Chu, C.Y., Elledge, S.J., and Harper, J.W. (1999). The SCFbeta-TRCP-ubiquitin ligase complex associates specifically with phosphorylated destruction motifs in IkappaBalpha and beta-catenin and stimulates IkappaBalpha ubiquitination in vitro. Genes Dev. *13*, 270–283.

Wu, G., Xu, G., Schulman, B.A., Jeffrey, P.D., Harper, J.W., and Pavletich, N.P. (2003). Structure of a β-TrCP1-Skp1-β-catenin complex: destruction motif binding and lysine specificity of the SCF(β-TrCP1) ubiquitin ligase. Mol. Cell *11*, 1445–1456.

Xu, W., and Kimelman, D. (2007). Mechanistic insights from structural studies of beta-catenin and its binding partners. J. Cell Sci. *120*, 3337–3344.

Xue, B., Romero, P.R., Noutsou, M., Maurice, M.M., Rüdiger, S.G.D., William, A.M., Jr., Mizianty, M.J., Kurgan, L., Uversky, V.N., and Dunker, A.K. (2013). Stochastic machines as a colocalization mechanism for scaffold protein function. FEBS Lett. *587*, 1587–1591.

Yin, J., Straight, P.D., McLoughlin, S.M., Zhou, Z., Lin, A.J., Golan, D.E., Kelleher, N.L., Kolter, R., and Walsh, C.T. (2005). Genetically encoded short peptide tag for versatile protein labeling by Sfp phosphopantetheinyl transferase. Proc. Natl. Acad. Sci. U S A *102*, 15815–15820.

Yin, J., Lin, A.J., Golan, D.E., and Walsh, C.T. (2006). Site-specific protein labeling by Sfp phosphopantetheinyl transferase. Nat. Protoc. *1*, 280–285.

Young, G., Hundt, N., Cole, D., Fineberg, A., Andrecka, J., Tyler, A., Olerinyova, A., Ansari, A., Marklund, E.G., Collier, M.P., et al. (2018). Quantitative mass imaging of single biological macromolecules. Science *360*, 423–427.

Zhang, L., and Shay, J.W. (2017). Multiple roles of APC and its therapeutic implications in colorectal cancer. J. Natl. Cancer Inst. *109*, 55.

STAR★METHODS

KEY RESOURCES TABLE

REAGENT or RESOURCE	SOURCE	IDENTIFIER
Antibodies		
anti-AXIN1	Cell Signaling Technology	Cat# 2087; RRID: AB_2274550
anti-APC	Novus Biologicals	Cat# NB100-667; RRID: AB_521749
anti-β-catenin	Cell Signaling Technology	Cat# 610153; RRID: 397554
anti-phospho-β-catenin pS45	Cell Signaling Technology	Cat# 9564; RRID: AB_331150
anti-phospho-β-catenin pS33/pS37/pT41	Cell Signaling Technology	Cat# 9561; RRID: AB_331729
anti-non-phospho-β-catenin S33/S37/T41	Cell Signaling Technology	Cat# 8814; RRID: AB_11127203
anti-GSK3β	Cell Signaling Technology	Cat# 12456; RRID: AB_2636978
anti-GSK3β pS9	Cell Signaling Technology	Cat# 5558; RRID: AB_10013750
anti-StrepMAB-Classic	IBA Life Sciences	Cat# 2-1507; RRID: AB_513133
anti-ubiquitin P4D1 clone	Enzo Life Sciences	Cat# BML-PW0930; RRID: AB_10998070
anti-ubiquitin K48 linkage-specific	Abcam	Cat# ab140601; RRID: AB_2783797
anti-ubiquitin K63 linkage-specific	Abcam	Cat# ab179434; RRID: AB_2737584
IRDye 680RD donkey anti-mouse	LI-COR	Cat# 926-68072; RRID: AB_10953628
IRDye 800CW donkey anti-rabbit	LI-COR	Cat# 926-32213; RRID: AB_621848
IRDye 800CW donkey anti-mouse	LI-COR	Cat# 926-32212; RRID: AB_621847
Bacterial and virus strains		
E. coli BL21-CodonPlus(DE3)-RIL	Agilent	Cat# 230245
E. coli DH10 MultiBacTurbo	ATG:biosynthetics GmbH (Bieniossek et al., 2008)	N/A
Chemicals, peptides, and recombinant proteins		
Protease inhibitor cocktail tablets	Thermo Scientific	Cat# A32965
AEBSF	Melford Laboratories Ltd	Cat# A20010
β-Glycerophosphate	Sigma-Aldrich	Cat# G9422
StrepTactin-Sepharose resin	IBA Life Sciences	Cat# 2-1201
Cellfectin II	Thermo Scientific	Cat# 11496015
Penicillin-Streptomycin	Thermo Scientific	Cat# 15070063
Alexa Fluor® 680 C_2 Maleimide	Thermo Scientific	Cat# A20344
Coenzyme A	Sigma-Aldrich	Cat# C3019
Sfp1	Dr Alessandro Vannini lab	N/A
Lambda phosphatase	Dr Gideon Coster lab	N/A
APPB1-Uba3	Enzo Life Sciences	Cat# PRT112
UbcH12	Enzo Life Sciences	Cat# UW9145
His_6-NEDD8	Enzo Life Sciences	Cat# UW9225
N-terminally fluorescein-labeled ubiquitin	Boston Biochem.	Cat# U-580
ubiquitin	Boston Biochem.	Cat# U-100H
K48-linked ubiquitin chains	R&D Systems	Cat# UC-230
K63-linked ubiquitin chains	R&D Systems	Cat# U-C-330
UBA1	Dr Edward Morris lab	N/A
GSK3 inhibitor CHIR-99021	Sigma-Aldrich	Cat# 361571
CK1 inhibitor D4476	Sigma-Aldrich	Cat# 218696
ATP regeneration solution	Enzo Lifesciences	Cat# BML-EW9810
26S proteasome	Boston Biochem.	Cat# e-365
Proteasome inhibitor MG132	Sigma-Aldrich	Cat# M7449
γ-^{32}P-ATP	Hartmann Analytics	Cat# SCP-301

(*Continued on next page*)

Continued		
REAGENT or RESOURCE	SOURCE	IDENTIFIER
Pierce^{TM} silver stain kit	Thermo Scientific	Cat# 24612
SYPRO Ruby protein stain	Thermo Scientific	Cat# S12000
InstantBlue protein stain	Abcam	Cat# 119211
NativeMark^{TM} unstained protein standard	Thermo Scientific	Cat# LC0725
Precision Plus Protein^{TM} standards	Bio-Rad	Cat:# 1610373
Experimental models: Cell lines		
S. frugiperda Sf9 insect cells	Thermo Scientific	Cat# 11496015
T. ni High-Five insect cells	Thermo Scientific	Cat# B85502
Recombinant DNA		
pLIB vector	Jan-Michael Peters lab, IMP (Weissmann et al., 2016)	N/A
pBIG1a vector	Jan-Michael Peters lab, IMP (Weissmann et al., 2016)	N/A
pBIG1b vector	Jan-Michael Peters lab, IMP (Weissmann et al., 2016)	N/A
pBIG2ab vector	Jan-Michael Peters lab, IMP (Weissmann et al., 2016)	N/A
pLIB_ APC	GenScript (this paper)	N/A
pLIB_ APC T1556*	This paper, see Table S10	N/A
pLIB_ APC Q1338*	This paper, see Table S10	N/A
pLIB_ APC S811*	This paper, see Table S10	N/A
pLIB_dStrepII-TEV-APC	GenScript (this paper)	N/A
pLIB_dStrepII-TEV-APC SAMP mutant	GenScript (this paper, see Table S10)	N/A
pLIB_dStrepII-TEV-APC 20R phospho mutant	GenScript (this paper, see Table S10)	N/A
pLIB_dStrepII-TEV-APC ΔCID	This paper, see Table S10	N/A
pLIB_dStrepII-TEV-APC T1556*	This paper	N/A
pLIB_dStrepII-TEV-APC Q1338*	This paper	N/A
pLIB_dStrepII-TEV-APC S811*	This paper	N/A
pBIG1b_APC	This paper	N/A
pBIG1b_APC T1556*	This paper	N/A
pBIG1b_APC Q1338*	This paper	N/A
pBIG1b_APC S811*	This paper	N/A
pLIB_AXIN1-TEV-dStrepII	GenScript (this paper)	N/A
pLIB_AXIN1 M3-TEV-dStrepII	This paper, see Table S10	N/A
pLIB_AXIN1	This paper	N/A
pLIB_AXIN1 M3	This paper, see Table S10	N/A
pLIB_dStrepII-TEV-β-catenin	GenScript (this paper)	N/A
pLIB_dStrepII-TEV-β-catenin-ybbR	This paper, see Table S10	N/A
pLIB_dStrepII-TEV-β-catenin S33A, S37A, T41A	This paper, see Table S10	N/A
pLIB_β-catenin	This paper	N/A
pLIB_CK1α	GenScript (this paper)	N/A
pLIB_GSK3β	GenScript (this paper)	N/A
pLIB_GSK3β K85N, D181N	This paper, see Table S10	N/A
pLIB_β-TrCP	GenScript (this paper)	N/A
pLIB_CUL1	GenScript (this paper)	N/A
pLIB_SKP1	GenScript (this paper)	N/A
pLIB_RBX1	GenScript (this paper)	N/A
pBIG1a_AXIN1-kinase complex	This paper, see Table S11	N/A

(*Continued on next page*)

Continued		
REAGENT or RESOURCE	SOURCE	IDENTIFIER
pBIG1a_AXIN1-kinase GSK3β-dead complex	This paper, see Table S11	N/A
pBIG1a_AXIN1 M3-kinase complex	This paper, see Table S11	N/A
pBIG1a_AXIN1-kinase-β-catenin complex	This paper, see Table S11	N/A
pBIG2ab_wild-type DC (destruction complex)	This paper, see Table S11	N/A
pBIG2ab_DC_APC T1556* complex	This paper, see Table S11	N/A
pBIG2ab_DC_APC Q1338* complex	This paper, see Table S11	N/A
pBIG2ab_DC_APC S811* complex	This paper, see Table S11	N/A
pBIG2ab_DC_AXIN1 M3 complex	This paper, see Table S11	N/A
pBIG2ab_SCF$^{\beta\text{-TrCP}}$ E3 ligase	This paper, see Table S11	N/A
pProEx HIS_6-TEV-UBCH5c	Frank Sicheri lab (Chou et al., 2012)	N/A
Software, algorithms, databases		
ImageJ (Fiji)	NIH	RRID: SCR_002285
ImageJ Linearize GelData plugin	https://rsb.info.nih.gov/ij/plugins/linearize-gel-data.html	N/A
GraphPad Prism	GraphPad	RRID: SCR_002798
Image Studio Lite	LI-COR	RRID: SCR_013715
ImageQuant	GE Healthcare	RRID: SCR_04246
ASTRA version 7.3.1	Wyatt Technology	RRID: SCR_016255
Acquire^{MP} acquisition software	Refeyn Ltd	Cat# 600-1
Discover^{MP} analysis software	Refeyn Ltd	Cat# 600-1
Scientific Python Development Environment	https://www.spyder-ide.org/	RRID: SCR_008394
DC stoichiometry algorithm	This paper	N/A
ExPASy ProtParam	https://web.expasy.org/protparam/ (Gasteiger et al., 2005)	RRID: SCR_018087
UniProt	https://www.uniprot.org (UniProt Consortium, 2021)	RRID:SCR_004426
Research Collaboratory for Structural Bioinformatics Protein Data Bank (RCSB PDB)	http://www.rcsb.org/pdb/ (Berman et al., 2002)<br>PDB: 1EMU (Spink et al., 2000)<br>PDB: 1V18 (Ha et al., 2004)<br>PDB: 1H8F (Dajani et al., 2001)	RRID:SCR_012820
Deposited data		
Original data and gel/Western blot images	This paper; Mendeley Data	https://doi.org/10.17632/wynmtr788p.1
Other		
Insect-Xpress cell medium	Lonza	Cat# BELN12-730Q
NuPAGE™ 4 – 12% Bis-Tris SDS-PAGE gels	Thermo Scientific	Cat# NP0321; Cat# WG1402; Cat# WG1403A
StrepTrap HP column	GE Healthcare	Cat# 28907547
HiLoad 16/60 Superdex 200 column	GE Healthcare	Cat# 28989335
HiLoad 16/600 Superose 6 column	GE Healthcare	Cat# 29323952
WTC-50N5 column	Wyatt Technology	Cat# WTC-50N5
WTC-100N5 column	Wyatt Technology	Cat# WTC-100N5
DAWN® MALS detector	Wyatt Technology	Cat# N1018; RRID: SCR_020896
Optilab refractometer	Wyatt Technology	Cat# N1001OP
Agilent 1260 Infinity II HPLC system	Agilent	RRID: SCR_019354
Odyssey CLx fluorescence imager	LI-COR	RRID: SCR_014579
Typhoon FLA 9500	GE Healthcare	RRID: SCR_019957
High Precision Coverslips	VWR	Cat# 630-2187
CultureWell™ gaskets	Sigma-Aldrich	Cat# GBL103250
Refeyn One^{MP} mass photometer	Refeyn Ltd	Cat# 100-01

CellPress
OPEN ACCESS

RESOURCE AVAILABILITY

Lead contact

Further information and requests for resources and reagents should be directed to and will be fulfilled by the Lead Contact, Sebastian Guettler (sebastian.guettler@icr.ac.uk).

Materials availability

Reagents generated in this study will be made available upon request, but we may require a completed Materials Transfer Agreement if there is potential for commercial application.

Data and code availability

- Original data and gel/Western blot images have been deposited at Mendeley and are publicly available as of the date of publication. The DOI is listed in the Key resources table.
- All original code is available in this paper's supplemental information (Data S1).
- Any additional information required to reanalyse the data reported in this paper is available from the lead contact upon request.

EXPERIMENTAL MODEL AND SUBJECT DETAILS

The E1 protein, λ-phosphatase and Sfp enzyme (all obtained as gifts; see below) were produced in *Escherichia coli*. The E2 protein was expressed in *Escherichia coli* BL21-CodonPlus(DE3)-RIL cells. All other proteins and protein complexes were expressed in either Sf9 (*Spodoptera frugiperda*) or High-Five (*Trichoplusia ni*) insect cells.

METHOD DETAILS

Cloning and expression of recombinant proteins and complexes

cDNA constructs of DC components and the $SCF^{\beta\text{-}TrCP}$ E3 ligase complex were codon-optimized for *E.coli* expression by GenScript and were subcloned into the pLIB vector (Weissmann et al., 2016) under control of the polyhedron gene promoter of the *Autographa californica* nuclear polyhedrosis virus (AcNPV). The codon-optimized DC cDNAs were inserted into the pLIB vector as follows:

BamHI - Kozak sequence (AGCCGCCACC) - Start codon - insert - Stop codon - HindIII

Affinity-tagged versions were synthesized by extending the ORF with a double-StrepII (dStrepII) tag followed (for N-terminal tags) or preceded (for C-terminal tags) by a TEV protease site. Tag and TEV site sequences were as follows:

N-terminal: MSA-WSHPQFEK-$(GGGS)_2$-GGSA-WSHPQFEK-GA-ENLYFQG

C-terminal: GA-ENLYFQG-SA-WSHPQFEK-$(GGGS)_2$-GGSA-WSHPQFEK *stop codon.

See Table S10 for plasmid details. Multi-protein expression constructs were assembled using the biGBac system (Weissmann et al., 2016) to generate single baculoviruses with core destruction complex (DC) variants, sub-complexes and the $SCF^{\beta\text{-}TrCP}$ E3 ubiquitin ligase complex (Table S11). Correct assembly was verified by restriction digest and sequencing of the open reading frames. Further construct details are available upon request.

Viral bacmids for the expression of individual subunits (from the pLIB vector) or complexes (from the pBIG vector) were generated using Tn7 transposition in DH10 MultiBacTurbo *E. coli* competent cells (Bieniossek et al., 2008). Insect cells were grown in Insect-Express media (# BELN12-730Q, Lonza) supplemented with 1% Penicillin-Streptomycin (# 15070063, Thermo Scientific). 2 mL of 5×10^5 cells/mL adherent Sf9 cells (# 11496015, Thermo Scientific) were transfected with purified bacmids using Cellfectin II reagent (# 10362100, Thermo Scientific) to generate recombinant baculoviruses. P1 virus was harvested after 72 h of incubation without shaking at 27°C. All of the ≈2 mL of harvested media was used to infect a fresh 30-mL culture of Sf9 cells (5×10^5 cells/mL) for a second round of viral amplification at 27°C and shaking at 130 rpm. Virus-containing media were harvested (P2 virus) once cell viability had dropped to ≈80%, and stored at 4°C.

Destruction (sub-) complexes were expressed in 0.25-L cultures of Sf9 cells, whereas the $SCF^{\beta\text{-}TrCP}$ E3 ubiquitin ligase complex and individual DC components in 0.5-L cultures of High-Five cells (# B85502, Thermo Scientific). The amplified viruses were used to infect 1×10^6 cells/mL of Sf9 or High-Five cells and incubated at 27°C, 130 rpm. Cultures were harvested by centrifugation (250 x g for 10 min at 4°C) when cell viability had reached between 90 – 80%, which typically took approximately 72 h, and cell pellets were stored at −80°C.

APC purification

High-Five cell pellets were resuspended in a buffer containing 50 mM HEPES-NaOH pH 7.5, 750 mM NaCl, 10 mM β-mercaptoethanol, 5% glycerol, 1 mM AEBSF protease inhibitor and Pierce protease inhibitor tablets, EDTA-free (# A32965, Thermo Scientific). Lysates were briefly sonicated, centrifuged at 45,000 x g for 45 min to remove insoluble material and filtered through a 0.45-μm filter. The cleared lysate was loaded onto a 5-mL StrepTrap HP column (GE Healthcare) and washed with 20 column volumes (CV) of wash buffer (identical to lysis buffer but lacking protease inhibitors). dStrepII-fusion proteins were eluted with 5 mM *d*-desthiobiotin in wash

buffer. The eluted peak fractions were pooled and dialysed overnight at 4°C against 50 mM HEPES-NaOH pH 7.5, 500 mM NaCl, 2 mM TCEP, 5% glycerol and His_6-Strep-TEV protease to cleave the dStrepII affinity tag. Residual affinity tag, uncleaved protein and His_6-Strep-TEV protease were removed through a subtractive Strep affinity chromatography step using a 5-mL StrepTrap HP column. The cleaved APC proteins were concentrated and flash-frozen in liquid nitrogen.

β-catenin purification

High-Five cell pellets were resuspended in a buffer containing 50 mM HEPES-NaOH pH 7.5, 500 mM NaCl, 10 mM β-mercaptoethanol and Pierce protease inhibitor tablets, EDTA-free. The subsequent steps until after loading of the cleared lysate onto the 5-mL StrepTrap column were performed as described for the purification of APC (see section above). Unbound material was removed with 20 CV of wash buffer, followed by equilibration with 2 CV of wash buffer containing 2 mM $MnCl_2$. Purified λ-phosphatase (kind gift from Dr Jerome Gouge and Dr Gideon Coster) at 20 μg/mL in 3 CV wash buffer containing 2 mM $MnCl_2$ was passed through the StrepTrap column. The column was capped and incubated at room temperature for 30 min to de-phosphorylate β-catenin. Lambda phosphatase was removed with 20 CV of wash buffer (without $MnCl_2$) and the de-phosphorylated β-catenin eluted with 5 mM *d*-desthiobiotin. The eluted peak fractions were pooled and dialysed overnight at 4°C against 50 mM HEPES-NaOH pH 7.5, 150 mM NaCl, 2 mM TCEP and His_6-StrepII-tagged TEV protease to cleave the dStrepII affinity tag. Residual affinity tag, uncleaved protein and His_6-Strep-TEV protease were removed through a subtractive Strep affinity chromatography step using a 5-mL StrepTrap HP column.

β-catenin-ybbR Alexa Fluor® 680 labeling

A CoA-fluorophore conjugate can be covalently attached to a serine residue in a short ybbR peptide by the *Bacillus subtilis* Sfp phosphopantetheinyl transferase (Yin et al., 2005). Purified Sfp enzyme was kindly provided by Dr Erin Cutts, and the labeling of β-catenin-ybbR was performed as described (Yin et al., 2006) or with the following modifications. Alexa Fluor® 680 maleimide (# A20344, Thermo Scientific) at 2.5 mM was conjugated to 0.5 mM Coenzyme A (# C3019, Sigma-Aldrich) in 100 mM sodium phosphate pH 7.0 reaction buffer, with five-fold excess of fluorophore to ensure near-complete conjugation of CoA. The reaction was incubated in the dark for 2 h at room temperature, and unconjugated Alexa Fluor® 680 maleimide was quenched with 12.5 mM DTT and incubation for a further 15 min. The entire sample was used in the following β-catenin labeling reaction: CoA conjugation reaction with ≈0.5 mM Alexa Fluor® 680-CoA, 26 μM β-catenin-ybbR, 6 μM Sfp enzyme and 10 mM $MgCl_2$. The reaction was performed in the dark for 1.5 h at room temperature, and reaction components were separated from labeled β-catenin-Alexa Fluor® 680 by size exclusion chromatography on a HiLoad 16/60 Superdex 200 column (prep grade, GE Healthcare) equilibrated in 50 mM HEPES-NaOH pH 7.5, 150 mM NaCl, 2 mM TCEP, 5% glycerol. Pure protein fractions were pooled, concentrated and flash-frozen in liquid nitrogen.

Purification of the destruction complex and subcomplexes

Sf9 cell pellets were resuspended in 50 mM HEPES-NaOH pH 7.5, 150 mM NaCl, 10 mM β-mercaptoethanol, 5% glycerol, 10 mM β-glycerophosphate, 1 mM AEBSF and Pierce protease inhibitor tablets, EDTA-free. Lysates were briefly sonicated and centrifuged at 25,000 x g for 45 min to remove insoluble material. Cleared lysates were incubated in batch with 1 – 2 mL of pre-equilibrated Strep-Tactin Sepharose resin (IBA Lifesciences) at 4°C rotating for 2 h. Resin was washed with > 50 CV of wash buffer (lysis buffer lacking protease and phosphatase inhibitors), and protein complexes were eluted with wash buffer supplemented with 50 mM *d*-biotin. Attempts to concentrate the purified destruction (sub-) complexes resulted in their precipitation. To obtain more concentrated samples, the protein complexes were eluted with biotin, which competes with the dStrepII tag more efficiently than desthiobiotin on the Strep-Tactin resin. The eluted peak fractions were pooled and dialysed overnight at 4°C against 50 mM HEPES-NaOH pH 7.5, 150 mM NaCl, 2 mM TCEP, 5% glycerol, then flash-frozen in liquid nitrogen.

$SCF^{\beta\text{-TrCP}}$ E3 ligase complex purification and in-vitro neddylation

High-Five cell pellets were resuspended in a buffer containing 50 mM HEPES-NaOH pH 7.5, 200 mM NaCl, 10 mM β-mercaptoethanol, 0.1% NP-40, 5% glycerol and Pierce protease inhibitor tablets, EDTA-free. Lysates were briefly sonicated, centrifuged at 45,000 x g for 45 min to remove insoluble material and filtered through a 0.45-μm filter. The cleared lysate was loaded onto a 5-mL StrepTrap HP column (GE Healthcare), washed with 20 CV of wash buffer (lysis buffer lacking NP-40 and protease inhibitors), and the protein complex was eluted with 5 mM *d*-desthiobiotin. The peak protein fractions were pooled and subjected to size exclusion chromatography on a HiLoad 16/600 Superose 6 column (prep grade, GE Healthcare), equilibrated in 50 mM HEPES-NaOH pH 7.5, 200 mM NaCl, 2 mM TCEP, 5% glycerol. The elution fractions containing all the components of the $SCF^{\beta\text{-TrCP}}$ E3 ligase complex were pooled, concentrated and flash-frozen in liquid nitrogen. Elution fractions from a second peak corresponding to a complex without β-TrCP were processed likewise.

The *in-vitro* neddylation was performed in the following reaction: 8 μM $SCF^{\beta\text{-TrCP}}$ E3 ligase complex, 350 nM APPBP1-Uba3 (# PRT112, Enzo Lifesciences), 1.8 μM UbcH12 (# UW9145, Enzo Lifesciences), 10 μM His_6-NEDD8 (# UW9225, Enzo Lifesciences), 10 mM $MgCl_2$ and 1.25 mM ATP. The reaction was incubated for 10 min at 37°C and stopped by the addition of DTT at a final concentration of 15 mM. A Strep-affinity chromatography on a 5-mL StrepTrap HP column was performed to remove the reaction components from the neddylated E3 ligase complex. The bound material was washed with 20 CV of wash buffer (50 mM HEPES-NaOH

pH 7.5, 200 mM NaCl, 2 mM TCEP, 5% glycerol) and eluted with 5 mM *d*-desthiobiotion. Elution fractions containing neddylated $SCF^{\beta\text{-TrCP}}$ E3 ligase were pooled, concentrated and flash-frozen in liquid nitrogen.

E1 and E2 purifications

Purified recombinant human E1 enzyme (UBA1, ubiquitin-like modifier-activating enzyme 1), expressed in *E. coli*, was generously provided by Dr Aylin Morris-Davies and Dr Edward Morris. The bacterial expression plasmid for the human E2 ubiquitin-conjugating enzyme (pProEx His_6-TEV- UBCH5c) was provided by Dr Frank Sicheri (Chou et al., 2012). Human UBCH5c was expressed as His_6-TEV fusion protein in *E. coli* BL21-CodonPlus(DE3)-RIL (# 230245, Agilent) grown in 2 L of LB media. Expression was induced at an OD_{600} of 0.6 with 1 mM IPTG overnight at 18°C. Cells were harvested by centrifugation, resuspended in a buffer containing 50 mM HEPES-NaOH pH 7.5, 150 mM NaCl, 5 mM imidazole, 10 mM β-mercaptoethanol, EDTA-free Pierce protease inhibitor tablets, lysed by sonication and centrifuged at 45,000 x g for 45 min to remove insoluble material. Lysates were filtered through a 0.45-μm filter and loaded onto a 5-mL HisTrap HP affinity column (GE Healthcare). The bound material was washed with 20 CV of wash buffer (lysis buffer lacking protease inhibitors). His_6-TEV-UBCH5c was eluted with a linear imidazole gradient (5 to 250 mM imidazole) in a buffer containing 50 mM HEPES-NaOH 7.5, 150 mM NaCl and 5 mM β-mercaptoethanol. The peak fractions containing relatively pure protein were pooled and dialysed overnight at 4°C against 50 mM HEPES-NaOH pH 7.5, 150 mM NaCl and 2 mM TCEP, concentrated and flash-frozen in liquid nitrogen.

SEC-MALS

Affinity-purified DCs (wild-type and a mutant variant containing polymerization-deficient AXIN1 M3 (Fiedler et al., 2011)) were quantified by UV spectroscopy at 280 nm (A_{280}). Given the unknown stoichiometry of the complexes, an accurate determination of their concentrations was not possible as extinction coefficients could not be reliably estimated. Therefore, serial dilutions of the complexes were made on the basis of A_{280}. 90 μL samples of different dilutions were resolved by size exclusion chromatography on an Agilent 1260 Infinity II HPLC system with a WTC-100N5 column (Wyatt Technology). The separation was performed in 50 mM HEPES-NaOH pH 7.5, 150 mM NaCl, 2 mM TCEP, 5% glycerol at a flow rate of 0.25 mL/min with collection of 100 μL elution fractions. 25 μL of elution fractions and 15 μL of the DC complexes, representing one sixth of input material, were analyzed by SDS-PAGE. Light scattering and differential refractive index (dRI) of the samples were analyzed in-line using the DAWN® and Optilab instruments from Wyatt Technologies, respectively. Data analysis, using the Zimm light scattering model and a dn/dc of 0.185 mL/g, was performed using Wyatt's ASTRA software version 7.3.1. Weight average molecular weights (M_w), number average molecular weights (M_n) and dispersities (Đ) with their associated uncertainties for the elution peak areas were obtained through the ASTRA software data analysis (see ASTRA 7 User's Guide for details). See the "*In-silico* stoichiometry modelling of the DC and estimation of DC molar concentrations" section below for details on estimating DC concentrations in input samples and eluates.

Assessment of $SCF^{\beta\text{-TrCP}}$ E3 ligase interactions with DC components

Size exclusion chromatography of affinity-purified $SCF^{\beta\text{-TrCP}}$ E3 ligase (as described above) resulted in the separation of fully assembled $SCF^{\beta\text{-TrCP}}$ E3 ligase and a distinct subpopulation of SCF E3 ligase lacking β-TrCP ($SCF^{w/o\ \beta\text{-TrCP}}$ E3 ligase). All subsequent binding analyses were performed in buffer containing 50 mM HEPES-NaOH pH 7.5, 200 mM NaCl, 2 mM TCEP. Purified DC components APC, AXIN1 WT or β-catenin at 1.5 μM were incubated with 1.5 μM of either $SCF^{\beta\text{-TrCP}}$ E3 ligase or $SCF^{w/o\ \beta\text{-TrCP}}$ E3 ligase for 30 min on ice. For each individual set of samples, i.e., APC - $SCF^{\beta\text{-TrCP}}$ E3 ligase and APC - $SCF^{w/o\ \beta\text{-TrCP}}$ E3 ligase, the individual components were also incubated with equal volumes of the respective protein buffer instead of the component being omitted. As a positive control, 1.5 μM β-catenin was phosphorylated using 75 nM AXIN1-CK1α-GSK3β complex in the presence of 5 mM $MgCl_2$ and 1 mM ATP, by incubating at 25°C for 60 min and terminating the reaction by addition of 10 mM EDTA. A similar reaction lacking ATP was conducted in parallel as a negative (non-phosphorylated β-catenin) control.

90 μL of the samples were resolved by size exclusion chromatography on an Agilent 1260 Infinity II HPLC system with a WTC-50N5 column (Wyatt Technology). The setup, including detectors and fraction collector, was the same as for the SEC-MALS experiments described above. The separation was performed in 50 mM HEPES-NaOH pH 7.5, 200 mM NaCl, 2 mM TCEP at a flow rate of 0.25 mL/min with collection of 100 μL elution fractions. 10 μL of elution fractions were resolved by SDS-PAGE (4 – 12% gradient Bis-Tris polyacrylamide gels (Life Technologies)), and proteins were detected by silver staining using the Pierce™ Silver stain kit. Separately, one sixth of input materials (15 μL) was analyzed by SDS-PAGE and Coomassie staining. Phosphorylation of β-catenin was detected through immunoblot analysis of the input samples and elution fractions using anti-β-catenin and anti-phospho-β-catenin pS33, pS37, pT41 antibodies.

β-catenin phosphorylation and ubiquitylation reactions

The concentrations of proteins added to the reactions were determined by UV spectroscopy (A_{280}) using theoretical extinction coefficients calculated using the ProtParam tool in ExPASy (Gasteiger et al., 2005) for the fully reduced forms of the proteins. For the AXIN1-kinase complex, we assumed a 1:1:1 AXIN1:CK1α:GSK3β stoichiometry to calculate the theoretical extinction coefficient. All reactions were performed in the following reaction buffer: 50 mM sodium phosphate pH 7.0, 150 mM NaCl, 1 mM TCEP. Salt contributions from the proteins added to the reactions were less than 50 mM NaCl. Master mixes were made of reaction components whenever possible to facilitate reaction assembly and to minimize variability between individual reactions. Complete reactions of

30 µL were prepared on ice from three components; the concentrations listed refer to the final concentrations in the 30 µL reactions: **I.** Ubiquitin mix (10 µL per reaction): reaction buffer containing 10 µM recombinant human ubiquitin (# U-100H, Boston Biochem.), 1 µM N-terminally fluorescein-labeled human ubiquitin (# U-580, Boston Biochem.), 50 nM E1 (human UBA1), 1 µM E2 (UBCH5c), 10 mM $MgCl_2$, 5 mM ATP added just before starting the reactions. **II.** DC components (10 µL per reaction): reaction buffer lacking NaCl (due to the high NaCl contribution from the APC protein stocks) containing 1 µM β-catenin-Alexa Fluor® 680, 200 nM APC, 150 nM AXIN1-kinase complex. Equal volumes of different APC proteins and AXIN1-kinase complex variants were added to reactions, respectively, by diluting the more highly concentrated stocks such that the stocks had the same concentrations as that which was least concentrated among them. Dilution ensured equal salt carryover between different reactions. In addition, equal volumes of corresponding protein buffers were used when proteins were omitted from the reaction. GSK3 inhibitor CHIR-99021 (# 361571, Sigma-Aldrich) or CK1 inhibitor D4476 (# 218696, Sigma-Aldrich) were added at this step, at the final concentrations stated in the figure legends; vehicle solution was used in control reactions. **III.** E3 ligase complex (10 µL per reaction): 100 nM $SCF^{\beta\text{-TrCP}}$ E3 ligase complex in reaction buffer. The reaction parts II and III were combined and incubated at room temperature for 10 min. Likewise, reaction part I was also prepared, but without ATP, and incubated at room temperature for 10 min. The reaction was started by adding ATP to reaction part I and combining it with II and III. Reactions were incubated under agitation (500 rpm) at 25°C in a Thermomixer (Eppendorf) and terminated at the specified time points by adding 9 µL of the progressing reaction mixture to 9 µL of 2x SDS-PAGE loading buffer and heating to 95°C for 5 min. Typically, 7 µL of boiled samples were resolved on 4 – 12% gradient Bis-Tris polyacrylamide gels (Life Technologies) and imaged for β-catenin-Alexa Fluor® 680, fluorescein-ubiquitin and the protein standards on a Typhoon FLA 9500 biomolecular imager (GE Healthcare). Signals were quantified using ImageQuant software (GE Healthcare). The protein standards (# 1610373, Bio-Rad) were imaged at 635 nm excitation wavelength, which to a lesser extent also triggers Alexa Fluor® 680 fluorescence. The raw image contained β-catenin-Alexa Fluor® 680 signals obtained at 635 nm and 685 nm excitation wavelengths. The β-catenin-Alexa Fluor® 680 signals obtained at 635 nm excitation wavelength were masked, leaving only the lane showing the protein standards. The β-catenin-Alexa Fluor® 680, fluorescein-ubiquitin and the protein standards images were merged back to obtain the final multicolor images presented. Where downstream immunoblot analyses were performed, the same gels were subsequently used to transfer the proteins onto nitrocellulose membranes. These were scanned for β-catenin-Alexa Fluor® 680 on the Typhoon imager before being processed for immunoblot detection and analyzed on an Odyssey infrared imaging system (LI-COR).

In-vitro phosphorylation and de-phosphorylation

The reactions were set up with 40 µL of affinity-purified DCs (A_{280} of 0.4) in a final reaction volume of 45 µL. For the phosphorylation reactions, affinity-purified DCs were incubated with 10 mM $MgCl_2$, 5 mM ATP for 30 min at 25°C. Reactions were terminated by adding 15 µL of 4x SDS-PAGE loading buffer, followed by heating to 95°C for 5 min. Where indicated, affinity-purified DCs were de-phosphorylated in a reaction containing 2 mM $MnCl_2$ and 2 µM λ-phosphatase for 30 min at 25°C and reactions terminated by adding 15 µL of 4x SDS-PAGE loading buffer, followed by heating to 95°C for 5 min.

β-catenin proteasomal degradation

In-vitro β-catenin ubiquitylation reactions of 30 µL were set up as described above (β-catenin phosphorylation and ubiquitylation reactions) in the presence of 5 µM GSK3 inhibitor and incubated for 60 min under agitation (500 rpm) at 25°C. Immediately afterward, reactions were placed on ice. 5 µL of each reaction were mixed with 5 µL of 2x SDS-PAGE loading buffer and heated to 95°C for 5 min; these samples served as input references for the subsequent proteasome reactions. The β-catenin proteasomal degradation was performed in the following buffer: 50 mM Tris-HCl pH 7.5, 100 mM NaCl, 2 mM ATP, 5 mM $MgCl_2$, 1 mM TCEP, 0.2 mg/mL BSA, 2% glycerol supplemented with 1x ATP regeneration solution (# BML-EW9810, Enzo Lifesciences) and 25 nM human 26S proteasome (# e-365, Boston Biochem). Control reactions were further supplemented with 10 µM MG132 (# M7449, Sigma-Aldrich) in the presence or absence of 26S proteasome. 5 µL of each ubiquitylation reaction were distributed over four microcentrifuge tubes and incubated in a total reaction volume of 15 µL under agitation (500 rpm) at 25°C. The reactions were stopped after 1, 2 and 3 h by adding 5 µL of 4x SDS-PAGE loading buffer and heating to 95°C for 5 min. The samples were resolved on 4 – 12% gradient Bis-Tris polyacrylamide gels (Life Technologies) and imaged for β-catenin-Alexa Fluor ® 680 and fluorescein-ubiquitin on a Typhoon FLA 9500 biomolecular imager (GE Healthcare). Signals were quantified using ImageQuant software (GE Healthcare).

Radiometric kinetic kinase assays

All reactions were performed in the following reaction buffer: 50 mM HEPES-NaOH pH 7.0, 150 mM NaCl, 10 mM $MgCl_2$, 1 mM TCEP at room temperature. Reactions were initiated by adding 200 µM unlabelled ATP with 0.9 µCi γ-^{32}P-ATP (# SCP-301, Hartmann Analytics) or serial dilutions of this ATP mix. Master mixes were made of reaction components whenever possible to facilitate reaction assembly. Assays were set up in a 96-well PCR plate using a multichannel pipette to ensure simultaneous initiation and termination of parallel reactions. Each 30 µL reaction in either an ATP titration or β-catenin titration experiment was set up from two components as specified below; the concentrations listed refer to the final concentrations in the 30 µL reactions:

ATP titration reactions

I. ATP mixture (6 µL per reaction): ATP buffer (50 mM HEPES-NaOH pH 7.0, 150 mM NaCl, 1 mM TCEP) containing 200 µM unlabelled ATP and 0.9 µCi γ-^{32}P-ATP. A two-fold serial dilution of a 200-µM ATP master mix was made in ATP buffer with each dilution volume sufficient for at least 2.5 reactions; ATP concentrations were 200, 100, 50, 12.5, 6.26, 3.125, 1.56, 0.78 µM.

II. DC components mixture (24 μL per reaction): reaction buffer lacking NaCl (due to the high NaCl contribution from the APC protein) containing 1 μM β-catenin, 200 nM APC (or an equal volume of APC protein buffer added to reactions without APC), 150 nM AXIN1-kinase complex, 10 mM $MgCl_2$ and incubated for 10 min at room temperature.

6 μL of ATP mixture were placed in individual wells on a 96-well PCR plate; a separate set of wells contained 29 μL of the DC components mixture.

β-catenin titration reactions

I. ATP mixture (6 μL per reaction): ATP buffer (50 mM HEPES-NaOH pH 7.0, 150 mM NaCl, 1 mM TCEP) containing 200 μM unlabelled ATP and 0.9 μCi γ-^{32}P-ATP.

II. DC components mixture (24 μL per reaction): reaction buffer lacking NaCl (due to the high NaCl contribution from the APC protein) and containing 200 nM APC (or an equal volume of APC protein buffer added to reactions without APC), 150 nM AXIN1-kinase complex, 10 mM $MgCl_2$ and serial dilutions of β-catenin (15, 10, 7.5, 5, 2.5, 1.0, 0.5, 0.25 μM). A master mix of the reaction components without β-catenin was placed in 96-well PCR plates, each well containing solution sufficient for 1.25 reactions. Subsequently, β-catenin from serial dilutions was mixed into the same wells, completing the DC component mixture.

6 μL of ATP mixture were placed in individual wells on a 96-well PCR plate.

To start the reactions, 24 μL of the DC components mixture was transferred into the wells containing the ATP mixture using a multichannel pipette and mixed by pipetting 3 times before starting the time course. Using a multichannel pipette, the reactions were terminated at specific time points (0.5, 1, 2 and 5 min) by pipetting 6 μL of the reaction volume into wells containing 6 μL of 2x SDS-PAGE loading buffer. At the end of the experiment, the samples were transferred to microcentrifuge tubes and heated to 95°C for 5 min. For the ATP titration reactions, the complete contents of the boiled samples were resolved on 4 – 12% gradient Bis-Tris polyacrylamide gels. For the β-catenin titrations, the boiled reaction samples containing more than 1 μM β-catenin were diluted with 1x SDS-PAGE loading buffer to prevent gel overloading. The samples were diluted to obtain a similar loading concentration of β-catenin as in the boiled sample for the 1.0 μM reaction. 12 μL of the diluted, boiled samples for the 15, 10, 7.5, 5, 2.5 μM reactions and the complete boiled samples from the other reactions (12 μL) were resolved on 4 – 12% gradient Bis-Tris polyacrylamide gels. All polyacrylamide gels were stained with Coomassie prior to drying on chromatography paper (Whatman). To enable quantification of the extent of protein phosphorylation, two-fold serial dilutions of the left-over ATP mixture containing unlabelled ATP and γ-P^{32}-ATP were made for each experiment and specific amounts of the ATP mixture (12.5, 6.25, 3.125, 1.563, 0.781, 0.390, 0.195 pmol) were spotted onto nitrocellulose membrane strips and air-dried. For each experiment, the dried polyacrylamide gels and the corresponding nitrocellulose membrane strips were simultaneously exposed to a phosphorimaging screen (GE Healthcare) and scanned on a Typhoon FLA 9500 biomolecular imager (GE Healthcare).

Control reactions to validate the β-catenin-4A phospho mutant (S33A, S37A, T41A, S45A) and assess GSK3β serine 9 phosphorylation were performed as described above with the following changes: reactions contained 200 μM unlabelled ATP and 0.9 μCi γ-^{32}P-ATP (for radiometric reactions only), 1 μM β-catenin and 200 nM APC. The reactions were terminated after 5 min by pipetting 6 μL of the reaction volume into wells containing 6 μL of 2x SDS-PAGE loading buffer.

The phosphorimaging files were analyzed using ImageJ (Fiji) (Schindelin et al., 2012). The raw files (.gel), which use a square root algorithm for signal compression, were normalized prior to gel densitometry analysis using the ImageJ "Linearize GelData" plugin (https://rsb.info.nih.gov/ij/plugins/linearize-gel-data.html). Background-corrected signal intensities of phosphorylated (^{32}P) β-catenin were converted into picomoles using the standard curve generated from the background-corrected signal intensities of known ATP (γ-^{32}P-ATP) quantities in serial dilutions.

The initial reaction rates (pmol/min) were obtained from the slopes of linear fits to data describing the amounts of generated product (pmol phosphorylated β-catenin) over time (min) at the different concentrations of either ATP or β-catenin. The initial rates were plotted as a function of [ATP] or [β-catenin] by nonlinear least-squares regression fit and kinetic parameters, V_{max} (pmol/μL), K_M (μM) and k_{cat} (min^{-1}) determined using the Michaelis-Menten equation:

$$v_o = \frac{k_{cat} \times [E_T] \times [S]}{K_M + [S]}$$

To calculate k_{cat}, the total enzyme concentration [E_T] in the reactions needs to be known. The precise kinase concentrations in the AXIN1-CK1α-GSK3β complexes are not known in the experimental setup. Therefore, the concentration of the complete AXIN1-kinase complex was used as simplification, giving an [E_T] of 0.9 pmol based on 6 μL of 150 nM AXIN1-kinase complex. (Note that as per convention, [E_T] is not entered as a concentration but as an amount.) Standard errors for V_{max}, K_M and k_{cat} reported in Figure 5C were derived from the non-linear least-squares fits to the Michaelis-Menten equation. For the β-catenin reactions in the absence of APC, which did not saturate, the catalytic efficiencies (k_{cat}/K_M) were obtained from the linear fit to the plots of the initial rates against [β-catenin], and the associated standard errors were derived from the linear regression fit. For reactions reaching saturation, standard errors for k_{cat}/K_M were propagated from individual k_{cat} and K_M values using the following equation:

$$\frac{\delta z}{z} = \sqrt{\left(\frac{\delta x}{x}\right)^2 + \left(\frac{\delta y}{y}\right)^2}$$

x = k_{cat}
y = K_M
z = k_{cat}/K_M
δ = standard error

Mass photometry

Instrument setup

Microscope coverslips (High Precision, No. 1.5, 24 × 50 mm, # 630-2187, VWR) were cleaned twice, sequentially with Milli-Q water, 100% isopropanol, Milli-Q water. Washed coverslips were dried with compressed air. Reusable silicone CultureWell™ gaskets (# GBL103250, Sigma-Aldrich) were cleaned sequentially with Milli-Q water, 100% Isopropanol, Milli-Q water and dried with compressed air. Cleaned gaskets were placed on the cleaned coverslips and mounted on the sample stage of a Refeyn One^{MP} mass photometer (Refeyn Ltd, Oxford, UK). All measurements were performed in acquisition buffer containing 50 mM HEPES-NaOH pH 7.5, 150 mM NaCl and filtered through a 0.2-μm filter. Each measurement was performed in a separate well. Data were acquired using the Refeyn Acquire^{MP} 2.3.1 software. Mass photometry movies of 30,000 frames were recorded from a 10 × 10 μm instrument field of view at a frame rate of 362.4 Hz with a frame binning value of 4, resulting in an effective frame rate of 90.6 Hz. For each acquisition, 15 μL of acquisition buffer were pipetted into a well, and the focal position was identified and secured in place using the autofocus functionality in the software.

Sample preparation and acquisition

Same-day, affinity-purified AXIN1 wild-type and AXIN1 M3 DCs at A_{280} = 0.84 and 0.86, respectively, were subjected to SEC-MALS analysis under conditions similar to those reported above. For accurate detection of single events during data acquisition, the recommended final protein concentration in the acquisition wells should be below 100 nM (Sonn-Segev et al., 2020). As stated above, the precise concentration of the DCs could not be reliably estimated. By determining the A_{280} values of the SEC-MALS input samples and peak elution fractions and performing several test acquisitions, a sample concentration corresponding to an A_{280} of ≈0.01 in the acquisition wells enabled detection of individual single events below saturation levels during acquisition. For instance, 2 μL of a 1:10 dilution in acquisition buffer of the input samples and 2 to 3 μL of undiluted elution fractions were used for the acquisitions. Input samples were diluted just prior to each respective data acquisition. Upon addition of the samples to the wells, data acquisition was started after 10 s to allow for autofocus stabilization.

Data processing

Data were processed and analyzed using Refeyn Discover^{MP} 2.3.0 software by performing three main steps: (1) background removal, (2) identification of particle landing events on the coverslip acquisition field, and (3) particle fitting to extract maximum contrast. The following default fitting parameters were used; threshold 1 (related to the particle contrast relative to the background noise) was set to 1.5, threshold 2 (related to the radial symmetry of the particle signature) was set to 0.25, and 5 ratiometric frames were binned. Individual particle contrasts from each individual movie were converted to mass using a contrast-to-mass (C2M) calibration. From these data, kernel density estimates (KDEs) were generated for each sample using a Gaussian kernel with a fixed bandwidth of 25 kDa.

Calibration procedure: Contrast-to-mass calibration was performed in the acquisition buffer. NativeMark™ unstained protein standard (# LC0725, Thermo Scientific), which contains proteins in the range of 20 to 1200 kDa, was diluted 1:100 in acquisition buffer, and 2.5 μL were added to an acquisition well for measurement. The following masses were used to generate a standard calibration curve in the Discover^{MP} software: 66, 146, 480 and 1048 kDa.

In-silico stoichiometry modeling of the DC and estimation of DC molar concentrations

Inputs

The following inputs were used for modeling of DC stoichiometries:

i) Predicted molecular weights and extinction coefficients (ε) of the individual proteins in the DC, from Expasy ProtParam (Gasteiger et al., 2005). As protein complexes can have similar molecular weights without sharing similar ε, by taking ε into account, we were able to narrow down the modeled stoichiometries.
ii) Means of molecular weights obtained from individual SEC-MALS data slices (M_i) for the DC with AXIN1 WT or DC with AXIN1 M3 across the peak (DC AXIN1 WT: 2.3 – 3.0 mL; DC AXIN1 M3: 2.5 – 3.2 mL). Mean molecular weights are number average molecular weights (M_n).
iii) Mean molar extinction coefficients (in M^{-1} cm^{-1}) of the DC calculated from the parameters recorded by SEC-MALS for individual data slices as follows:

The differential refractive index (dRI) of a sample solution relative to the solvent is directly proportional to the sample concentration. Hence, the sample concentration (c) can be calculated from the dRI measurements and the protein-specific refractive index increment dn/dc of 0.185 mL g^{-1} as follows:

$$c = \frac{dRI}{\frac{dn}{dc}}$$

With the concentration determined and converted into g L^{-1}, the extinction coefficient (ε) in L g^{-1} cm^{-1} is calculated using the Beer-Lambert law equation:

$$\varepsilon = \frac{A}{lc}$$

where A is absorbance measured at 280 nm (A_{280}) in absorbance units (AU), and l is length of light path (1 cm). ε is then converted into the molar units (M^{-1} cm^{-1}) by multiplying ε with the calculated molar masses (g mol^{-1}) from MALS.

Performing these calculations across the elution peak, we obtained the concentrations of the DC at the detector in SEC-MALS for each data slice for the SEC-MALS experiment shown in Figure 1E. The maximum concentration measured for the DC with wild-type AXIN1 was 56 nM; that for the DC with M3 AXIN1 was 94 nM. We also calculated the average ε of the DC (2,024,161 ± 367,667 M^{-1} cm^{-1} for the DC with wild-type AXIN1 and 980,087 ± 275,179 M^{-1} cm^{-1} for that with M3 AXIN1; variance, SD). This enabled us to estimate the molar concentrations of the DC in the SEC-MALS input samples: 415 nM for the DC with wild-type AXIN1, and 877 nM for that with M3 AXIN1. Note, however, that due to the highly concentration-dependent composition of the DC, the molar extinction coefficients will also vary with concentration, especially for the DC containing wild-type AXIN1.

Conditions

The DC stoichiometries are modeled on the conditions that:

1. DC components are APC, AXIN1, CK1α, GSK3β and β-catenin.
2. The molecular weight of the DC is the sum of molecular weights of its components at a given stoichiometry.
3. The extinction coefficient (ε) of the DC is the sum of the individual ε values of its components at a given stoichiometry.
4. Molecular weight and ε of the DC at the modeled stoichiometry must satisfy the input values ± 1.96 standard deviations from the mean values calculated from SEC-MALS (95% confidence interval).
5. At least one of each component is present in the complex.
6. The number of molecules in the DC takes the form of a positive integer.
7. DC stoichiometries are modeled by incrementing the number of molecules of each component by 1 in the order of conditions 8 – 14.
8. At least two AXIN1 molecules are present in the DC with wild-type AXIN1, as we observe that AXIN1 polymerization is a major DC size determinant.
9. CK1α and GSK3β occupancy on AXIN1 is at least 50%, and at most both kinases are bound to AXIN1. This is to mitigate possible modeling errors in the case of incomplete occupancy of kinase binding sites on AXIN1.
10. Each APC binds at least one and a maximum of ten β-catenin molecules through four 15R and six 20R repeats, as 20R2 does not bind β-catenin (Liu et al., 2006).
11. Each APC binds one to three AXIN1 molecules via three available SAMP repeats.
12. At least one molecule of β-catenin is bound to each AXIN1 or APC molecule present in the complex.
13. For a given AXIN1:APC stoichiometry, at least one more molecule of β-catenin is present in the DC containing wild-type AXIN1 compared to the DC containing AXIN1 M3.
14. The number of β-catenin molecules in the DC with AXIN1 M3 does not exceed the number of β-catenin molecules in the DC with wild-type AXIN1.

Conditions 9 and 12-14 are based on the SDS-PAGE analysis of affinity-purified material and the elution fractions from SEC-MALS. As a consequence of condition 9, the number of β-catenin molecules can increase if AXIN1 is not saturated with kinases.

The algorithm first imports the inputs, sets the constraints on the stoichiometries that can be modeled and starts from the minimum number of AXIN1 molecules (AXIN1 M3 n = 1, AXIN1 WT n = 2). For each AXIN1 molecule, the algorithm then explores all possible combinations of the DC in the order of conditions specified above. Modeled stoichiometries which satisfy all of the set theoretical limits on the APC:AXIN1:kinases:β-catenin ratios **and** attain a molecular weight and ε falling within the range calculated from SEC-MALS are written in the final output file. The molecular weights plotted in Figure 1G correspond to number average molecular weights (M_n). The Python scripts for DC stoichiometry modeling are available for download as Data S1 in the online version of the article.

Antibodies and reagents

Primary antibodies used were anti-β-catenin (# 610153, BD Transduction Laboratories), anti-phospho-β-catenin pS33, pS37, pT41 (# 9561, Cell Signaling Technology), anti-non-phospho-β-catenin S33, S37, T41 (#8814, Cell Signaling Technology), anti-phospho-β-catenin pS45 (# 9564, Cell Signaling Technology), anti-StrepMAB-Classic (# 2-1507-001, IBA Life Sciences) to detect dStrepII-AXIN1, anti-AXIN1 (# 2087, Cell Signaling Technology), anti-APC (# NB100-667, Novus Biologicals), anti-GSK3β (# 12456, Cell Signaling Technology), anti-phospho-GSK3β pS9 (# 5558, Cell Signaling Technology), anti-ubiquitin K48 linkage-specific (# ab140601, Abcam), anti-ubiquitin K63 linkage-specific (# ab179434, Abcam), anti-ubiquitin P4D1 clone (# BML-PW0930, Enzo Lifesciences). Secondary antibodies for immunoblotting with detection using the Odyssey infrared imaging system (LI-COR) were IRDye 800CW donkey anti-mouse (# 926-32212, LI-COR), IRDye 800CW donkey anti-rabbit (# 926-32213, LI-COR), IRDye 680RD

donkey anti-mouse (# 926-68072, LI-COR). Recombinant human poly-ubiquitin chains were obtained from commercial source: K63-linked chains (# UC-330, R&D Systems), K48-linked chains (# UC-230, R&D Systems).

QUANTIFICATION AND STATISTICAL ANALYSIS

Various β-catenin signals from *in-vitro* ubiquitylation reactions were analyzed using ImageQuant software (GE Healthcare). Phosphorylated β-catenin levels from *in vitro* γ-^{32}P-ATP kinetic reactions were analyzed using ImageJ (Fiji) 1.53c software, and the kinetic parameters were determined as described above using GraphPad Prism 8.2.1 software. All statistical analyses were performed using GraphPad Prism 8.2.1 software. Statistical significance was calculated using either an unpaired Student's t test or one-way ANOVA analysis with Bonferroni test for multiple comparisons. $p < 0.05$ was considered significant.

Molecular Cell, Volume *81*

Supplemental information

Reconstitution of the destruction complex defines roles of AXIN polymers and APC in β-catenin capture, phosphorylation, and ubiquitylation

Michael Ranes, Mariola Zaleska, Saira Sakalas, Ruth Knight, and Sebastian Guettler

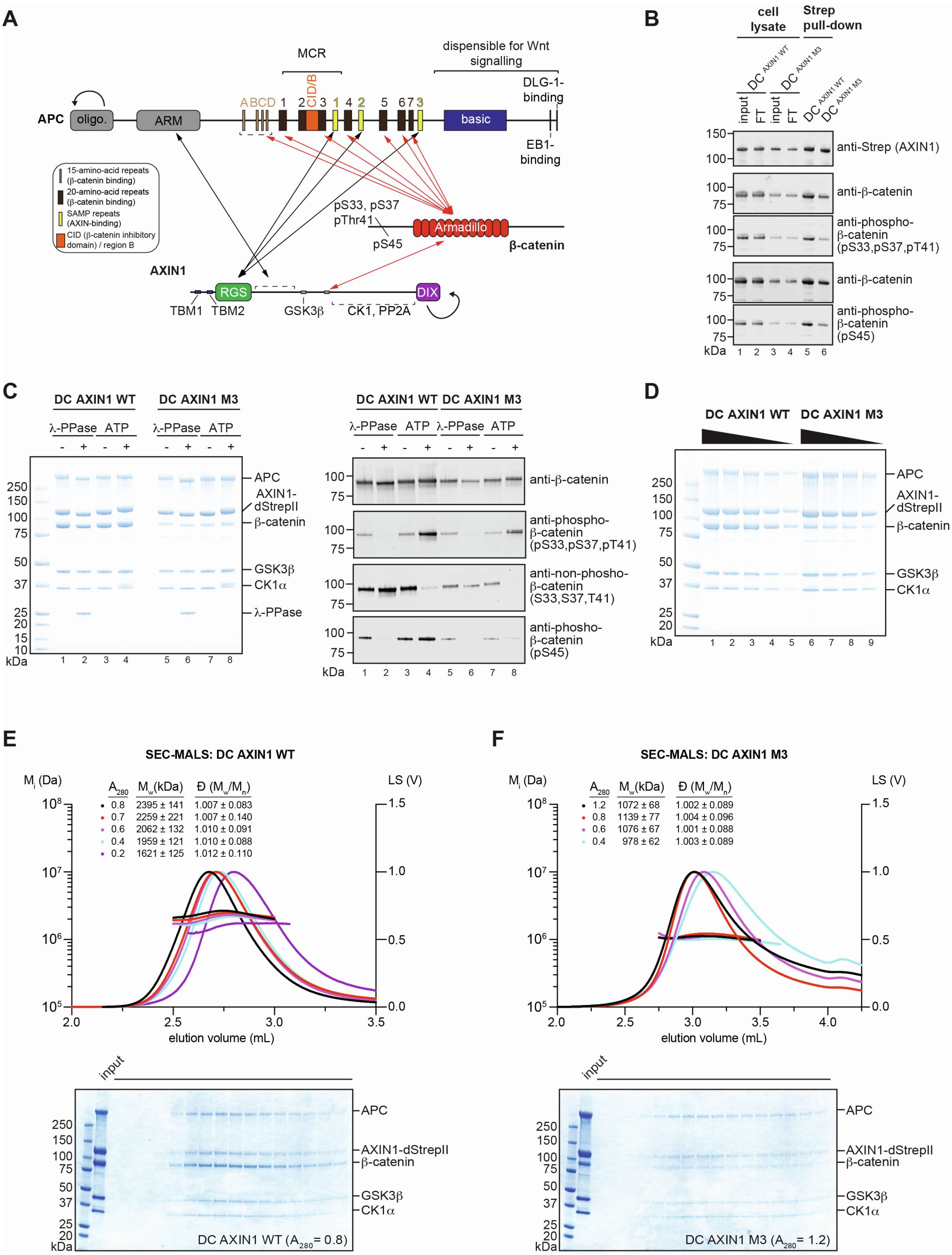

Figure S1. AXIN1 polymerisation is a major contributor to the concentration-dependent stoichiometry of the DC. (related to Figure 1)

(**A**) Schematic representation of core DC components. **APC:** APC 15- and 20-amino-acid-repeats (15R and 20R) bind β-catenin (except for 20R2) (Eklof Spink et al., 2001; Ha et al.,

2004). SAMP repeats bind AXIN1/2 (Spink et al., 2000). 20R2 and the adjacent β-catenin-inhibitory domain (CID) (a.k.a. region B) are proposed to bind α-catenin or regulate AXIN1/2 binding to APC (Choi et al., 2013; Pronobis et al., 2015). The MCR (mutation cluster region) refers to a CRC mutation hotspot (Kohler et al., 2008). Oligo., oligomerisation domain; ARM, armadillo repeat domain. **Beta-catenin:** ARM, armadillo repeat domain. **AXIN1:** TBM, tankyrase-binding motif (Morrone et al., 2012); RGS, regulator of G-protein signalling domain; DIX, polymerising domain present in Dishevelled and AXIN (Fiedler et al., 2011), sometimes also referred to as DAX domain. Kinase binding sites in AXIN1 (Stamos and Weis, 2013) are indicated. Mutual interaction sites between APC, AXIN1 and β-catenin are indicated by arrows. (**B**) Immunoblot analysis of the expressed and affinity-purified recombinant DC containing either wild-type or polymerisation-deficient (M3) AXIN1 to probe the β-catenin phosphorylation status. The figure, which corresponds to Figure 1B, shows one of three representative experiments that form the basis of Figure 1C. Input, lysates; FT, flow-through. (**C**) Further analysis of the phosphorylation status of the affinity-purified recombinant DC containing either wild-type or polymerisation-deficient (M3) AXIN1. Samples were either left untreated, incubated with λ-phosphatase for de-phosphorylation, or incubated with ATP for hyperphosphorylation. Left, Coomassie-stained SDS-PAGE gel; right, immunoblot analysis of the same samples. (**D**) Coomassie-stained SDS-PAGE gel of the indicated input samples analysed by SEC-MALS, corresponding to Figure 1D. (**E** and **F**) SEC-MALS analyses of the DC containing either wild-type AXIN1 (panel E) or polymerisation-deficient AXIN1 M3 (panel F), corresponding to Figure 1D. Chromatograms show plots of light scattering intensity at 90° and calculated molecular weights per measurement slice (M_i). The tables show the measured A_{280} of the input material*, weight-average molecular weights (M_w) and dispersities (Đ) over the peak areas with uncertainties (see Methods). Eluates across the peaks were fractionated and analysed by SDS-PAGE and Coomassie staining.

*Note that in the absence of a known stoichiometry, the concentration of the DC cannot be reliably calculated.

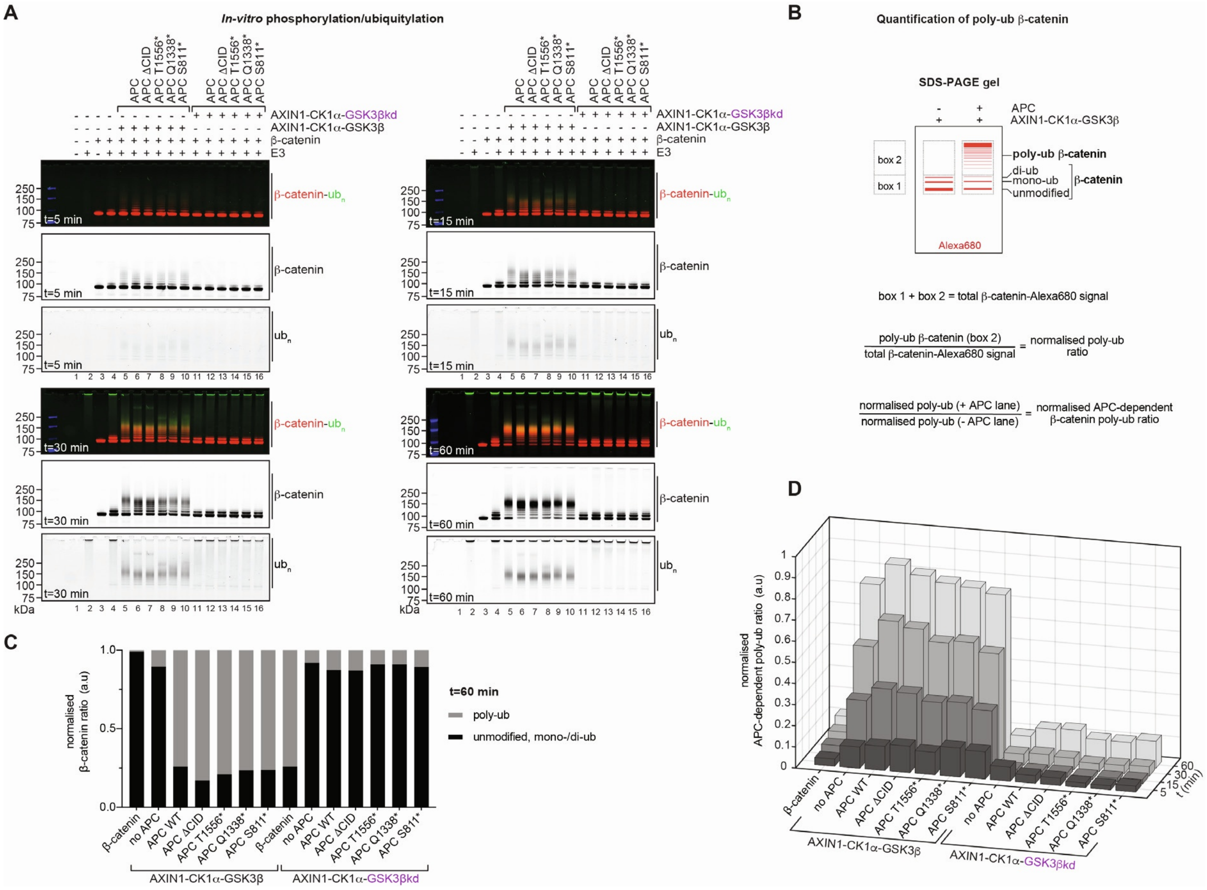

Figure S2. Reconstitution of the biochemical activity of the DC: β-catenin poly-ubiquitylation. (related to Figure 3)

(**A**) *In-vitro* DC activity assay as in Figure 3D, with different reaction times. The data for the 60-min time point are identical to those shown in Figure 3D and are included for comparison. (**B**) Procedure for quantifying β-catenin poly-ubiquitylation and the contribution of APC. (**C**) Normalised ratio of unmodified + mono-/di-ubiquitylated vs. poly-ubiquitylated β-catenin for the 60-min time point. (**D**) Normalised APC-dependent β-catenin poly-ubiquitylation, calculated for different reaction time points, as shown in A and B (n=1 representative experiment).

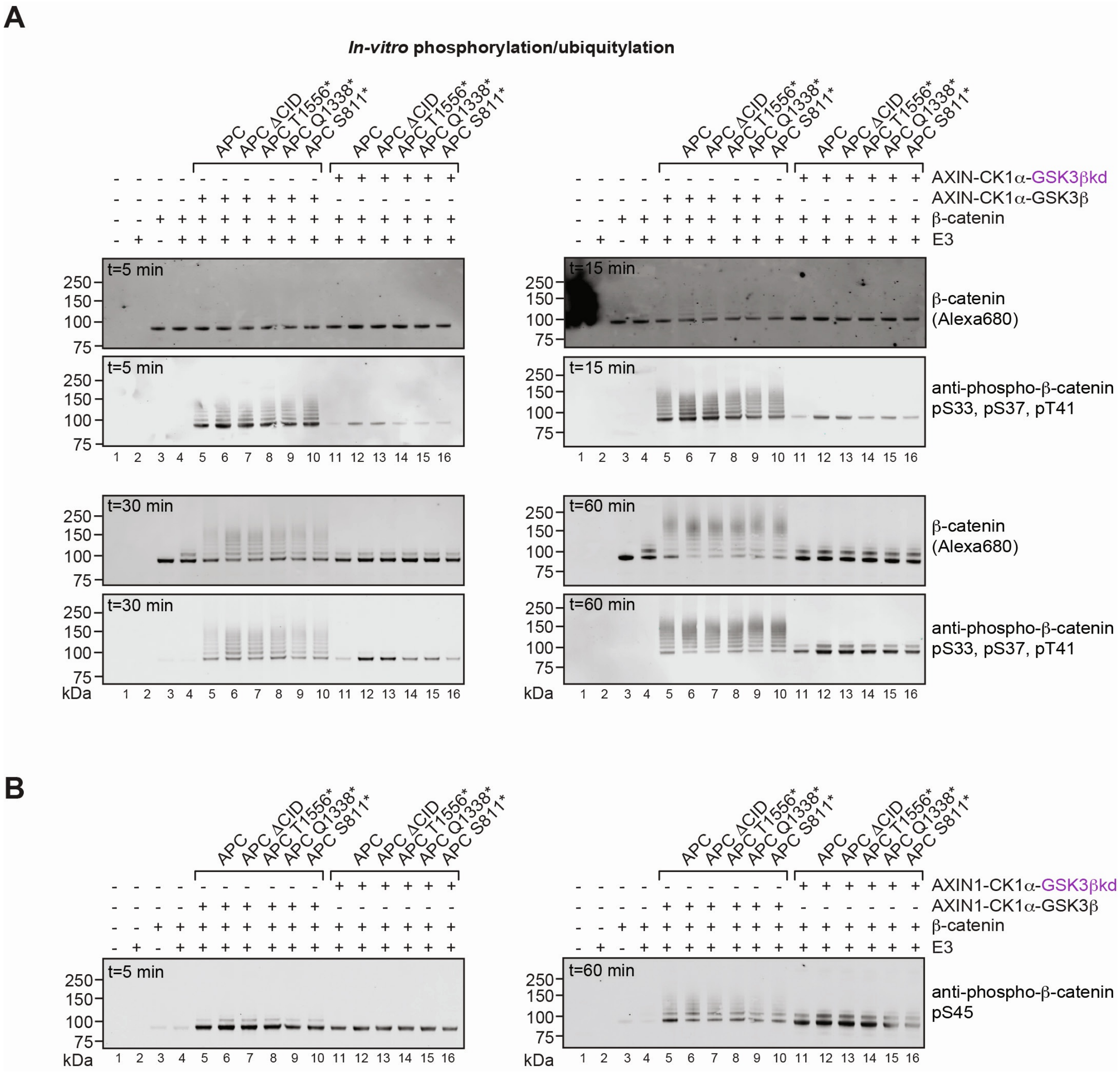

Figure S3. Reconstitution of the biochemical activity of the DC: β-catenin phosphorylation. (related to Figure 3)

(**A**) Analysis of GSK3β-dependent phosphorylation of β-catenin in the samples also analysed in Figures 3D and S2. The same gels as those shown in Figure 3D and S2A were used for transfer and immunoblotting. (**B**) Analysis of CK1α-dependent phosphorylation of β-catenin in the same samples from the indicated reaction time points.

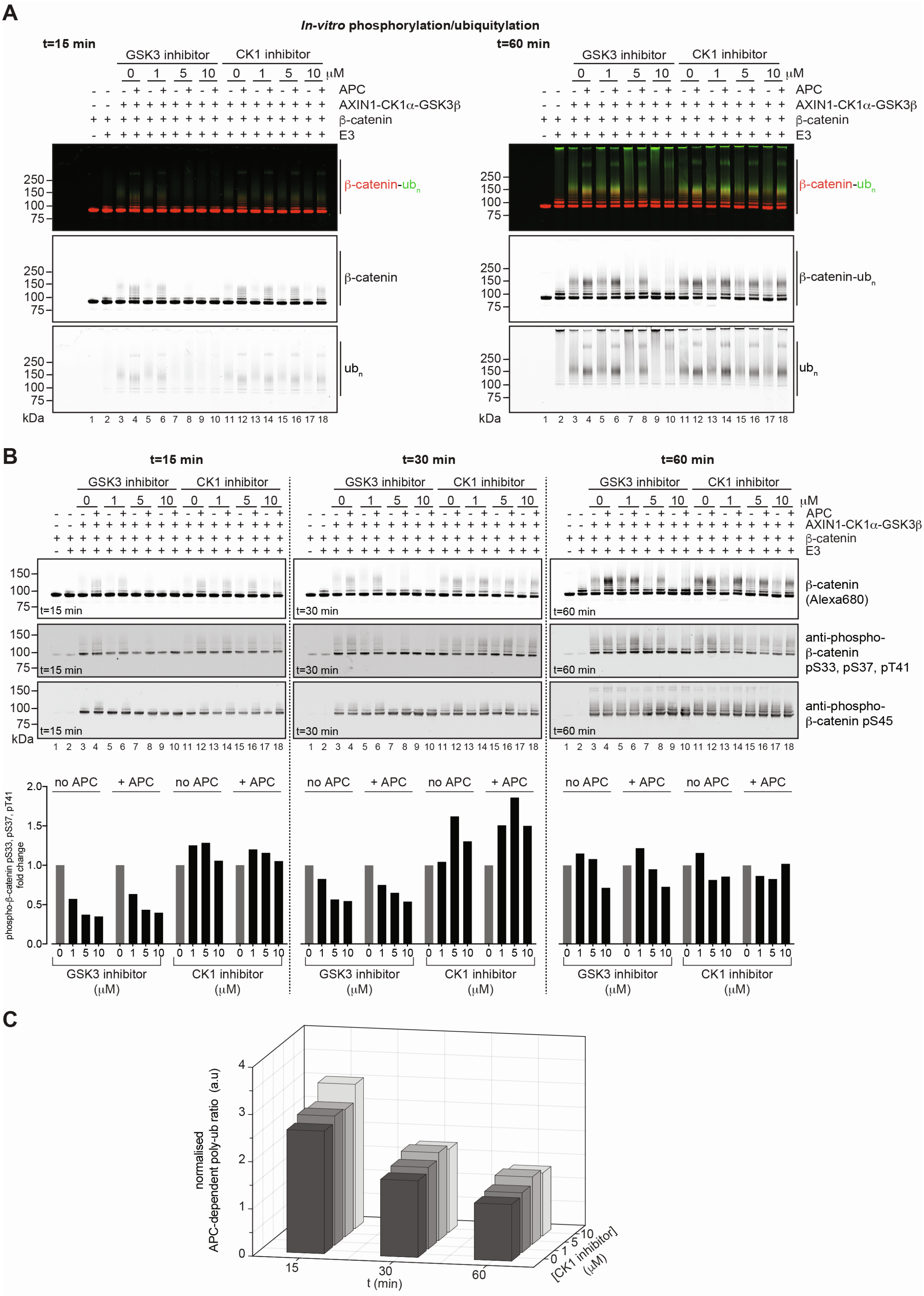

Figure S4. GSK3β inhibition sensitises the *in-vitro* DC phosphorylation/ubiquitylation assay to APC addition. (related to Figure 3)

(**A**) *In-vitro* DC activity assays as in Figure 3F, with different reaction times. Data from the 30-min time point are shown in Figure 3F. (**B**) Analysis of GSK3β- and CK1α-dependent phosphorylation of β-catenin at the indicated reaction time points. The same gels as those shown in A and Figure 3F were used for transfer and immunoblotting. The phospho-β-catenin changes upon inhibitor titration were quantified and expressed relative to those in the absence of kinase inhibitor (set to 1, grey bars) for each condition. (**C**) Normalised APC-dependent β-catenin poly-ubiquitylation, calculated for different reaction time points and different CK1 inhibitor concentrations (n=1 representative experiment).

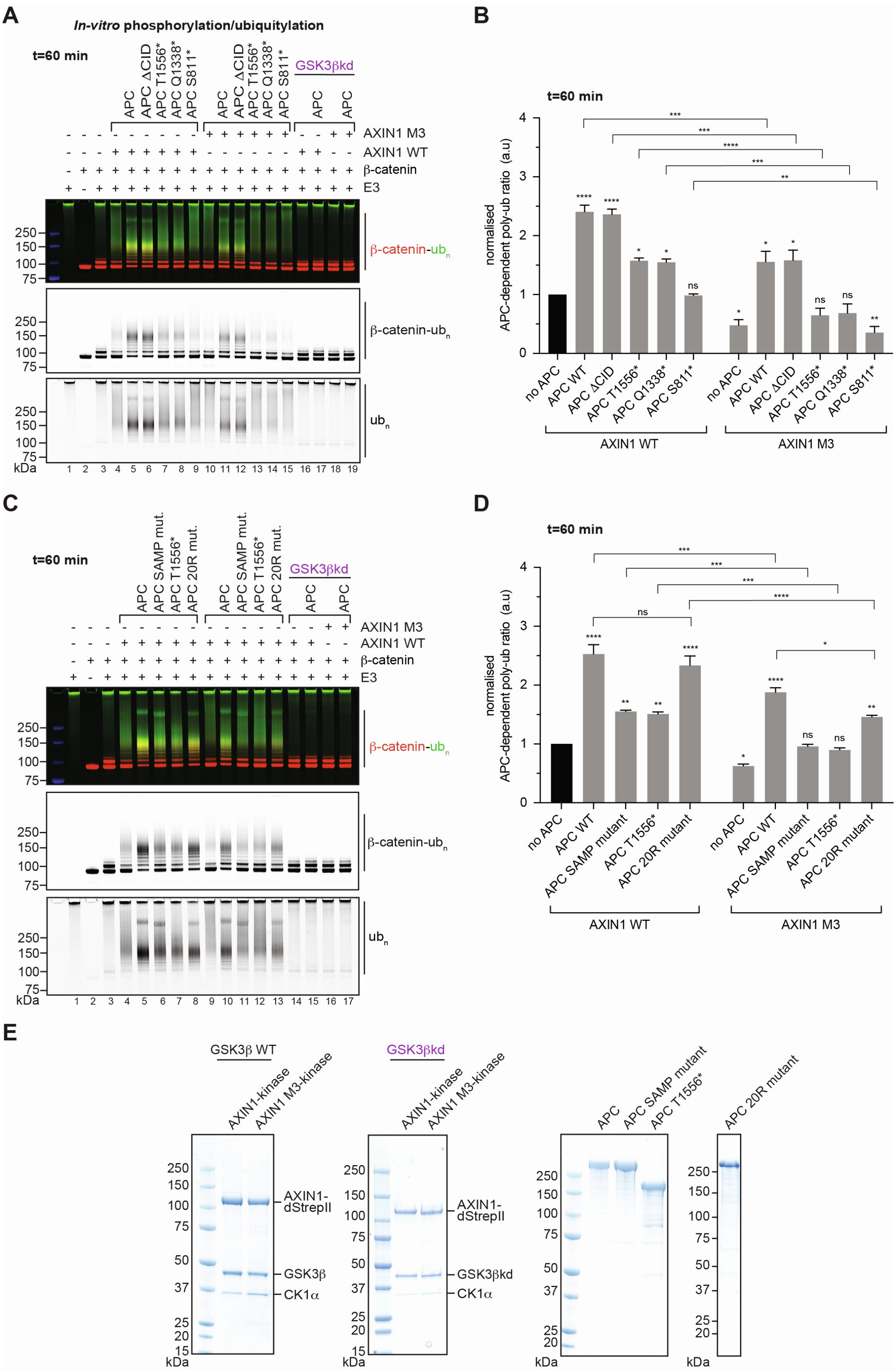

Figure S5. Oncogenic APC truncation and impairment of AXIN1 polymerisation limit DC activity. (related to Figure 4)

(**A** and **C**), *In-vitro* DC activity assay in the presence of 5 μM GSK3 inhibitor. Data are displayed as in Figures 4A and 4C but are from the 60-min reaction time point. (**B** and **D**) Quantification of the APC-dependent poly-ubiquitylation of β-catenin as in Figures 4B and 4D, but from the 60-min reaction time point. Data are means from three independent experiments with error bars representing SEM. Statistical analyses were performed using one-way ANOVA analysis with Bonferroni test for multiple comparisons. Asterisks above histograms (without brackets) refer to comparisons to the no-APC condition (black bar). *P<0.05; **P<0.01; ***P<0.001; ****P<0.0001 ; ns = not significant. See Tables S7 and S9 for details of statistical analyses. (**E**) Coomassie-stained SDS-PAGE gels for purified AXIN1-kinase complexes, wild-type APC, APC SAMP mutant, APC T1556* and APC 20R phospho-site mutant.

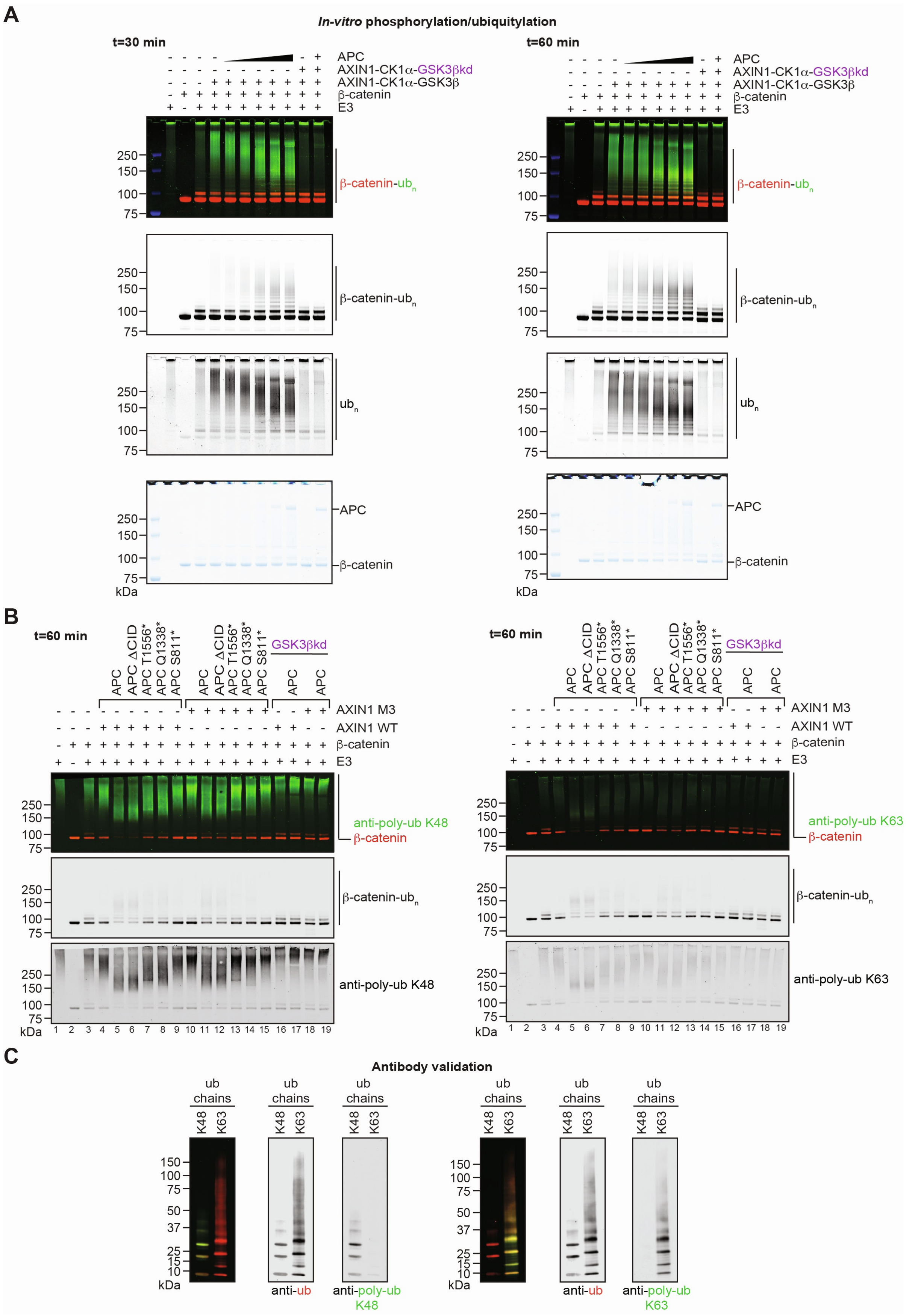

Figure S6. *In-vitro* modification of β-catenin by K48-linked poly-ubiquitin. (related to Figure 4)

(**A**) *In-vitro* DC activity assay in the presence of variable amounts of APC (25, 50, 100, 200, 400 nM) and 5 μM GSK3 inhibitor. (**B**) *In-vitro* DC activity assay in the presence of 10 μM GSK3 inhibitor. Reactions were probed with antibodies against K48-linked (left) or K63-linked poly-ubiquitin (right). (**C**) Validation of the linkage-specific poly-ubiquitin antibodies detecting K48-linked and K63-linked poly-ubiquitin. An anti-ubiquitin antibody was used to detect both chain types.

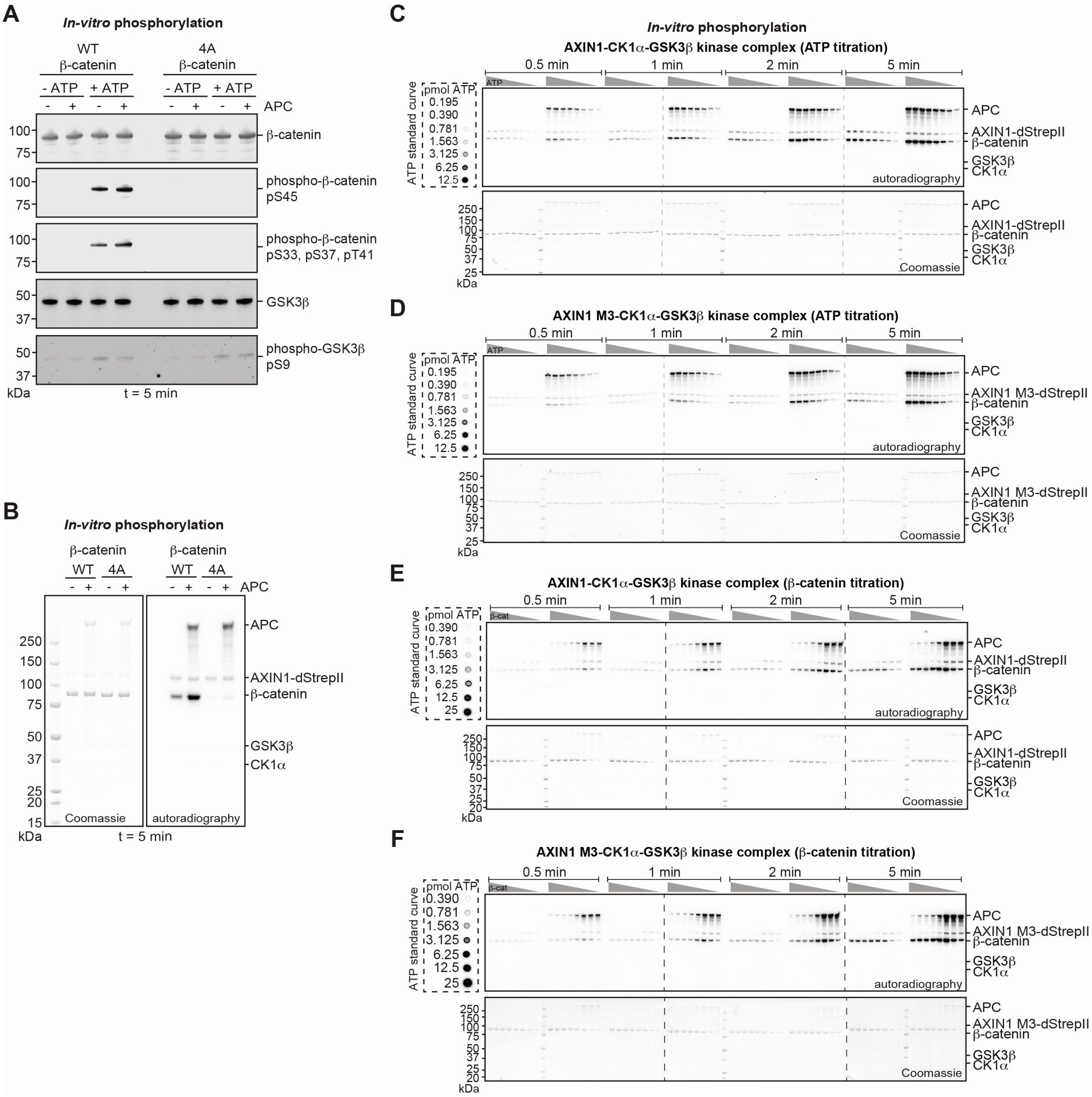

Figure S7. ***In-vitro* β-catenin phosphorylation assays.** (related to Figure 5)

(**A**) Validation of the β-catenin-4A phospho-mutant (S33A, S37A, T41A, S45A) and assessment of GSK3β serine 9 phosphorylation by *in-vitro* phosphorylation and immunoblotting. AXIN1-kinase complex was incubated with the indicated β-catenin variants in the absence and presence of ATP. (**B**) Assessment of overall β-catenin *in-vitro* phosphorylation, not limited to phosphodegron phosphorylation, by incubation with ATP/γ-^{32}P-ATP and autoradiography. APC was added to the reactions as indicated. (**C, D, E, F**) Representative Coomassie-stained gels, their respective autoradiographs and autoradiographs of ATP standard curve serial dilutions for kinetic β-catenin phosphorylation assays. Prior to SDS-PAGE analysis, samples containing >1 μM β-catenin were diluted to 1 μM to prevent gel overloading; the raw values were corrected accordingly during data analysis.

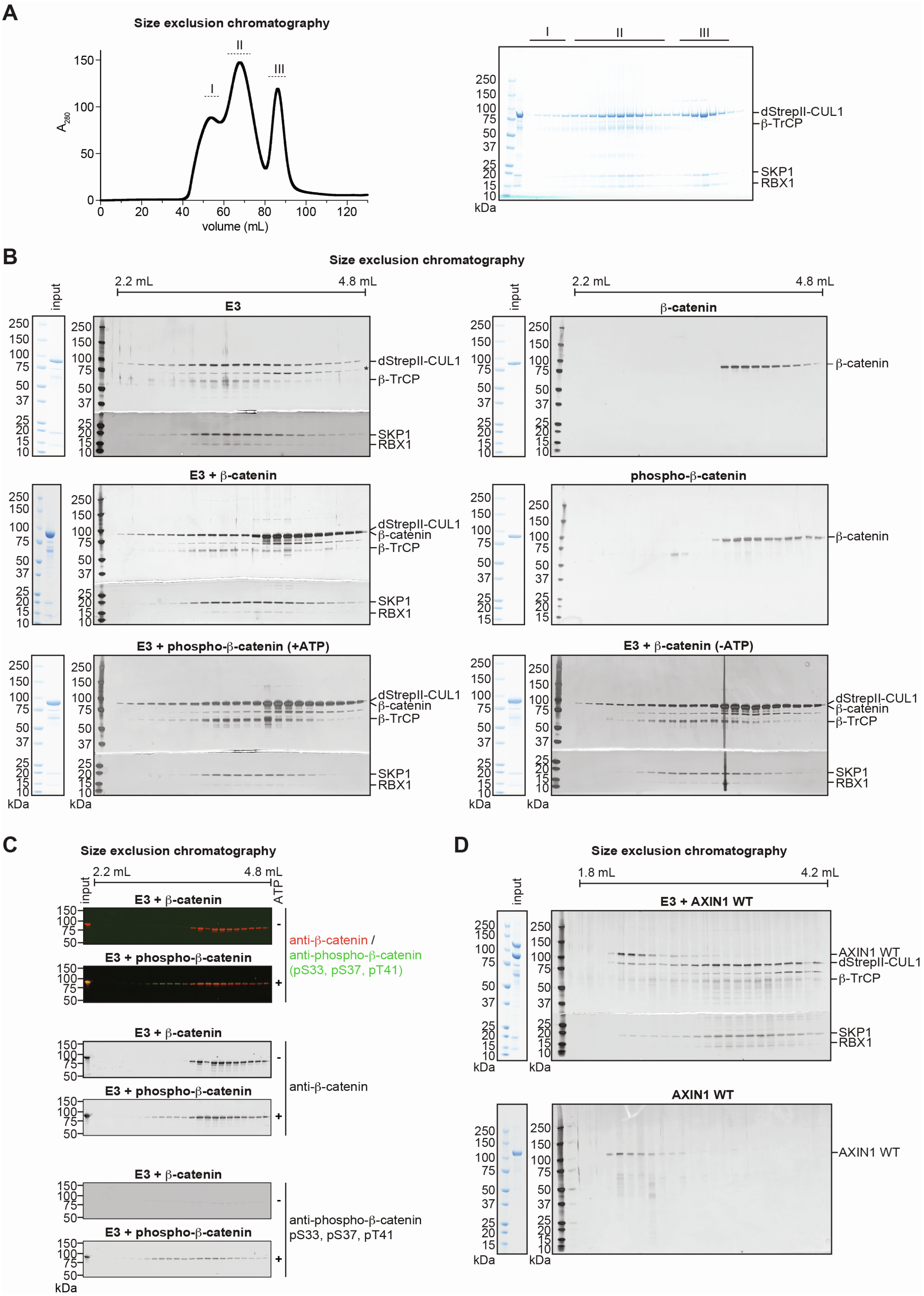

Figure S8. Size exclusion chromatography analysis of SCF$^{\beta\text{-TrCP}}$ with β-catenin or AXIN1. (related to Figure 6)

(**A**) Final (size exclusion chromatography) step in the purification of the SCF$^{\beta\text{-TrCP}}$ complex, with chromatogram (left) and analysis of peak fractions by SDS-PAGE and Coomassie staining (right). Peaks 2 and 3 correspond to the full complex and one lacking β-TrCP, respectively. (**B**) Analytical size exclusion chromatography to study phosphorylation-dependent interactions of the SCF$^{\beta\text{-TrCP}}$ complex with β-catenin. Input samples and SEC fractions were analysed by SDS-PAGE and Coomassie or silver staining, respectively. β-catenin was partially phosphorylated ("phospho-β-catenin") by incubation with sub-stoichiometric amounts of an AXIN1-CK1α-GSK3β complex and ATP. (**C**) Analysis of samples from panel B by immunoblotting, using antibodies recognising either total or GSK3β-phosphorylated β-catenin. (**D**) Analytical size exclusion chromatography to study interaction of the SCF$^{\beta\text{-TrCP}}$ complex with AXIN1. Reference runs with loading of the equivalent fractions are found in Figure 6. Samples were analysed as for panel B.

Table S1. *In-silico* DC stoichiometry modelling corresponding to Figure 1G.

DC AXIN1	Number of molecules					M_n (kDa)	Maximum stoichiometry (APC : AXIN1 : kinases : β-catenin)
	APC	AXIN1	CK1α	GSK3β	β-catenin		
M3	2	2	1 – 2	1 – 2	4 – 6	1250 – 1507	1 : 1 : 1 : 3
	1	2	1 – 2	1 – 2	3 – 5	853 – 1110	1 : 2 : 2 : 5
	1	3	2 – 3	2 – 3	4 – 5	1124 – 1295	1 : 3 : 3 : 5
WT	2	2	1 – 2	1 – 2	12 – 22	1934 – 2875	1 : 1 : 1 : 11
	3	3	2 – 3	2 – 3	13 – 17	2517– 2944	1 : 1 : 1 : 5.7
	3	4	2 – 4	2 – 4	13 – 17	2616 – 2959	1 : 1.3 : 1.3 : 5.7
	2	3	2 – 3	2 – 3	9 – 21	1863 – 2975	1 : 1.5 : 1.5 : 10.5
	3	5	3 – 5	3 – 5	13 – 15	2802 – 2973	1 : 1.7 : 1.7 : 5
	2	4	2 – 4	2 – 4	9 – 20	1963 – 2950	1 : 2 : 2 : 10
	2	5	3 – 5	3 – 5	9 – 18	2148 – 2964	1 : 2.5 : 2.5 : 9
	2	6	3 – 6	3 – 6	9 – 15	2248 – 2971	1 : 3 : 3 : 8.5
	1	3	2 – 3	2 – 3	11 – 13	1722 – 1979	1: 3: 3: 13 (fully saturated unit of the DC)

Table S2. One-way ANOVA analysis corresponding to Figure 2D and STAR Methods (Quantification and statistical analysis).

Bonferroni's multiple comparisons test	Mean diff.	95.00% CI of diff.	Significant?	Summary	Adjusted P value
DC-APC wild-type vs. DC-APC T1556*	0.5053	0.3200 to 0.6905	Yes	****	<0.0001
DC-APC wild-type vs. DC-APC Q1338*	0.4397	0.2544 to 0.6249	Yes	****	<0.0001
DC-APC wild-type vs. DC-APC S811*	0.5649	0.3797 to 0.7502	Yes	****	<0.0001
DC-APC wild-type vs. DC-lacking APC	0.8597	0.6744 to 1.045	Yes	****	<0.0001
DC-APC T1556* vs. DC-APC Q1338*	-0.06559	-0.2508 to 0.1197	No	ns	>0.9999
DC-APC T1556* vs. DC-APC S811*	0.0597	-0.1255 to 0.2449	No	ns	>0.9999
DC-APC T1556* vs. DC-lacking APC	0.3544	0.1692 to 0.5397	Yes	****	<0.0001
DC-APC Q1338* vs. DC-APC S811*	0.1253	-0.05995 to 0.3105	No	ns	0.4774
DC-APC Q1338* vs. DC-lacking APC	0.42	0.2348 to 0.6052	Yes	****	<0.0001
DC-APC S811* vs. DC-lacking APC	0.2947	0.1095 to 0.4800	Yes	***	0.0005

Table S3. One-way ANOVA analysis corresponding to Figure 2E and STAR Methods (Quantification and statistical analysis).

Bonferroni's multiple comparisons test	Mean diff.	95.00% CI of diff.	Significant?	Summary	Adjusted P value
DC-APC wild-type vs. DC-APC T1556*	0.466	0.1120 to 0.8201	Yes	**	0.0082
DC-APC wild-type vs. DC-APC Q1338*	0.3814	0.02740 to 0.7355	Yes	*	0.0317
DC-APC wild-type vs. DC-APC S811*	0.6791	0.3251 to 1.033	Yes	***	0.0004
DC-APC wild-type vs. DC-lacking APC	0.7478	0.3937 to 1.102	Yes	***	0.0002
DC-APC T1556* vs. DC-APC Q1338*	-0.08457	-0.4386 to 0.2695	No	ns	>0.9999
DC-APC T1556* vs. DC-APC S811*	0.2131	-0.1410 to 0.5671	No	ns	0.5653
DC-APC T1556* vs. DC-lacking APC	0.2817	-0.07231 to 0.6358	No	ns	0.1725
DC-APC Q1338* vs. DC-APC S811*	0.2977	-0.05639 to 0.6517	No	ns	0.1309
DC-APC Q1338* vs. DC-lacking APC	0.3663	0.01226 to 0.7203	Yes	*	0.0407
DC-APC S811* vs. DC-lacking APC	0.06864	-0.2854 to 0.4227	No	ns	>0.9999

Table S4. One-way ANOVA analysis corresponding to Figure 2F and STAR Methods (Quantification and statistical analysis).

Bonferroni's multiple comparisons test	Mean diff.	95.00% CI of diff.	Significant?	Summary	Adjusted P value
DC-APC wild-type vs. DC-APC T1556*	0.4528	0.08416 to 0.8214	Yes	*	0.0134
DC-APC wild-type vs. DC-APC Q1338*	0.3932	0.02454 to 0.7618	Yes	*	0.0337
DC-APC wild-type vs. DC-APC S811*	0.6682	0.2996 to 1.037	Yes	***	0.0007
DC-APC wild-type vs. DC-lacking APC	0.7858	0.4171 to 1.154	Yes	***	0.0002
DC-APC T1556* vs. DC-APC Q1338*	-0.05962	-0.4282 to 0.3090	No	ns	>0.9999
DC-APC T1556* vs. DC-APC S811*	0.2154	-0.1532 to 0.5840	No	ns	0.6283
DC-APC T1556* vs. DC-lacking APC	0.333	-0.03563 to 0.7016	No	ns	0.0894
DC-APC Q1338* vs. DC-APC S811*	0.275	-0.09359 to 0.6436	No	ns	0.2341
DC-APC Q1338* vs. DC-lacking APC	0.3926	0.02399 to 0.7612	Yes	*	0.034
DC-APC S811* vs. DC-lacking APC	0.1176	-0.2510 to 0.4862	No	ns	>0.9999

Table S5. One-way ANOVA analysis corresponding to Figure 3E and STAR Methods (Quantification and statistical analysis).

Bonferroni's multiple comparisons test	Mean diff.	95.00% CI of diff.	Significant?	Summary	Adjusted P value
no APC vs. APC WT	-0.1165	-0.1709 to -0.06203	Yes	****	<0.0001
no APC vs. APC ΔCID	-0.09181	-0.1463 to -0.03737	Yes	***	0.0009
no APC vs. APC T1556*	-0.04927	-0.1037 to 0.005174	No	ns	0.0906
no APC vs. APC Q1338*	-0.04389	-0.09834 to 0.01055	No	ns	0.1681
no APC vs. APC S811*	0.007780	-0.04666 to 0.06222	No	ns	>0.9999
APC WT vs. APC ΔCID	0.02466	-0.02978 to 0.07910	No	ns	>0.9999
APC WT vs. APC T1556*	0.06721	0.01276 to 0.1216	Yes	*	0.0119
APC WT vs. APC Q1338*	0.07258	0.01814 to 0.1270	Yes	**	0.0066
APC WT vs. APC S811*	0.1243	0.06981 to 0.1787	Yes	****	<0.0001

Table S6. One-way ANOVA analysis corresponding to Figure 4B and STAR Methods (Quantification and statistical analysis).

Bonferroni’s multiple comparisons test	Mean diff.	95.00% CI of diff.	Significant?	Summary	Adjusted P value
no APC vs. APC WT	-5.037	-6.880 to -3.194	Yes	****	<0.0001
no APC vs. APC ΔCID	-5.158	-7.001 to -3.315	Yes	****	<0.0001
no APC vs. APC T1556*	-1.855	-3.698 to -0.01211	Yes	*	0.0474
no APC vs. APC Q1338*	-1.847	-3.690 to -0.004193	Yes	*	0.0491
no APC vs. APC S811*	-0.06197	-1.905 to 1.781	No	ns	>0.9999
no APC vs. no APC_AXIN M3	0.5509	-1.292 to 2.394	No	ns	>0.9999
no APC vs. APC WT_AXIN M3	-1.500	-3.342 to 0.3434	No	ns	0.2129
no APC vs. APC ΔCID_AXIN M3	0.4440	-1.399 to 2.287	No	ns	>0.9999
no APC vs. APC T1556*_AXIN M3	0.3777	-1.465 to 2.221	No	ns	>0.9999
no APC vs. APC Q1338*_AXIN M3	0.7412	-1.102 to 2.584	No	ns	>0.9999
no APC vs. APC S811*_AXIN M3	-1.907	-3.750 to -0.06458	Yes	*	0.0377
APC WT vs. APC WT_AXIN M3	3.538	1.695 to 5.380	Yes	****	<0.0001
APC ΔCID vs. APC ΔCID_AXIN M3	3.250	1.408 to 5.093	Yes	****	<0.0001
APC T1556* vs. APC T1556*_AXIN M3	2.299	0.4561 to 4.142	Yes	**	0.0066
APC Q1338* vs. APC Q1338*_AXIN M3	2.225	0.3819 to 4.068	Yes	**	0.0092
APC S811* vs. APC S811*_AXIN M3	0.8031	-1.040 to 2.646	No	ns	>0.9999

Table S7. One-way ANOVA analysis corresponding to Figure S5B, which is related to Figure 4, and STAR Methods (Quantification and statistical analysis).

Bonferroni's multiple comparisons test	Mean diff.	95.00% CI of diff.	Significant?	Summary	Adjusted P value
no APC vs. APC WT	-1.406	-1.925 to -0.8878	Yes	****	<0.0001
no APC vs. APC ΔCID	-1.362	-1.881 to -0.8434	Yes	****	<0.0001
no APC vs. APC T1556*	-0.5749	-1.093 to -0.05630	Yes	*	0.0207
no APC vs. APC Q1338*	-0.5479	-1.066 to -0.02928	Yes	*	0.0317
no APC vs. APC S811*	0.01609	-0.5025 to 0.5347	No	ns	>0.9999
no APC vs. no APC_AXIN M3	0.5210	0.002445 to 1.040	Yes	*	0.0481
no APC vs. APC WT_AXIN M3	-0.5538	-1.072 to -0.03516	Yes	*	0.0289
no APC vs. APC ΔCID_AXIN M3	-0.5814	-1.100 to -0.06279	Yes	*	0.0187
no APC vs. APC T1556*_AXIN M3	0.3520	-0.1666 to 0.8706	No	ns	0.5659
no APC vs. APC Q1338*_AXIN M3	0.3142	-0.2044 to 0.8328	No	ns	0.9293
no APC vs. APC S811*_AXIN M3	0.6456	0.1270 to 1.164	Yes	**	0.0067
APC WT vs. APC WT_AXIN M3	0.8526	0.3340 to 1.371	Yes	***	0.0002
APC ΔCID vs. APC ΔCID_AXIN M3	0.7806	0.2620 to 1.299	Yes	***	0.0008
APC T1556* vs. APC T1556*_AXIN M3	0.9269	0.4083 to 1.446	Yes	****	<0.0001
APC Q1338* vs. APC Q1338*_AXIN M3	0.8621	0.3435 to 1.381	Yes	***	0.0002
APC S811* vs. APC S811*_AXIN M3	0.6295	0.1109 to 1.148	Yes	**	0.0087

Table S8. One-way ANOVA analysis corresponding to Figure 4D and STAR Methods (Quantification and statistical analysis).

Bonferroni's multiple comparisons test	Mean diff.	95.00% CI of diff.	Significant?	Summary	Adjusted P value
no APC vs. APC WT	-5.506	-6.817 to -4.195	Yes	****	<0.0001
no APC vs. APC SAMP mut.	-1.993	-3.304 to -0.6820	Yes	***	0.0009
no APC vs. APC T1556*	-1.944	-3.255 to -0.6332	Yes	**	0.0012
no APC vs. APC 20R mut.	-4.255	-5.566 to -2.944	Yes	****	<0.0001
no APC vs. no APC_AXIN M3	0.4914	-0.8195 to 1.802	No	ns	>0.9999
no APC vs. APC WT_AXIN M3	-2.477	-3.788 to -1.166	Yes	****	<0.0001
no APC vs. APC SAMP mut. AXIN_M3	0.09441	-1.217 to 1.405	No	ns	>0.9999
no APC vs. APC T1556*_AXIN M3	0.2288	-1.082 to 1.540	No	ns	>0.9999
no APC vs. APC 20R mut._AXIN M3	-1.044	-2.355 to 0.2667	No	ns	0.2290
APC WT vs. APC WT_AXIN M3	3.028	1.717 to 4.339	Yes	****	<0.0001
APC SAMP mut. Vs. APC SAMP mut. Axin_M3	2.087	0.7764 to 3.398	Yes	***	0.0005
APC T1556* vs. APC T1556*_AXIN M3	2.173	0.8620 to 3.484	Yes	***	0.0003
APC 20R mut. Vs. APC 20R mut._AXIN M3	3.211	1.900 to 4.522	Yes	****	<0.0001
APC WT vs. APC 20R mut.	1.251	-0.06032 to 2.562	No	ns	0.0710
APC WT_M3 vs. APC 20R mut._AXIN M3	1.433	0.1223 to 2.744	Yes	*	0.0244

Table S9. One-way ANOVA analysis corresponding to Figure S5D, which is related to Figure 4, and STAR Methods (Quantification and statistical analysis).

Bonferroni's multiple comparisons test	Mean diff.	95.00% CI of diff.	Significant?	Summary	Adjusted P value
no APC vs. APC WT	-1.528	-1.900 to -1.156	Yes	****	<0.0001
no APC vs. APC SAMP mut.	-0.5513	-0.9235 to -0.1791	Yes	**	0.0012
no APC vs. APC T1556*	-0.5072	-0.8794 to -0.1350	Yes	**	0.0030
no APC vs. APC 20R mut.	-1.337	-1.709 to -0.9644	Yes	****	<0.0001
no APC vs. no APC_AXIN M3	0.3765	0.004260 to 0.7487	Yes	*	0.0458
no APC vs. APC WT_AXIN M3	-0.8799	-1.252 to -0.5077	Yes	****	<0.0001
no APC vs. APC SAMP mut. AXIN_M3	0.04048	-0.3317 to 0.4127	No	ns	>0.9999
no APC vs. APC T1556*_AXIN M3	0.1024	-0.2698 to 0.4746	No	ns	>0.9999
no APC vs. APC 20R mut._AXIN M3	-0.4596	-0.8318 to -0.08735	Yes	**	0.0081
APC WT vs. APC WT_AXIN M3	0.1911	-0.1811 to 0.5633	No	ns	>0.9999
APC SAMP mut. vs. APC SAMP mut. Axin_M3	0.4204	0.04817 to 0.7926	Yes	*	0.0184
APC T1556* vs. APC T1556*_AXIN M3	0.6478	0.2756 to 1.020	Yes	***	0.0002
APC 20R mut. vs. APC 20R mut._AXIN M3	0.5918	0.2196 to 0.9640	Yes	***	0.0005
APC WT vs. APC 20R mut.	0.6096	0.2374 to 0.9818	Yes	***	0.0004
APC WT_M3 vs. APC 20R mut._AXIN M3	0.8771	0.5049 to 1.249	Yes	****	<0.0001

Table S10. Individual expression constructs for DC subunits. UniProt IDs (UniProt Consortium, 2021) are provided. (related to Figure 1B, 2A-B, 3A-C, S5E, S7A-B)

insert in pLIB vector	UniProt ID	tag	mutations
APC	P25054 APC_ HUMAN	N-term	
APC T1156*		N-term	Based on COLO-205 cell line, APC p.T1556Nfs*3 mutation, which replaces the T1556 codon with codons for N, Y and stop (*).
APC Q1338*		N-term	Based on SW480 cell line, harbouring a nonsense point mutation at the Q1338 codon.
APC S811*		N-term	Based on COLO320-DM cell line, harbouring a nonsense point mutation at the S811 codon.
APC ΔCID		N-term	S1404 – G1466 deletion
APC SAMP mutant		N-term	SAMP1: I1579A, M1583A, P1584A<br>SAMP2: I1728A, M1732A, P1733A<br>SAMP3: I2043A, M2047A, P2048A<br><br>Mutations were designed based on the crystal structure of the human AXIN1 RGS domain bound to the human APC SAMP3 repeat (Spink et al., 2000; PDB: 1EMU).
APC 20R phospho mutant		N-term	20R1: S1272A, S1275A, S1276A, S1278A, S1279A, S1281A<br>20R2: S1385A, T1388A, S1389A, S1391A, S1392A<br>20R3: S1501A, S1504A, S1505A, S1507A, S1510A<br>20R4: S1652A, T1655A, S1656A, S1658A, T1661A<br>20R5: S1857A, S1861A, S1863A, S1864A<br>20R6: S1967A, S1968A, S1970A, S1971A, S1973A<br>20R7: S2022A, S2025A, S2026A, S2028A, S2029A, S2031A<br><br>Mutations were designed based on Ha et al., 2004, which reports the crystal structure of phosphorylated 20R3 from human APC bound to the armadillo repeat region of murine β-catenin (PDB: 1V18).
AXIN1	O15169 AXIN1_ HUMAN	C-term	
AXIN1 M3		C-term	I794A, R797D (Fiedler et al., 2011)
β-catenin	P35222 CTNB1_ HUMAN	N-term	
β-catenin-4A phospho-mutant		N-term	S33A, S37A, T41A, S45A
β-catenin -ybbR		N-term	C-terminal ybbR; *-linker (GGGS)* – DSLEFIASKLA * stop
GSK3β	P49841 GSK3B_ HUMAN		
GSK3β kinase-dead			K85A, D181N (PDB: 1H8F; Dajani et al., 2001)
CK1α	P48729 KC1A_ HUMAN		
β-TrCP	Q9UKB1 FBW1B_ HUMAN		
CUL1	Q13616 CUL1_ HUMAN	N-term	
SKP1	P63208		

	SKP1_ HUMAN		
RBX1	P62877 RBX1_ HUMAN		

Table S11. Purified recombinant protein complexes. (related to Figure 1B, 2A-B, 3A-C, S5E)

Construct	Description
AXIN1-kinase complex	pBIG1a vector containing: AXIN1-TEV-dStrepII, CK1α, GSK3β
AXIN1-kinase GSK3β-dead complex	pBIG1a vector containing: AXIN1-TEV-dStrepII, CK1α, GSK3β K85A, D181N
AXIN1 M3-kinase complex	pBIG1a vector containing: AXIN1 M3-TEV-dStrepII, CK1α, GSK3β
AXIN1 M3-kinase GSK3β-dead complex	pBIG1a vector containing: AXIN1 M3-TEV-dStrepII, CK1α, GSK3β K85A, D181N
AXIN1-kinase β-catenin complex	pBIG1a vector containing: AXIN1-TEV-dStrepII, β-catenin, CK1α, GSK3β
wild-type destruction complex (DC)	pBIG2ab vector containing: APC, AXIN1-TEV-dStrepII, β-catenin, CK1α, GSK3β
DC – AXIN1 M3	pBIG2ab vector containing: APC, AXIN1 M3-TEV-dStrepII, β-catenin, CK1α, GSK3β
DC – APC T1556*	pBIG2ab vector containing: APC T1556*, AXIN1-TEV-dStrepII, β-catenin, CK1α, GSK3β
DC – APC Q1338*	pBIG2ab vector containing: APC Q1338*, AXIN1-TEV-dStrepII, β-catenin, CK1α, GSK3β
DC – APC S811*	pBIG2ab vector containing: APC S811*, AXIN1-TEV-dStrepII, β-catenin, CK1α, GSK3β
$SCF^{β\text{-}TrCP}$ E3 ubiquitin ligase	pBIG2ab vector containing: β-TrCP, dStrepII-TEV-CUL1, SKP1, RBX1
